# A study on selective transformation of norbornadiene into fluorinated cyclopentane-fused isoxazolines

**DOI:** 10.3762/bjoc.17.132

**Published:** 2021-08-13

**Authors:** Zsanett Benke, Attila M Remete, Loránd Kiss

**Affiliations:** 1 Institute of Pharmaceutical Chemistry, University of Szeged, H-6720 Szeged, Eötvös u. 6, Hungary; 2 University of Szeged, Interdisciplinary Excellence Centre, Institute of Pharmaceutical Chemistry, H-6720 Szeged, Eötvös u. 6, Hungary

**Keywords:** functionalization, metathesis, nitrile oxide, organofluorine chemistry, selectivity

## Abstract

This work presents an examination of the selective functionalization of norbornadiene through nitrile oxide 1,3-dipolar cycloaddition/ring-opening metathesis (ROM)/cross-metathesis (CM) protocols. Functionalization of commercially available norbornadiene provided novel bicyclic scaffolds with multiple stereogenic centers. The synthesis involved selective cycloadditions, with subsequent ROM of the formed cycloalkene-fused isoxazoline scaffolds and selective CM by chemodifferentiation of the olefin bonds of the resulting alkenylated derivatives. Various experimental conditions were applied for the CM transformations with the goal of exploring substrate and steric effects, catalyst influence and chemodifferentiation of the olefin bonds furnishing the corresponding functionalized, fluorine-containing isoxazoline derivatives.

## Introduction

Olefin metathesis is considered to be a powerful synthetic tool for the creation of olefin bonds [1]. Several types of metathesis reactions, such as ring-opening metathesis (ROM), cross-metathesis (CM), ring-closing metathesis (RCM) or ring-opening/cross-metathesis (ROCM) have found high utility in the creation of C=C bonds and in the synthesis of a number of organic molecules, functionalized scaffolds or various building blocks. The efficient catalytic activity and remarkable functional group tolerance of commercially available versatile Ru-based olefin metathesis catalysts have allowed wide applicability of these transformations [1–8]. Moreover, the robustness of many commercial Ru-based catalysts has enabled the general application of olefin metathesis in the synthesis of versatile functionalized heterocycles [9–11], a wide variety of natural products (especially macrocycles) [12], alkaloids [13], amino acids and functionalized biomolecules such as peptides [14–20] or various drugs [21]. Due to the ring strain, bicyclic systems and derivatives, such as norbornadiene derivatives can easily be converted across ROM or ROCM into a variety of alkenylated, functionalized scaffolds [22–34]. Although metathesis is a reversible process, it is often shifted towards a certain direction. For example, the equilibrium of the reaction of ethylene with a strained ring system and the corresponding ROM product is shifted towards ROM because ring strain disfavors reclosing of the ring system. Therefore, functionalized norbornenes, which are highly strained scaffolds, easily provide a number of functionalized cyclopentanes across the ROM/CM process.

It is well known that the structure of a certain metathesis substrate, the nature of the catalyst and all experimental conditions may highly influence metathesis reactions and determine the outcome of olefin metathesis. The accurate prediction of a specific catalyst, including its efficiency, the suitable experimental conditions such as catalyst loading, temperature, solvent, reaction time or even work-up seem to be a difficult task. It is observed that there is no single universal catalyst suitable for all types of metathesis reactions, and there is no general relationship between the structure of the substrate and the type of catalyst. These assumptions might be valid, in particular, for selective processes such as selection between the olefin bonds by chemodifferentiation or chemodiscrimination [1–8,22].

Since the nature of the substrate, catalyst as well as reaction conditions affect the outcome of metathesis, various publications were dedicated to studies describing selective CM or ROCM transformations. The accurate prediction of selectivity regarding CM reactions is still considered to be a challenging issue among synthetic organic chemists. Of numerous factors contributing to the observed selectivities in metathesis reactions, H-bonding interactions between chloride ligands as H-bond acceptors and OH or NH functions in the metathesis intermediate appear to be determining [35–36].

Selectivity derived from chelation is considered to be an another important contributor. Through the formation of intermediates with stable (e.g., six-membered) chelate ring systems, the chelation ability of oxygen functionalities to ruthenium during metathesis can greatly influence the outcome of the CM reaction [36–37]. Steric factors are another important phenomenon, which will possibly contribute to the selectivity of olefin bonds during a CM reaction [38–43].

Investigations of various types of olefins in CM, such as substituted and functionalized styrenes, unsaturated tertiary alcohols, olefins with quaternary carbon centers, acrylates, allyl ethers or allyl acetates gave a general model suitable for the prediction of product selectivity and olefin bond chemodifferentiation in cross metathesis. In general, regarding the reactivity of the olefin bond in CM, alkenes can be categorized by the relative ability to undergo homodimerization via CM and the possibility of the corresponding homodimers for novel secondary metathesis reactions [44]. Thus, olefins can be categorized as type I (fast homodimerization), type II (slow homodimerization), type III (no homodimerization) and type IV (unreactive olefins, spectators to CM) [44].

Although olefins with perfluorinated alkane moiety are considered as to be of type II, only a handful of literature data are available on the behavior of fluorine-containing olefins or perfluorinated alkenes. The incorporation of fluoroalkyl moieties (such as difluoromethyl, trifluoromethyl and perfluoroalkyl groups) into an organic molecule can often enhance the pharmacokinetic properties of lead candidates in drug research through the improvement in lipophilicity, absorption, distribution, hydrophobicity and metabolism. Considering the high importance of organofluorine chemistry and that of fluoroalkyl groups in pharmaceutical chemistry, a wide range of novel and efficient protocols for the introduction of fluorinated scaffolds or fluoroalkyl groups onto organic molecular entities represent a hot topic in synthetic organic chemistry [45–48]. In order to prepare a certain fluorinated organic molecule, two common approaches are used: i) late-stage fluorination, when the fluorine atom is incorporated in the final step of the synthetic protocol (e.g., deoxofluorinations) or ii) application of various commercial fluorine-containing scaffolds (e.g., fluorine-containing amines, fluorine-containing alkenes etc.) [49–58]. It is to be noted that a recent review has been devoted to the synthesis of various fluorine-containing derivatives through various metathesis techniques by the application of versatile fluorinated substrates [59].

## Results and Discussion

**Figure 1:**
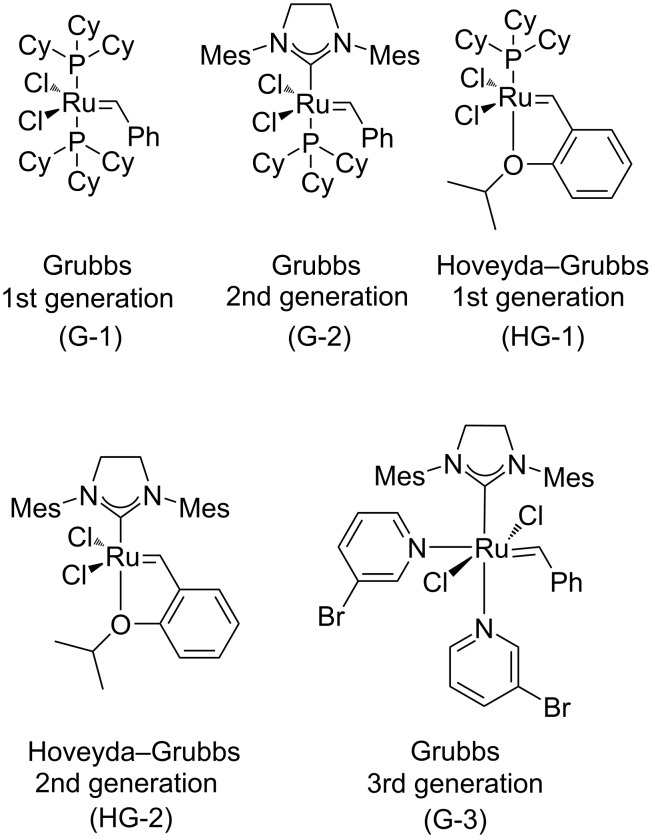
Some commercial Ru-based catalysts used in the current work.

The aim of the current work was to investigate the selective functionalization of readily available norbornadiene across nitrile oxide cycloaddition/ROM/CM protocols in view of the access of various fluorine-containing molecular entities as well as to explore the chemical behavior of olefin bonds in the reaction with some fluorinated alkene derivatives in view of chemodifferentiation. The reactions were performed with various olefin metathesis catalysts to find the most optimal conditions (Figure 1).

**Figure 2:**
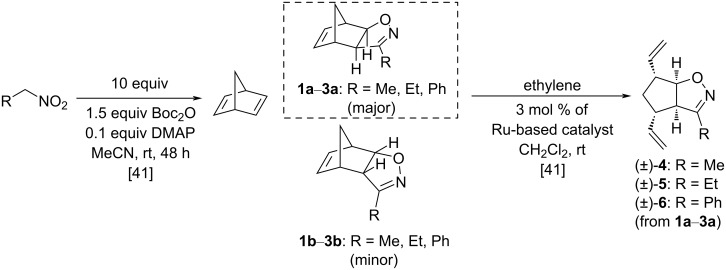
Synthesis of divinylated cyclopentane-fused isoxazolines [41].

The starting divinyl-substituted bicyclic isoxazolines were synthesized according to literature methods, as shown in Figure 2, utilizing nitrile oxide cycloaddition according to the Mukaiyama method followed by ROM of the major product. All five catalysts provided the desired products to some extent, but HG-1 gave the highest yield of (±)-**4**: 76%, (±)-**5**: 75% and (±)-**6**: 87% [41].

**Figure 3:**
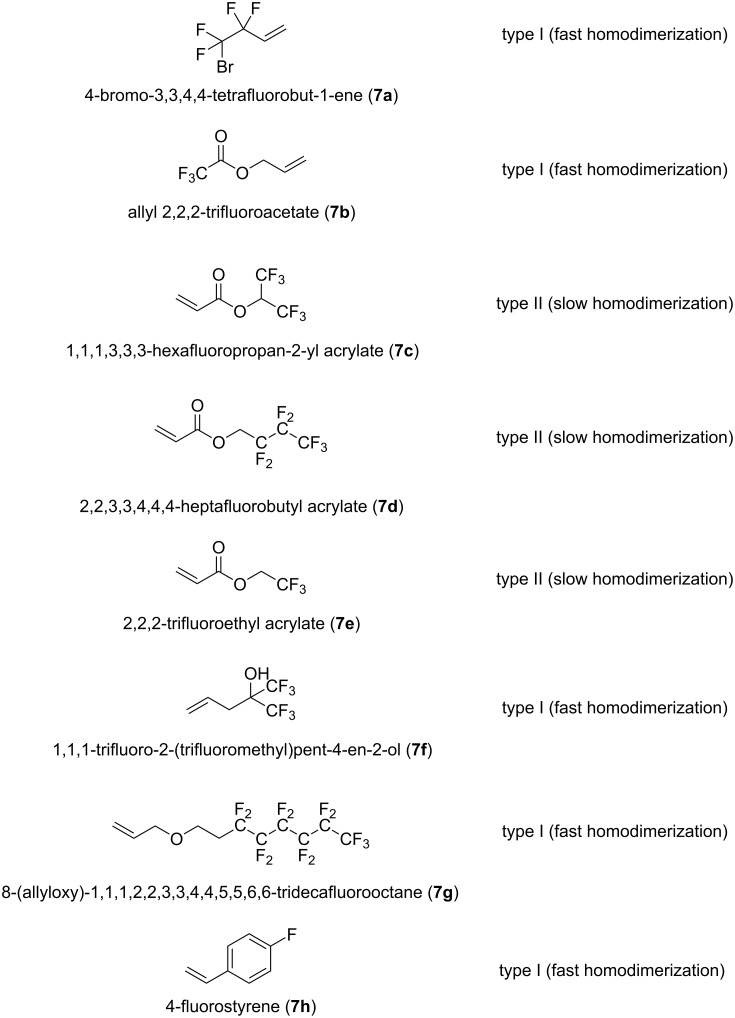
Various fluorine-containing olefins used in the current work.

Further functionalization of compounds (±)-**4**–**6** was attempted via CM with a high number of fluorine-containing alkenes (Figure 3). Compounds **7a** and **7b** as well as **7f**–**h** were type I olefins, while acrylate esters **7c**–**e** were type II olefins. Because 1st generation metathesis catalysts usually perform poorly in CM reactions with acrylates [31–32,36,39,41], only G-2, HG-2 and G-3 were used in our CM steps.

**Scheme 1:**
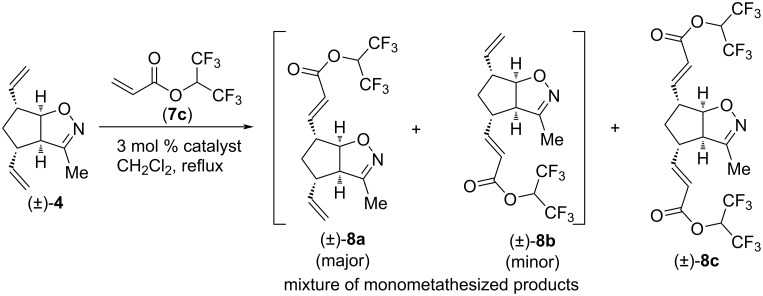
Cross-metathesis of divinylated isoxazoline (±)-**4** with 1,1,1,3,3,3-hexafluoropropan-2-yl acrylate (**7c**).

CM reactions of compound (±)-**4** were investigated first. With 4-bromo-3,3,4,4-tetrafluorobut-1-ene and allyl 2,2,2-trifluoroacetate, no CM product was observed. However, CM reactions with 1,1,1,3,3,3-hexafluoropropan-2-yl acrylate (**7c**) were successful (Scheme 1 and Table 1). When catalyst HG-2 was used (Table 1, entries 1 and 2), decomposition of the catalyst with NaHCO_3_ in aqueous MeOH during workup improved the yield. Therefore, catalyst decomposition was incorporated into the workup procedure of all other CM reactions.

**Table 1:** CM of isoxazoline (±)-**4** with 1,1,1,3,3,3-hexafluoropropan-2-yl acrylate (**7c**).

entry	catalyst	reaction time	yield and ratio of (±)-**8a** and (±)-**8b**	yield of (±)-**8c**

1	HG-2	5 h	5% (3.3:1)	16%^a^
2	HG-2	5 h	10% (3.3:1)	17%
3	G-3	5 h	15% (2.5:1)	0%
4	G-2	5 h	25% (2.5:1)	0%

^a^MeOH, H_2_O/NaHCO_3_ were not added before concentration of the mixture. Note: all yields reported in tables are isolated yield values.

The outcome of the reaction between (±)-**4** and **7c** was catalyst-dependent (Table 1). In the presence of HG-2, the main product was dicoupled (±)-**8c** accompanied by the unseparable mixture of monocoupled products (±)-**8a** and (±)-**8b**. Despite the partial signal overlap, 2D NMR analysis of the (±)-**8a** and (±)-**8b** mixture was possible, and the structure of both (±)-**8a** and (±)-**8b** as well as the compound ratio could be determined. When catalyst G-2 or G-3 was applied for the CM reaction, only monocoupled products were formed. Notably, G-2 or G-3 catalysts had lower selectivity towards (±)-**8a** (ratio of (±)-**8a** and (±)-**8b**: 3.3:1 with HG-2 and 2.5:1 with G-2 or G-3), but they, in particular G-2, provided a superior combined yield of (±)-**8a** and (±)-**8b**.

Note, that although the yield of the CM is relatively low, a full conversion of the starting isoxazoline could be detected. However, all CM transformations alongside the desired coupled compounds afforded a significant amount of unidentifiable polymeric material.

**Scheme 2:**
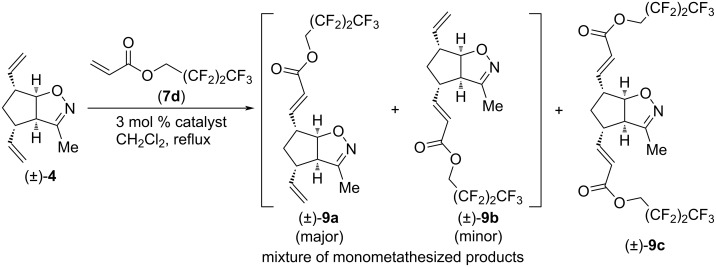
Cross-metathesis of divinylated isoxazoline (±)-**4** with 2,2,3,3,4,4,4-heptafluorobutyl acrylate (**7d**).

CM reactions of compound (±)-**4** with 2,2,3,3,4,4,4-heptafluorobutyl acrylate (**7d**) were also successful (Scheme 2 and Table 2). Again, the catalyst HG-2 provided mainly the dimetathesized product. CM in the presence of G-2 or G-3 gave mainly an inseparable mixture of monometathesized products, but some dimetathesized (±)-**9c** was formed too. Similar to Table 1, G-2 provided the highest combined yield of (±)-**9a** and (±)-**9b**, but it was the least regioselective (ratio of (±)-**9a** and (±)-**9b**: 3.3:1 with HG-2, 2.5:1 with G-3 and 2:1 with G-2).

**Table 2:** CM of isoxazoline (±)-**4** with 2,2,3,3,4,4,4-heptafluorobutyl acrylate (**7d**).

entry	catalyst	reaction time	yield and ratio of (±)-**9a** and (±)-**9b**	yield of (±)-**9c**

1	HG-2	5 h	20% (3.3:1)	34%
2	G-3	5 h	11% (2.5:1)	trace
3	G-2	5 h	36% (2:1)	10%

**Scheme 3:**
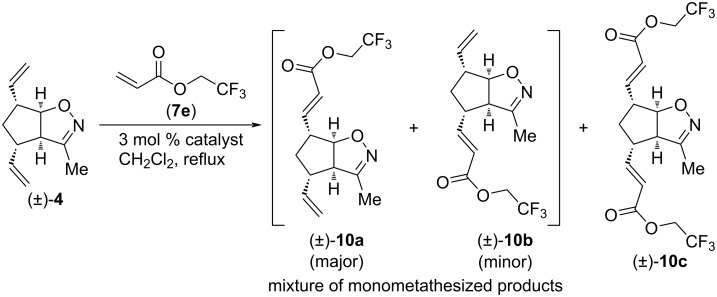
Cross-metathesis of divinylated isoxazoline (**±**)**-4** with 2,2,2-trifluoroethyl acrylate (**7e**).

We continued our investigation with CM reactions of compound (±)-**4** with 2,2,2-trifluoroethyl acrylate (**7e**, Scheme 3 and Table 3). Interestingly, even with HG-2 catalyst, dicoupled product (±)-**10c** formed only in trace amounts. With G-2 and G-3 catalysts, only monocoupled products (±)-**10a** and (±)-**10b** were formed as an inseparable mixture. Similar to Table 1, HG-2 provided the lowest combined yield of monocoupled products, but it had the highest regioselectivity (ratio of (±)-**10a** and (±)-**10b**: 3.3:1 with HG-2 and 1.66:1 with G-2 and G-3, respectively). The highest yield for the (±)-**10a** and (±)-**10b** mixture (23%) was achieved with G-2 catalyst.

**Table 3:** CM of isoxazoline (±)-**4** with 2,2,2-trifluoroethyl acrylate (**7e**).

entry	catalyst	reaction time	yield and ratio of (±)-**10a** and (±)-**10b**	yield of (±)-**10c**

1	HG-2	5 h	15% (3.3:1)	trace
2	G-3	5 h	19% (1.66:1)	0%
3	G-2	5 h	23% (1.66:1)	0%

**Scheme 4:**
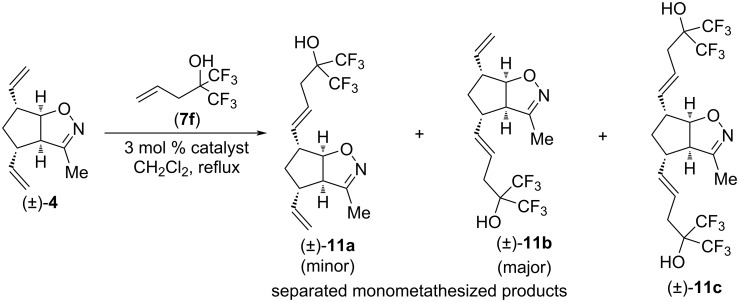
Cross-metathesis of divinylated isoxazoline (±)-**4** with 1,1,1-trifluoro-2-(trifluoromethyl)pent-4-en-2-ol (**7f**).

Then, substrate (±)-**4** was subjected to CM with type I olefin **7f** utilizing HG-2 and G-2 catalysts (Scheme 4 and Table 4). As shown in Tables 1–3, G-3 gave similar or slightly inferior yield compared to G-2. To our surprise, dimetathesized product (±)-**11c** was not formed, and the two monometathesized products were separable. Interestingly, regioselectivity was reversed compared to those in Tables 1–3: the main product was (±)-**11b** (26% with HG-2, 25% with G-2), while isomeric product (±)-**11a** was formed in lower yield (15% with HG-2 and 10% with G-2). G2 catalyst showed better regioselectivity (ratio of (±)-**11a** and (±)-**11b**: 1:1.73 with HG-2 and 1:2.5 with G-2).

**Table 4:** CM of isoxazoline (±)-**4** with 1,1,1-trifluoro-2-(trifluoromethyl)pent-4-en-2-ol (**7f**).

entry	catalyst	reaction time	yield of (±)-**11a**	yield of (±)-**11b**	yield of (±)-**11c**

1	HG-2	5 h	15%	26%	0%
2	G-2	5 h	10%	25%	0%

**Scheme 5:**
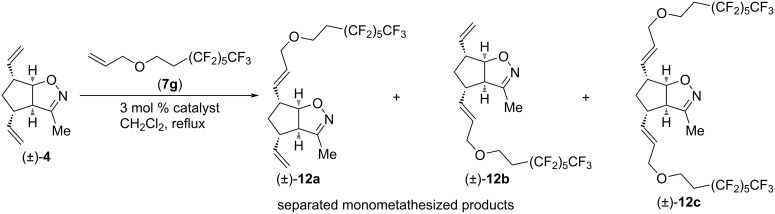
Cross-metathesis of divinylated isoxazoline (±)-**4** with 8-(allyloxy)-1,1,1,2,2,3,3,4,4,5,5,6,6-tridecafluorooctane (**7g**).

Next, CM reactions of isoxazoline (±)-**4** with type I olefin **7g** were studied (Scheme 5 and Table 5) applying HG-2 and G-2 catalysts. Under these conditions, dicoupled product (±)-**12c** was not detected. The formed monocoupled products (±)-**12a** and (±)-**12b** were separable. HG-2 provided both (±)-**12a** and (±)-**12b** in better yield. Interestingly, (±)-**12a** was the main product with HG-2, and (±)-**12b** with G-2.

**Table 5:** CM of isoxazoline (±)-**4** with 8-(allyloxy)-1,1,1,2,2,3,3,4,4,5,5,6,6-tridecafluorooctane (**7g**).

entry	catalyst	reaction time	yield of (±)-**12a**	yield of (±)-**12b**	yield of (±)-**12c**

1	HG-2	5 h	9%	6%	0%
2	G-2	5 h	3%	5%	0%

**Scheme 6:**
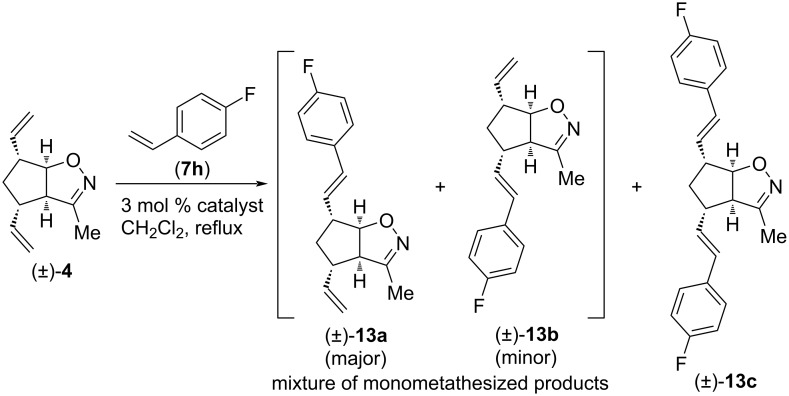
Cross-metathesis of divinylated isoxazoline (±)-**4** with 4-fluorostyrene (**7h**).

In the final test of compound (±)-**4**, it was subjected to CM with 4-fluorosryrene (**7h**), a type I olefin (Scheme 6 and Table 6). With HG-2 catalyst, the main product was dicoupled (±)-**13c**, while the minor product was an inseparable mixture of monocoupled products (±)-**13a** and (±)-**13b**. When G-2 or G-3 catalyst, respectively, was applied for the CM reaction, the major product mixture was (±)-**13a** and (±)-**13b** (the best yield was achieved with G-2) accompanied by some (±)-**13c**. Interestingly, regioselectivity of all three catalysts was the same, with a 1.4:1 ratio of (±)-**13a** and (±)-**13b**.

**Table 6:** CM of isoxazoline (±)-**4** with 4-fluorostyrene (**7h**).

entry	catalyst	reaction time	yield and ratio of (±)-**13a** and (±)-**13b**	yield of (±)-**13c**

1	HG-2	5 h	18% (1.4:1)	30%
2	G-3	5 h	28% (1.4:1)	5%
3	G-2	5 h	36% (1.4:1)	12%

**Scheme 7:**
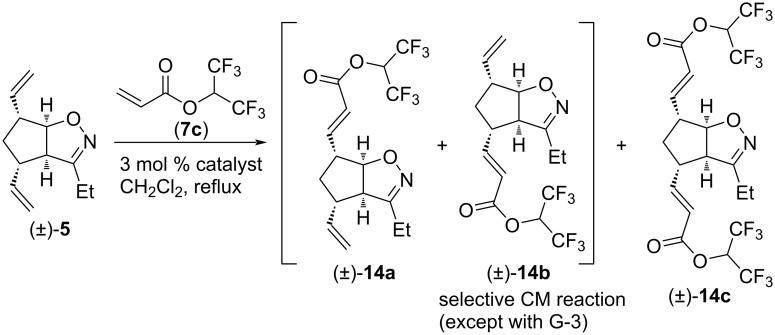
Selective CM of divinylated isoxazoline (±)**-5** with 1,1,1,3,3,3-hexafluoropropan-2-yl acrylate (**7c**).

We continued our work with the study of CM reactions of compound (±)-**5**, which has a slightly longer alkyl chain on the heteroring compared to that of (±)-**4**. Similar to (±)-**4**, no CM product was observed with olefins **7a** and **7b**. In contrast, CM reactions with **7c** were successful (Scheme 7 and Table 7). With HG-2 catalyst, dimetathesized compound (±)-**14c** was the main product, and some monometathesized (±)-**14a** was also formed. With G-2 catalyst, the outcome was the opposite. Interestingly, G-3 catalyst provided only an inseparable mixture of monometathesized products (±)-**14a** and (±)-**14b** (note, that compound (±)-**14b** was not formed in the presence of HG-2 or G-2).

**Table 7:** CM of isoxazoline (±)-**5** with 1,1,1,3,3,3-hexafluoropropan-2-yl acrylate (**7c**).

entry	catalyst	reaction time	yield and ratio of (±)-**14a** and (±)-**14b**	yield of (±)-**14c**

1	HG-2	5 h	2% (1:0)	27%
2	G-3	5 h	13% (2:1)	0%
3	G-2	5 h	16% (1:0)	7%

**Scheme 8:**
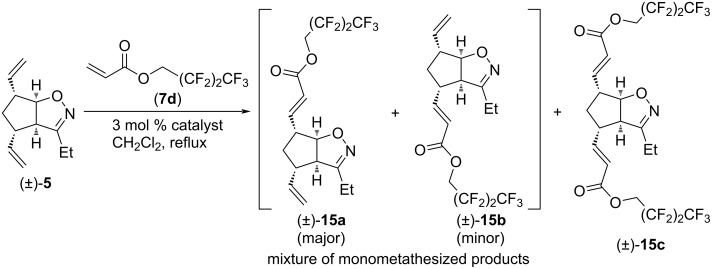
Cross-metathesis of divinylated isoxazoline (±)-**5** with 2,2,3,3,4,4,4-heptafluorobutyl acrylate (**7d**).

CM reactions of isoxazoline (±)-**5** with olefin **7d** in the presence of G-3 catalyst led to the inseparable mixture of monocoupled products (±)-**15a** and (±)-**15b** (Scheme 8 and Table 8). With G-2 catalyst, a mixture of (±)-**15a** and (±)-**15b** was formed in significantly higher yield, and some dicoupled product (±)-**15c** was also isolated from the reaction mixture. With HG-2 catalyst, (±)-**15c** was the main product, but an amount of (±)-**15a** and (±)-**15b** was formed as well. Judging from the ratio of (±)-**15a** and (±)-**15b**, HG-2 catalyst was the most regioselective and G-3 was the least regioselective.

**Table 8:** CM of isoxazoline (±)-**5** with 2,2,3,3,4,4,4-heptafluorobutyl acrylate (**7d**).

entry	catalyst	reaction time	yield and ratio of (±)-**15a** and (±)-**15b**	yield of (±)-**15c**

1	HG-2	5 h	9% (5:1)	42%
2	G-3	5 h	8% (2:1)	0%
3	G-2	5 h	34% (3.3:1)	18%

**Scheme 9:**
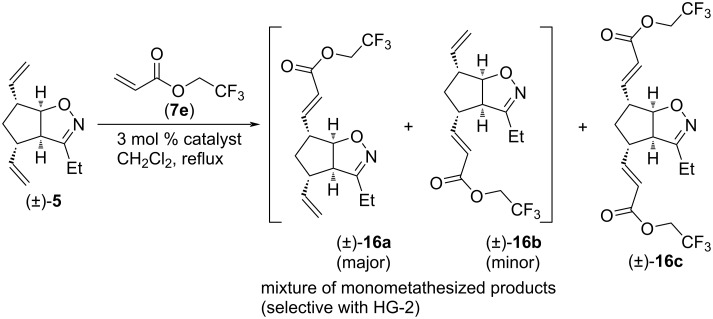
Cross-metathesis of divinylated isoxazoline (±)-**5** with 2,2,2-trifluoroethyl acrylate (**7e**).

The next CM partner was 2,2,2-trifluoroethyl acrylate (**7e**, Scheme 9 and Table 9). With HG-2 catalyst, dimetathesized product (±)-**16c** was formed in medium yield, and some monometathesized product (±)-**16a** was present too. With G-2 catalyst, the inseparable mixture of monometathesized compounds (±)-**16a** and (±)-**16b** (in 2.5:1 ratio) was the main product, accompanied with some (±)-**16c**. Only monometathesized products formed with G-3 catalyst, but both the yield and regioselectivity were inferior compared to those found with G-2.

**Table 9:** CM of isoxazoline (±)-**5** with 2,2,2-trifluoroethyl acrylate (**7e**).

entry	catalyst	reaction time	yield and ratio of (±)-**16a** and (±)-**16b**	yield of (±)-**16c**

1	HG-2	5 h	7% (1:0)	52%
2	G-3	5 h	25% (2:1)	0%
3	G-2	5 h	34% (2.5:1)	8%

**Scheme 10:**
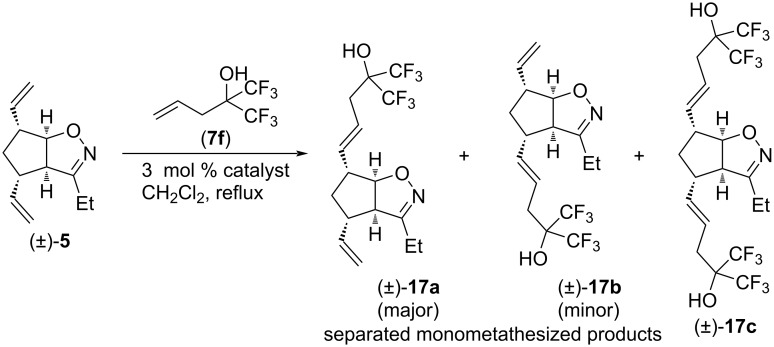
Cross-metathesis of divinylated isoxazoline (±)-**5** with 1,1,1-trifluoro-2-(trifluoromethyl)pent-4-en-2-ol (**7f**).

CM reactions of substrate (±)-**5** and type I olefin **7f** were studied only with HG-2 and G-2 catalysts, respectively (Scheme 10 and Table 10). In both cases, only a mixture of monocoupled products (±)-**17a** and (±)-**17b** was formed, which was separable. The main product was always (±)-**17a**. HG-2 was more regioselective (ratio of (±)-**17a** and (±)-**17b**: 5:1 with HG-2 and 3.3:1 with G2), but G-2 provided a higher yield.

**Table 10:** CM of isoxazoline (±)-**5** with 1,1,1-trifluoro-2-(trifluoromethyl)pent-4-en-2-ol (**7f**).

entry	catalyst	reaction time	yield of (±)-**17a**	yield of (±)-**17b**	yield of (±)-**17c**

1	HG-2	5 h	21%	4%	0%
2	G-2	5 h	34%	11%	0%

**Scheme 11:**
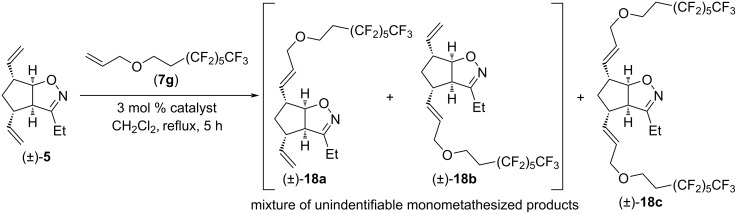
Cross-metathesis of divinylated isoxazoline (±)**-5** with 8-(allyloxy)-1,1,1,2,2,3,3,4,4,5,5,6,6-tridecafluorooctane (**7g**).

We also attempted CM reactions of (±)-**5** with 8-(allyloxy)-1,1,1,2,2,3,3,4,4,5,5,6,6-tridecafluorooctane (**7g**, Scheme 11). However, dimetathesized product (±)-**18c** was not formed, and isolation of monometathesized products (±)-**18a** and (±)-**18b** (or a mixture thereof) in pure form failed despite repeated attempts of chromatographic separation.

**Scheme 12:**
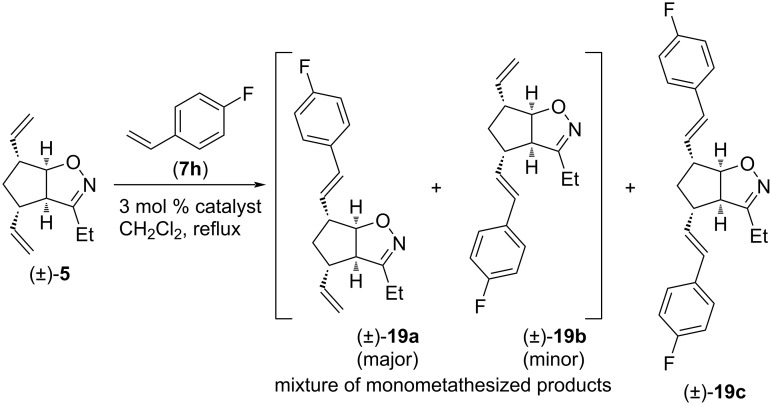
Cross-metathesis of divinylated isoxazoline (±)**-5** with 4-fluorostyrene (**7h**).

Finally, isoxazoline (±)-**5** was subjected to CM with 4-fluorostyrene (**7h**, Scheme 12 and Table 11). HG-2 catalyst provided mainly dicoupled product (±)-**19c**, but some amount of (±)-**19a** and (±)-**19b** mixture was isolated too. G-3 catalyst provided mainly the inseparable mixture of monocoupled products (±)-**19a** and (±)-**19b**, but some (±)-**19c** was formed as well. With G-2 catalyst, a product mixture of (±)-**19a** and (±)-**19b** was formed in slightly higher yield than with G-3, but it was accompanied with a considerable amount of (±)-**19c**. With all three catalysts, the ratio of (±)-**19a** and (±)-**19b** was 2:1.

**Table 11:** CM of isoxazoline (±)**-5** with 4-fluorostyrene (**7h**).

entry	catalyst	reaction time	yield and ratio of (±)-**19a** and (±)-**19b**	yield of (±)-**19c**

1	HG-2	5 h	12% (2:1)	38%
2	G-3	5 h	35% (2:1)	7%
3	G-2	5 h	37% (2:1)	22%

**Scheme 13:**
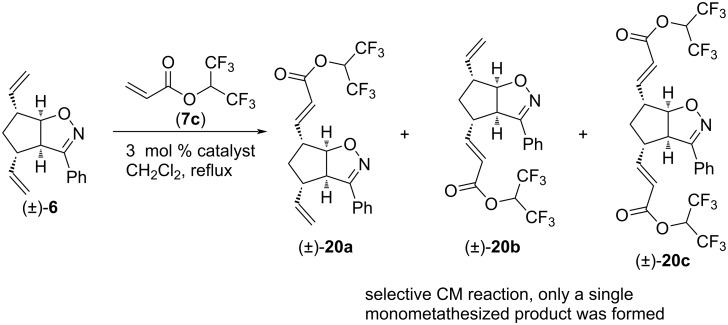
Cross-metathesis of divinylated isoxazoline (±)-**6** with 1,1,1,3,3,3-hexafluoropropan-2-yl acrylate (**7c**).

We continued our work with the study of CM reactions of phenyl-substituted isoxazoline (±)**-6**. Similar to (±)-**4** and (±)-**5**, no CM product was observed with olefins **7a** and **7b**. However, CM reactions with **7c** were successful (Scheme 13 and Table 12). With HG-2 catalyst, dimetathesized compound (±)-**20c** was the main product and monometathesized compound (±)-**20a** was the minor product. With G-2 catalyst, that preference was reversed. G-3 catalyst provided product (±)-**20a** in an exclusive manner, and this was the most efficient way to synthesize this monometathesized compound. Importantly, formation of alternative monometathesized product (±)-**20b** was not observed.

**Table 12:** CM of isoxazoline (±)-**6** with 1,1,1,3,3,3-hexafluoropropan-2-yl acrylate (**7c**).

entry	catalyst	reaction time	yield and ratio of (±)-**20a** and (±)-**20b**	yield of (±)-**20c**

1	HG-2	5 h	6% (1:0)	38%
2	G-3	5 h	30% (1:0)	0%
3	G-2	5 h	16% (1:0)	7%

**Scheme 14:**
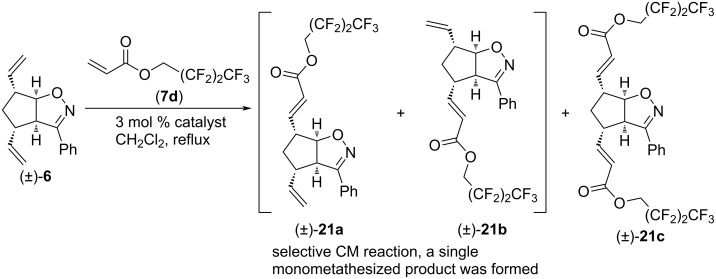
Cross-metathesis of divinylated isoxazoline (±)-**6** with 2,2,3,3,4,4,4-heptafluorobutyl acrylate (**7d**).

Then, CM reactions of (±)-**6** with alkene **7d** were explored (Scheme 14 and Table 13). G-3 catalyst yielded (±)-**21a** as the sole monocoupled product, while HG-2 and G-2 catalysts gave both monocoupled (±)-**21a** and dicoupled (±)-**21c**. The best yield of (±)-**21c** was achieved with HG-2 catalyst (although G-2 catalyst also produced a surprisingly high amount of (±)-**21c**), while the synthesis of (±)-**21a** was the most efficient with G-2 catalyst. Note, that this CM reaction was also regioselective.

**Table 13:** CM of isoxazoline (±)-**6** with 2,2,3,3,4,4,4-heptafluorobutyl acrylate (**7d**).

entry	catalyst	reaction time	yield and ratio of (±)-**21a** and (±)-**21b**	yield of (±)-**21c**

1	HG-2	5 h	18% (1:0)	37%
2	G-3	5 h	17% (1:0)	0%
3	G-2	5 h	27% (1:0)	32%

**Scheme 15:**
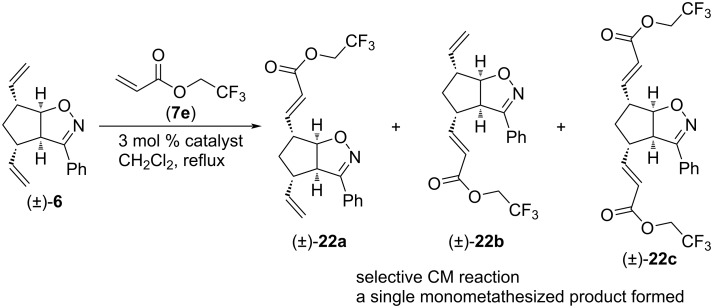
Cross-metathesis of divinylated isoxazoline (±)-**6** with 2,2,2-trifluoroethyl acrylate (**7e**).

CM reactions of compound (±)-**6** with olefin **7e** were also regioselective (Scheme 15 and Table 14). G-3 catalyst yielded only monometathesized (±)-**22a** as a single product, HG-2 catalyst gave mainly dimetathesized (±)-**22c** (together with some (±)-**22a**) and G-2 catalyst gave mainly monometathesized (±)-**22a** (together with some (±)-**22c**). The best yield of (±)-**22c** was achieved with HG-2 catalyst, while the synthesis of (±)-**22a** was the most efficient with G-2 catalyst.

**Table 14:** CM of isoxazoline (±)-**6** with 2,2,2-trifluoroethyl acrylate (**7e**).

entry	catalyst	reaction time	yield and ratio of (±)-**22a** and (±)-**22b**	yield of (±)-**22c**

1	HG-2	5 h	4% (1:0)	48%
2	G-3	5 h	22% (1:0)	0%
3	G-2	5 h	37% (1:0)	11%

**Scheme 16:**
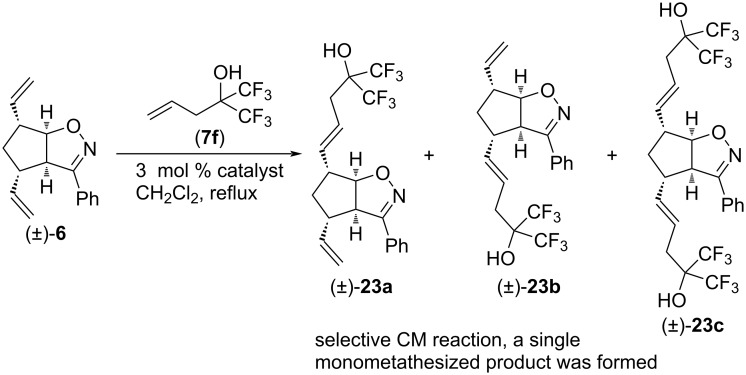
Cross-metathesis of divinylated isoxazoline (±)**-6** with 1,1,1-trifluoro-2-(trifluoromethyl)pent-4-en-2-ol (**7f**).

Cross metathesis of substrate (±)-**6** with unsaturated alcohol **7f** was also performed (Scheme 16 and Table 15). The reaction was completely regioselective with HG-2 and G-2 catalysts, and provided only a single monocoupled product, (±)-**23a**. The best yield was achieved with G-2 catalyst.

**Table 15:** CM of isoxazoline (±)-**6** with 1,1,1-trifluoro-2-(trifluoromethyl)pent-4-en-2-ol (**7f**).

entry	catalyst	reaction time	yield and ratio of (±)-**23a** and (±)-**23b**	yield of (±)-**23c**

1	HG-2	5 h	3% (1:0)	0%
2	G-2	5 h	54% (1:0)	0%

**Scheme 17:**
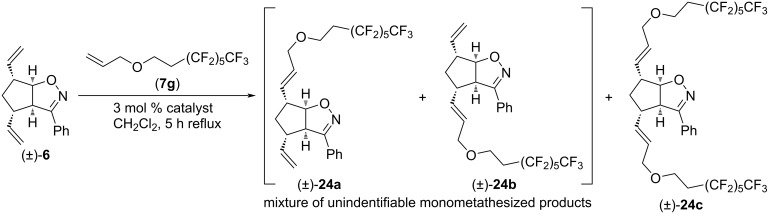
Cross-metathesis of divinylated isoxazoline (±)-**6** with 8-(allyloxy)-1,1,1,2,2,3,3,4,4,5,5,6,6-tridecafluorooctane (**7g**).

We also attempted CM reactions of (±)-**6** with 8-(allyloxy)-1,1,1,2,2,3,3,4,4,5,5,6,6-tridecafluorooctane (**7g**, Scheme 17). However, dimetathesized product (±)-**24c** was not formed, and isolation of monometathesized products (±)-**24a** and (±)-**24b** (or a mixture thereof) in pure form failed, despite repeated attempts of chromatographic separation.

**Scheme 18:**
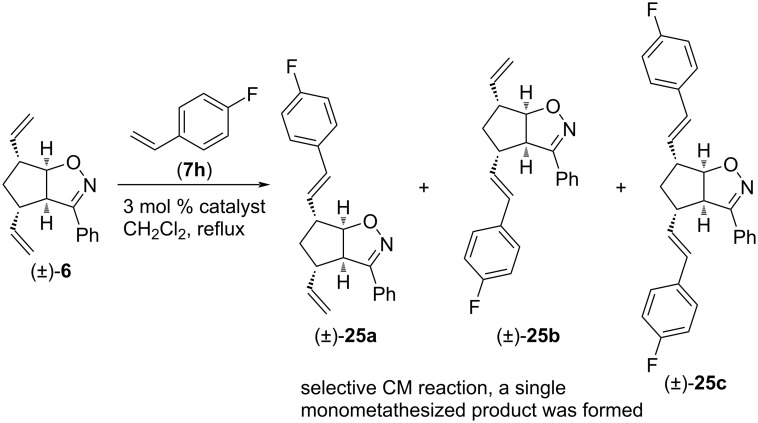
Cross-metathesis of divinylated isoxazoline (±)-**6** with 4-fluorostyrene (**7h**).

Finally, CM reactions between isoxazoline (±)-**6** and 4-fluorostyrene (**7h**) were studied (Scheme 18 and Table 16). HG-2 catalyst provided monocoupled (±)-**25a** and dicoupled (±)-**25c** in a comparable amount. With G-2 catalyst, both products were formed in higher yield. G-3 catalyst provided mostly monocoupled (±)-**25a**, but a low amount of (±)-**25c** was also isolated. Note, that the CM reaction was highly regioselective (alternative monocoupled product (±)-**25b** was not detected).

**Table 16:** CM of isoxazoline (±)-**6** with 4-fluorostyrene (**7h**).

entry	catalyst	reaction time	yield and ratio of (±)-**25a** and (±)-**25b**	yield of (±)-**25c**

1	HG-2	5 h	18% (1:0)	25%
2	G-3	5 h	18% (1:0)	2%
3	G-2	5 h	28% (1:0)	49%

## Conclusion

An insight into the study of selective functionalization of norbornadiene through nitrile oxide 1,3-dipolar cycloaddition/ROM/CM strategies was presented. The stepwise functionalization of norbornadiene across the ring olefin bonds generated fluorine-containing alkenylated cyclopentane-fused isoxazolines. The synthetic protocol was based on selective nitrile oxide cycloaddition to the norbornadiene C=C bond, followed by ROM of the resulting cyclopentane-fused isoxazolines. In the final step, selective CM by chemodifferentiation of the newly created olefin bonds on the resulting alkenylated derivatives took place. As coupling olefin partners in CM reactions, several commercial fluorine-containing alkenes have been investigated (type I and type II), and CM has been studied in order to explore the substrate effect, catalyst influence and the chemical behavior of the olefin bonds. Second-generation Ru-based commercial catalysts G-2 and HG-2 as well as third-generation G-3 were found to be more effective in the CM transformations. Note, that first-generation catalyst HG-1 did not afford cross-metathesized products.

**Figure 4:**
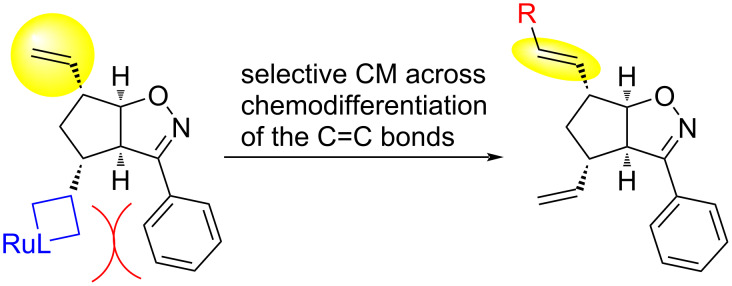
Chemoselective CM reaction due to steric hindrance.

Our data allows to summarize some clearly visible general trends. First of all, most CM reactions of compounds (±)-**4** and (±)-**5** were only slightly regioselective (transformation of the vinyl group at C-6 was preferred, except for the reactions of isoxazoline (±)-**4** with **7f** and **7g**), while all CM reactions of (±)-**6** were completely regioselective (the vinyl group at C-6 was transformed first). This can be explained by steric hindrance: the substituent at C-3 on the isoxazoline ring shields the vinyl group at C-4 from reacting with the bulky catalyst molecules (Figure 4). For the smaller Me or Et groups, this effect is relatively weak (only some reactions of (±)-**5** with **7c** and **7e** were completely regioselective). The large Ph group, however, provided complete regioselectivity in all successful CM reactions.

The ratio of monometathesized products in CM reactions of (±)-**4** and (±)-**5** also depended on the catalyst. Generally, HG-2 catalyst provided the highest regioselectivity. Unfortunately, separation of regioisomeric monometathesized compounds proved to be impossible in most cases (only monocoupled products with olefins **7f** and **7g** were separable). However, the formed dimetathesized products could be separated from the monometathesized compounds.

Usually, the best (combined) yield of monometathesized products was achieved with G-2 catalyst, while HG-2 provided the best yield of dicoupled products. Notably, G-3 catalyst highly disfavored the formation of dimetathesized products.

Further investigations in view of the selectivity of CM reactions with other novel model compounds as well as further functionalization strategies are currently being investigated in our group.

## Supporting Information

File 1 Experimental section and NMR spectra.
