# Supplementary material for: A study on selective transformation of norbornadiene into fluorinated cyclopentane-fused isoxazolines
Source: Beilstein J Org Chem. 2021 Aug 13;17:2051–66. doi: 10.3762/bjoc.17.132 (PMC8372314; doi:10.3762/bjoc.17.132)
Supplement: File 1 — Experimental section and NMR spectra. [file Beilstein_J_Org_Chem-17-2051-s001.pdf]

BEILSTEIN JOURNAL OF ORGANIC CHEMISTRY

Supporting Information

for

A study on selective transformation of norbornadiene into fluorinated cyclopentane-fused isoxazolines

Zsanett Benke, Attila M. Remete and Loránd Kiss

Beilstein J. Org. Chem. **2021,** *17,* 2051–2066. doi:10.3762/bjoc.17.132

Experimental section and NMR spectra

License and Terms: This is a supporting information file under the terms of the Creative Commons Attribution License (https://creativecommons.org/licenses/by/4.0). Please note that the reuse, redistribution and reproduction in particular requires that the author(s) and source are credited and that individual graphics may be subject to special legal provisions.
The license is subject to the *Beilstein Journal of Organic Chemistry* terms and conditions: (https://www.beilstein-journals.org/bjoc/terms)

Experimental section

The experiments were performed in a manner analogous to reference [1].

General procedure for ring-opening metathesis

To a solution of isoxazoline-fused derivative (100 mg) in anhydrous CH_2Cl_2 (15 mL), catalyst (3 mol %, see tables in the main manuscript) and ethylene were added from a bottle by bubbling, and the mixture was stirred for 2 hours at room temperature. The reaction was monitored by TLC and after completion, the catalyst was decomposed by adding a solution of $NaHCO_3$ (0.3 g) into the mixture of methanol (5 mL) and water (25 mL). The reaction mixture was stirred for 2 hours, then 20 mL water was added, and the two layers were separated. The aqueous phase was extracted with CH_2Cl_2 (3 × 30 mL), the combined organic phase was dried over Na_2SO_4 and concentrated under vacuum, and the crude product was purified by means of column chromatography on silica gel (*n*-hexane/EtOAc).

General procedure for cross-metathesis

To a solution of isoxazoline derivative (100 mg) in anhydrous CH_2Cl_2 (15 mL), Ru-based catalyst (3 mol %, see tables in the main manuscript) and fluorinated olefin **7a–h** (10 equiv) were added, and the mixture was stirred for 5 hours at reflux temperature. The reaction was monitored by TLC and after completion, the catalyst was decomposed by adding a solution of $NaHCO_3$ (0.3 g) into the mixture of methanol (5 mL) and water (25 mL). The reaction mixture was stirred for 2 hours, then 20 mL water was added, and the phases were separated. The aqueous phase was extracted with CH_2Cl_2 (3 × 10 mL). The combined organic phase was dried over Na_2SO_4 and concentrated under vacuum, and the crude product was purified by means of column chromatography on silica gel (*n*-hexane/EtOAc).

(*E*)-1,1,1,3,3,3-Hexafluoropropan-2-yl 3-((3a*R,4*S**,6*R**,6a*R**)-3-methyl-4-vinyl-4,5,6,6a-tetrahydro-3aH-cyclopenta[*d*]isoxazol-6-yl)acrylate** and **(*E*)-1,1,1,3,3,3-hexafluoropropan-2-yl 3-((3a*R**,4*S**,6*R**,6a*R**)-3-methyl-6-vinyl-4,5,6,6a-tetrahydro-3a*H*-cyclopenta[*d*]isoxazol-4-yl)acrylate,** (±)-**8a** and (±)-**8b**

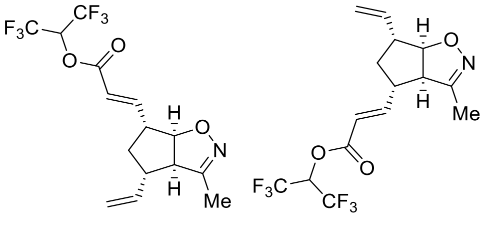

Best catalyst: G-2. Yield of (±)-**8a** and (±)-**8b**: 25% (53 mg); R_f = 0.53 (*n*-hexane/EtOAc 3:1); ratio of (±)-**8a** and (±)-**8b**: 2.5:1.

HRMS calcd. for $C_{15}H_{16}F_6NO_3^+$ ($[M+H]^+$): 372.1028, found: 372.1032.

(2*E*,2'*E*)-Bis(1,1,1,3,3,3-hexafluoropropan-2-yl) 3,3'-((3a*R,4*S**,6*R**,6a*R**)-3-methyl-4,5,6,6a-tetrahydro-3a*H*-cyclopenta[*d*]isoxazole-4,6-diyl)diacrylate,** (±)-**8c**

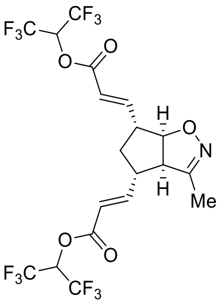

Best catalyst: HG-2. Dark yellow oil; yield: 17% (54 mg); R_f = 0.52 (*n*-hexane/EtOAc 3:1).

^{1}H NMR (500 MHz, $CDCl_3$): δ = 1.63-1.72 (dd, 1H, J = 12 Hz, CH_2); 2.02 (s, 3H, CH_3); 2.15-2.22 (m, 1H, CH_2); 2.87-2.96 (m, 2H, H-4 and H-6); 3.52-3.58 (m, 1H, H-3a); 4.88-4.94 (m, 1H, H-6a); 5.77-5.87 (m, 2H, $CH(CF_3)_2$); 6.04-6.11 (m, 2H, =CH); 7.11-7.25 (m, 2H, =CH).

^{13}C NMR (125 MHz, $CDCl_3$): δ = 11.4, 36.9, 50.7, 60.5, 66.6 (septett, $^2J_{C\text{-}F}$ = 34 Hz, **C**H(CF_3)$_2$), 66.7 (septett, $^2J_{C\text{-}F}$ = 34 Hz, **C**H(CF_3)$_2$), 88.6, 119.0, 119.6, 120.4 (q, $^1J_{C\text{-}F}$=281 Hz, CF_3), 151.6, 153.0, 155.6, 162.2, 162.3.

^{19}F NMR (471 MHz, CDCl$_3$): δ = -73.2 (CF$_3$), -73.3 (CF$_3$).

HRMS calcd. for $C_{19}H_{16}F_{12}NO_5^+$ ($[M+H]^+$): 566.0831, found: 566.0830.

(*E*)-2,2,3,3,4,4,4-Heptafluorobutyl 3-((3a*R,4*S**,6*R**,6a*R**)-3-methyl-4-vinyl-4,5,6,6a-tetrahydro-3a*H*-cyclopenta[*d*]isoxazol-6-yl)acrylate** and **(*E*)-2,2,3,3,4,4,4-hepta-fluorobutyl 3-((3a*R**,4*S**,6*R**,6a*R**)-3-methyl-6-vinyl-4,5,6,6a-tetrahydro-3a*H*-cyclo-penta[*d*]isoxazol-4-yl)acrylate,** (±)-**9a** and (±)-**9b**

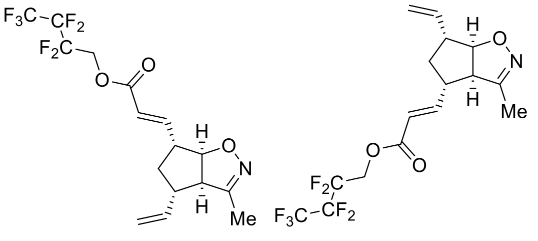

major **minor**

Best catalyst: G-2. Yield of (±)-**9a** and (±)-**9b**: 36% (82 mg); R_f = 0.46 (*n*-hexane/EtOAc 3:1); ratio of (±)-**9a** and (±)-**9b**: 2:1.

HRMS calcd. for $C_{16}H_{17}F_7NO_3^+$ ($[M+H]^+$): 404.1091, found: 404.1092.

(2*E*,2'*E*)-Bis(2,2,3,3,4,4,4-Heptafluorobutyl) 3,3'-((3a*R,4*S**,6*R**,6a*R**)-3-methyl-4,5,6,6a-tetrahydro-3a*H*-cyclopenta[*d*]isoxazole-4,6-diyl)diacrylate,** (±)-**9c**

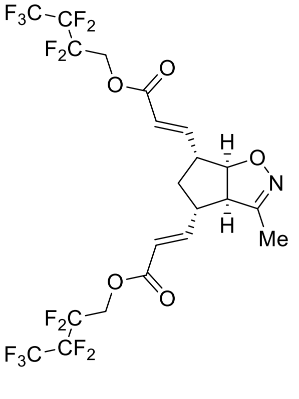

Best catalyst: HG-2. Yellow oil; yield: 34% (120 mg); R_f = 0.36 (*n*-hexane/EtOAc 3:1).

^{1}H NMR (500 MHz, $CDCl_3$): δ = 1.56-1.66 (m, 1H, CH_2); 1.99 (s, 3H, CH_3); 2.10-2.18 (m, 1H, CH_2); 2.82-2.92 (m, 2H, H-4 and H-6); 3.47-3.53 (m, 1H, H-3a); 4.58-4.71 (m, 4H, $CH_2(CF_2)_2CF_3$); 4.83-4.90 (m, 1H, H-6a); 5.97-6.04 (m, 2H, =CH); 6.99-7.13 (m, 2H, =CH).

^{13}C NMR (125 MHz, $CDCl_3$): δ = 11.4, 37.2, 46.6, 50.5, 59.4 (t, *J*=27 Hz, **C**$H_2(CF_2)_2CF_3$), 59.5 (t, *J*=27 Hz, **C**$H_2(CF_2)_2CF_3$), 60.7, 88.9, 100-120 (m, $CF_2CF_2CF_3$), 120.3, 120.8, 149.5, 150.8, 155.7, 163.9,.164.0.

^{19}F NMR (471 MHz, $CDCl_3$): δ = -80.87 (t, *J* = 9 Hz, CF_3), -80.89 (t, *J*=9 Hz, CF_3), -120.35 (m, **CF_2**CF_2CF_3), 127.58 (s, CF_2**CF_2**CF_3).

HRMS calcd. for $C_{21}H_{18}F_{14}NO_5^+$ ($[M+H]^+$): 630.0955, found: 630.0957.

(*E*)-2,2,2-Trifluoroethyl 3-((3a*R,4*S**,6*R**,6a*R**)-3-methyl-4-vinyl-4,5,6,6a-tetrahydro-3a*H*-cyclopenta-[*d*]isoxazol-6-yl)acrylate** and **(*E*)-2,2,2-trifluoroethyl 3-((3a*R**,4*S**,6*R**,6a*R**)-3-methyl-6-vinyl-4,5,6,6a-tetrahydro-3a*H*-cyclopenta[*d*]isoxazol-4-yl)acrylate,** (±)-**10a** and (±)-**10b**

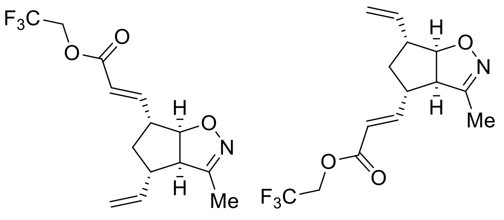

major **minor**

Best catalyst: G-2. Yield of (±)-**10a** and (±)-**10b**: 23% (38 mg); R_f = 0.38 (*n*-hexane/EtOAc 3:1); ratio of (±)-**10a** and (±)-**10b**: 1.66:1; HRMS calcd. for $C_{14}H_{17}F_3NO_3^+$ ($[M+H]^+$): 304.1155, found: 304.1161.

(*E*)-1,1,1-Trifluoro-5-((3a*R,4*S**,6*R**,6a*R**)-3-methyl-4-vinyl-4,5,6,6a-tetrahydro-3a*H*-cyclopenta[*d*]isoxazol-6-yl)-2-(trifluoromethyl)pent-4-en-2-ol,** (±)-**11a**

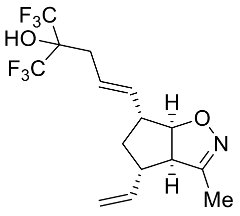

Best catalyst: HG-2. White solid; yield: 15% (30 mg); R_f = 0.36 (*n*-hexane/EtOAc 4:1), mp. 108-109 °C.

^{1}H NMR (500 MHz, $CDCl_3$): δ = 1.36-1.46 (m, 1H, CH_2); 1.92-2.01 (m, 4H, CH_2 and CH_3); 2.63-2.86 (m, 4H, H-4, H-6 and **CH_2**C(CF_3)OH); 3.33-3.41 (m, 1H, H-3a); 4.63-4.69 (m, 1H, H-6a); 4.77 (brs, 1H, OH); 5.06-5.20 (m, 2H, =CH_2); 5.57-5.66 (m, 1H, =CH); 5.69-5.75 (m, 1H, =CH); 5.77-5.87 (m, 1H, =CH).

^{13}C NMR (125 MHz, $CDCl_3$): δ = 11.1, 28.7, 45.8, 48.0, 60.5, 75.7 (septet, $^2J_{C-F}$ = 29 Hz, **C**(CF_3)$_2$OH); 89.7, 115.5, 121.8, 122.9 (q, $^1J_{C-F}$ = 287 Hz, CF_3); 123.3 (q, $^1J_{C-F}$ = 287 Hz, CF_3); 136.1, 139.4, 157.8.

^{19}F NMR (471 MHz, $CDCl_3$): δ = -78.0 (CF_3), -75.3 (CF_3).

HRMS calcd. for $C_{15}H_{18}F_6NO_2^+$ ($[M+H]^+$): 358.1236, found: 358.1240.

(*E*)-1,1,1-Trifluoro-5-((3a*R,4*S**,6*R**,6a*R**)-3-methyl-6-vinyl-4,5,6,6a-tetrahydro-3a*H*-cyclopenta[*d*]isoxazol-4-yl)-2-(trifluoromethyl)pent-4-en-2-ol,** (±)-**11b**

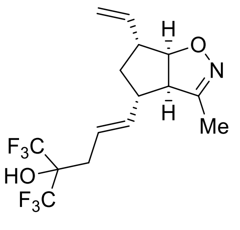

Best catalyst: HG-2. Brown oil; yield: 26% (51 mg); R_f = 0.28 (*n*-hexane/EtOAc 4:1).

^{1}H NMR (500 MHz, $CDCl_3$): δ = 1.36-1.45 (m, 1H, CH_2); 1.92-2.01 (m, 4H, CH_2 and CH_3); 2.57-2.73 (m, 4H, H-4, H-6 and **CH_2**$C(CF_3)_2OH$); 3.31-3.38 (m, 1H, H-3a); 4.11 (brs, 1H, OH); 4.68-4.75 (m, 1H, H-6a); 5.05-5.18 (m, 2H, =CH_2); 5.54-5.92 (m, 3H, =CH).

^{13}C NMR (125 MHz, $CDCl_3$): δ = 11.2, 33.8, 38.3, 48.3, 50.7, 60.9, 75.5 (septet, $^2J_{C-F}$ = 29 Hz, **C**$(CF_3)_2OH$); 89.9, 115.4, 121.8, 123.0 (q, $^1J_{C-F}$ = 286 Hz, CF_3); 138.1, 139.7, 157.2.

^{19}F NMR (471 MHz, $CDCl_3$): δ = -76.3 (CF_3), -76.5 (CF_3).

HRMS calcd. for $C_{15}H_{18}F_6NO_2^+$ ($[M+H]^+$): 358.1236, found: 358.1240.

(3a*R,4*S**,6*R**,6a*R**)-3-Methyl-6-((*E*)-3-((3,3,4,4,5,5,6,6,7,7,8,8,8-tridecafluorooctyl)-oxy)prop-1-en-1-yl)-4-vinyl-4,5,6,6a-tetrahydro-3a*H*-cyclopenta[*d*]isoxazole,** (±)-**12a**

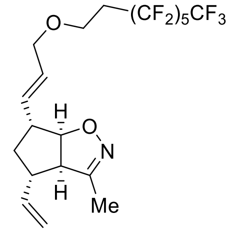

Best catalyst: HG-2. Brown oil; yield: 9% (30 mg); R_f = 0.36 (*n*-hexane/EtOAc 4:1).

^{1}H NMR (500 MHz, $CDCl_3$): δ = 1.37-1.46 (m, 1H, CH_2); 1.93-2.02 (m, 4H, CH_2 and CH_3); 2.37-2.47 (m, 2H, CH_2OCH_2**CH_2**$(CF_2)_5CF_3$); 2.57-2.69 (m, 2H, H-4 and H-6); 3.28-3.34 (m, 1H, H-3a); 3.68-3.74 (t, 2H, *J* = 7 Hz, CH_2O**CH_2**$CH_2(CF_2)_5CF_3$); 3.95-4.00 (m, 2H, **CH_2**$OCH_2CH_2(CF_2)_5CF_3$); 4.68-4.75 (m, 1H, H-6a); 5.02-5.18 (m, 2H, =CH_2); 5.63-5.70 (m, 1H, =CH); 5.76-5.85 (m, 2H, =CH).

^{13}C NMR (125 MHz, $CDCl_3$): δ = 11.4, 31.6 (t, $^2J_{C-F}$=21 Hz, CH_2OCH_2**C**$H_2(CF_2)_5CF_3$); 38.4, 48.4, 50.5, 61.0, 61.8 (t, $^3J_{C-F}$ = 5 Hz, CH_2O**C**$H_2CH_2(CF_2)_5CF_3$); 71.4, 90.1, 100-120 (m, $(CF_2)_5CF_3$); 115.2, 126.9, 133.8, 139.9, 156.6.

^{19}F NMR (471 MHz, $CDCl_3$): δ = -126.0 (CF_2); -123.5 (CF_2); -122.7 (CF_2); 121.7 (CF_2); -113.1 (CF_2); -80.8 (CF_3).

(3a*R,4*S**,6*R**,6a*R**)-3-Methyl-4-((*E*)-3-((3,3,4,4,5,5,6,6,7,7,8,8,8-tridecafluorooctyl)-oxy)prop-1-en-1-yl)-6-vinyl-3a,5,6,6a-tetrahydro-4*H*-cyclopenta[*d*]isoxazole,** (±)-**12b**

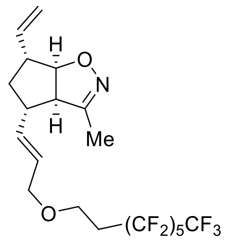

Best catalyst: HG-2. Brown oil; yield: 6% (19 mg); R_f = 0.29 (*n*-hexane/EtOAc 4:1).

^{1}H NMR (500 MHz, $CDCl_3$): δ = 1.37-1.47 (m, 1H, CH_2); 1.94-2.02 (m, 4H, CH_2 and CH_3); 2.36-2.48 (m, 2H, CH_2OCH_2**CH_2**$(CF_2)_5CF_3$); 2.59-2.69 (m, 2H, H-4 and H-6); 3.28-3.35(m, 1H, H-3a); 3.69-3.75 (t, 2H, J = 7 Hz, CH_2O**CH_2**$CH_2(CF_2)_5CF_3$); 3.97-4.00 (d, 2H, J = 5 Hz, **CH_2**$OCH_2CH_2(CF_2)_5CF_3$); 4.70-4.76 (m, 1H, H-6a); 5.04-5.18 (m, 2H, =CH_2); 5.62-5.69 (m, 1H, =CH); 5.69-5.76 (m, 1H, =CH); 5.85-5.94 (m, 1H, =CH).

^{13}C NMR (125 MHz, $CDCl_3$): δ = 11.4, 31.6 (t, $^2J_{C-F}$=21 Hz, CH_2OCH_2**C**$H_2(CF_2)_5CF_3$); 38.3, 47.0, 51.7, 61.2, 61.5 (t, $^3J_{C-F}$ = 5 Hz, CH_2O**C**$H_2CH_2(CF_2)_5CF_3$); 71.1, 90.1, 100-120 (m, $(CF_2)_5CF_3$); 115.5, 126.7, 135.4, 138.2, 156.6.

^{19}F NMR (471 MHz, $CDCl_3$): δ = -126.0 (CF_2); -123.5 (CF_2); -122.7 (CF_2); 121.7 (CF_2); -113.1 (CF_2); -80.8 (CF_3).

HRMS calcd. for $C_{20}H_{21}F_{13}NO_2^+$ ($[M+H]^+$): 554.1359, found: 554.1358.

(3a*R,4*S**,6*R**,6a*R**)-4-((*E*)-4-Fluorostyryl)-3-methyl-6-vinyl-4,5,6,6a-tetrahydro-3a*H*-cyclopenta-[*d*]isoxazole** and **(3a*R**,4*S**,6*R**,6a*R**)-6-((*E*)-4-fluorostyryl)-3-methyl-4-vinyl-4,5,6,6a-tetrahydro-3a*H*-cyclopenta[*d*]isoxazole,** (±)-**13a** and (±)-**13b**

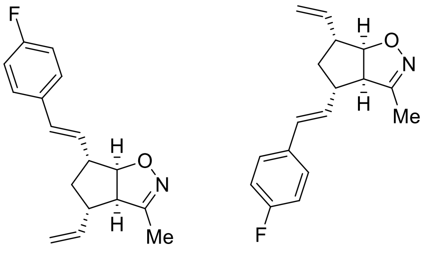

Best catalyst: G-2. Yield of (±)-**13a** and (±)-**13b**: 36% (55 mg); R_f = 0.44 (*n*-hexane/EtOAc 3:1); ratio of (±)-**13a** and (±)-**13b**: 1.4:1.

HRMS calcd. for $C_{17}H_{19}FNO^+$ ($[M+H]^+$): 272.1445, found: 272.1446.

(3a*R,4*S**,6*R**,6a*R**)-4,6-Bis((*E*)-4-fluorostyryl)-3-methyl-4,5,6,6a-tetrahydro-3a*H*-cyclopenta[*d*]isoxazole,** (±)-**13c**

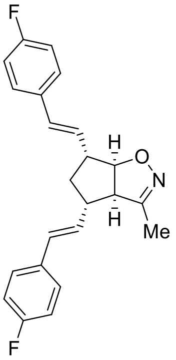

Best catalyst: HG-2. Yellow oil; yield: 30% (61 mg); R_f = 0.35 (*n*-hexane/EtOAc 3:1).

^{1}H NMR (500 MHz, $CDCl_3$): δ = 1.54-1.64 (dd, 1H, *J*=12 Hz, CH_2); 2.00 (s, 3H, CH_3); 2.07-2.14 (m, 1H, CH_2); 2.77-2.87 (m, 2H, H-4 and H-6); 3.40-3.46 (m, 1H, H-3a); 4.81-4.87 (m, 1H, H-6a); 6.07-6.14 (m, 1H, =CH); 6.15-6.22 (m, 1H, =CH); 6.44-6.51 (m, 2H, =CH); 6.95-7.04 (m, 4G, CH-Ar); 7.28-7.35 (m, 4H, CH-Ar).

^{13}C NMR (125 MHz, $CDCl_3$): δ = 11.6, 39.2, 47.9, 51.3, 61.5, 90.3, 115.4 (d, $^2J_{C\text{-}F}$ = 19 Hz); 115.6 (d, $^2J_{C\text{-}F}$ = 19 Hz); 127.6 (d, $^3J_{C\text{-}F}$ = 7 Hz); 127.6 (d, $^3J_{C\text{-}F}$ = 7 Hz); 129.5, 129.6, 129.8,

131.3, 132.9 (d, $^{4}J_{C\text{-}F}$ = 3 Hz); 133.3 (d, $^{4}J_{C\text{-}F}$ = 3 Hz); 156.8, 162.2 (d, $^{1}J_{C\text{-}F}$ = 246 Hz), 162.4 (d, $^{1}J_{C\text{-}F}$ = 246 Hz).

^{19}F NMR (471 MHz, $CDCl_3$): δ = -114.33 (Ar-F), -114.93 (Ar-F).

HRMS calcd. for $C_{23}H_2F_2NO^+$ ($[M+H]^+$): 366.1664, found: 366.1669.

(*E*)-1,1,1,3,3,3-Hexafluoropropan-2-yl 3-((3a*R,4*S**,6*R**,6a*R**)-3-ethyl-4-vinyl-4,5,6,6a-tetrahydro-3a*H*-cyclopenta[*d*]isoxazol-6-yl)acrylate,** (±)-**14a**

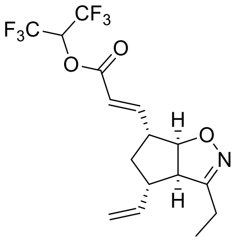

Best catalyst: G-2. Pale yellow oil; yield: 16% (32 mg); R_f = 0.55 (*n*-hexane/EtOAc 3:1).

^{1}H NMR (500 MHz, $CDCl_3$): δ = 1.15-1.20 (t, 3H, J=7 Hz, CH_2**CH_3**); 1.48-1.57 (m, 1H, CH_2); 2.02-2.10 (m, 1H, CH_2); 2.19-2.29 (m, 1H, **CH_2**CH_3); 2.44-2.54 (m, 1H, **CH_2**CH_3); 2.65-2.75 (m, 1H, H-4); 2.79-2.89 (m, 1H, H-6); 3.43-3.50 (m, 1H, H-3a); 4.79-4.86 (m, 1H, H-6a); 5.07-5.20 (m, 2H, =CH_2); 5.76-5.86 (m, 2H, **CH**$(CF_3)_2$ and =CH); 6.01-6.07 (m, 1H, =CH); 7.19-7.24 (m, 1H, =CH).

^{13}C NMR (125 MHz, $CDCl_3$): δ = 10.6, 19.7, 37.9, 48.4, 50.9, 59.8, 66.5 (septett, J = 34 Hz, **C**H$(CF_3)_2$), 88.8, 116.0, 118.9, 120.49 (q, J = 282 Hz, CF_3), 139.2, 153.6, 161.4, 162.5.

^{19}F NMR (471 MHz, $CDCl_3$): δ = -73.2 (CF_3); -73.3 (CF_3).

HRMS calcd. for $C_{16}H_{18}F_6NO_3^+$ ($[M+H]^+$): 386.1185, found: 386.1190.

(*E*)-1,1,1,3,3,3-Hexafluoropropan-2-yl 3-((3a*R,4*S**,6*R**,6a*R**)-3-ethyl-4-vinyl-4,5,6,6a-tetrahydro-3a*H*-cyclopenta[*d*]isoxazol-6-yl)acrylate** and **(*E*)-1,1,1,3,3,3-hexafluoropropan-2-yl 3-((3a*R**,4*S**,6*R**,6a*R**)-3-ethyl-6-vinyl-4,5,6,6a-tetrahydro-3a*H*-cyclopenta[*d*]isoxazol-4-yl)acrylate,** (±)-**14a** and (±)-**14b**

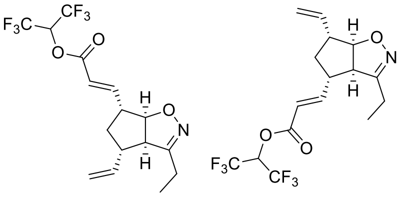

Used catalyst: G-3. Yield of (±)-**14a** and (±)-**14b**: 13% (26 mg); R_f = 0.55 (*n*-hexane/EtOAc 3:1); ratio of (±)-**14a** and (±)-**14b**: 2:1.

HRMS calcd. for $C_{16}H_{18}F_6NO_3^+$ ($[M+H]^+$): 386.1185, found: 386.1190.

(2*E*,2'*E*)-bis(1,1,1,3,3,3-Hexafluoropropan-2-yl) 3,3'-((3a*R,4*S**,6*R**,6a*R**)-3-ethyl-4,5,6,6a-tetrahydro-3a*H*-cyclopenta[*d*]isoxazole-4,6-diyl)diacrylate,** (±)-**14c**

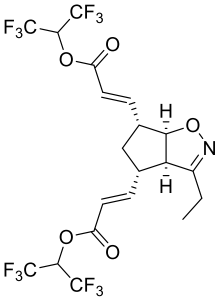

Best catalyst: HG-2. Pale yellow oil; yield: 27% (81 mg); R_f = 0.54 (*n*-hexane/EtOAc 3:1).

^{1}H NMR (500 MHz, $CDCl_3$): δ = 1.17-1.22 (m, 3H, CH_2**CH_3**); 1.61-.170 (m, 1H, CH_2); 2.13-2.29 (m, 2H, CH_2 and **CH_2**CH_3); 2.42-2.52 (m, 1H, **CH_2**CH_3); 2.86-2.95 (m, 2H, H-4 and H-6); 2.56-2.63 (m, 1H, H-3a); 4.86-4.92 (m, 1H, H-6a); 5.76-5.86 (m, 2H, **CH**$(CF_3)_2$); 6.03-6.09 (m, 2H, =CH); 7.10-7.24 (m, 2H, =CH).

^{13}C NMR (125 MHz, $CDCl_3$): δ = 10.5, 19.8, 37.1, 46.6, 50.7, 59.5, 66.6 (septett, J = 35 Hz, **C**H(CF_3)$_2$); 66.7 (septett, J = 35 Hz, **C**H(CF_3)$_2$); 88.6, 119.0, 119.6, 120.5 (q, J = 286 Hz, CF_3); 151.7, 153.1, 160.1, 162.2, 162.3.

^{19}F NMR (471 MHz, $CDCl_3$): δ = -73.3 (CF_3); -73.2 (CF_3).

HRMS calcd. for $C_{20}H_{18}F_{12}NO_5^+$ ($[M+H]^+$): 580.0987, found: 580.0988.

(*E*)-2,2,3,3,4,4,4-Heptafluorobutyl 3-((3a*R,4*S**,6*R**,6a*R**)-3-ethyl-4-vinyl-4,5,6,6a-tetrahydro-3a*H*-cyclopenta[*d*]isoxazol-6-yl)acrylate and (*E*)-2,2,3,3,4,4,4-heptafluorobutyl 3-((3a*R**,4*S**,6*R**,6a*R**)-3-ethyl-6-vinyl-4,5,6,6a-tetrahydro-3a*H*-cyclopenta[*d*]isoxazol-4-yl)acrylate,** (±)-**15a** and (±)-**15b**

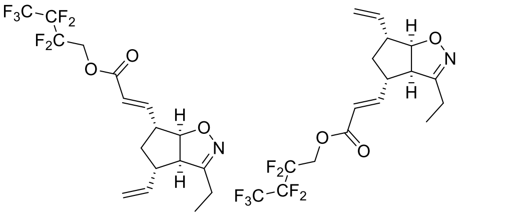

major **minor**

Best catalyst: G-2. Yield of (±)-**15a** and (±)-**15b**: 34% (73 mg); R_f=0.54 (*n*-hexane/EtOAc, 3.1); ratio of (±)-**15a** and (±)-**15b**: 3.3:1.

HRMS calcd. for $C_{17}H_{18}F_7NO_3^+$ ($[M+H]^+$): 418.1247, found: 418.1249.

(2*E*,2'*E*)-bis(2,2,3,3,4,4,4-Heptafluorobutyl) 3,3'-((3a*R,4*S**,6*R**,6a*R**)-3-ethyl-4,5,6,6a-tetrahydro-3a*H*-cyclopenta[*d*]isoxazole-4,6-diyl)diacrylate,** (±)-**15c**

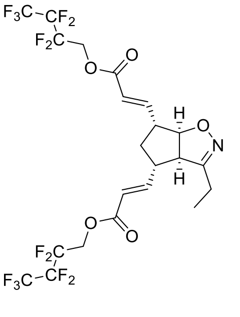

Best catalyst: HG-2. Yellow oil; yield: 42% (140 mg); R_f = 0.52 (*n*-hexane/EtOAc 3:1).

^{1}H NMR (500 MHz, $CDCl_3$): δ = 1.15-1.20 (t, 3H, J = 7 Hz, CH_3); 1.57-1.65 (m, 1H, CH_2); 2.09-2.16 (m, 1H, CH_2); 2.18-2.27 (m, 1H, CH_2); 2.41-2.54 (m, 1H, CH_2); 2.82-2.91 (m, 2H, H-4 and H-6); 3.53-3.59 (m, 1H, H-3a); 4.60-4.70 (m, 4H, $CH_2(CF_2)_2CF_3$); 4.84-4.89 (m, 1H, H-6a); 5.96-6.03 (m, 2H, =CH); 6.98-7.05 (m, 1H, =CH); 7.05-7.12 (m, 1H, =CH).

^{13}C NMR (125 MHz, $CDCl_3$): δ = 10.4, 19.7, 37.3, 46.6, 50.5, 59.4 (t, J = 27 Hz, **C**$H_2(CF_2)_2CF_3$); 59.5 (t, J = 27 Hz, **C**$H_2(CF_2)_2CF_3$); 59.6, 88.8, 100-120 (m, $CF_2CF_2CF_3$); 120.3, 120.8, 149.5, 150.9, 160.2, 163.9, 164.1.

^{19}F NMR (471 MHz, $CDCl_3$): δ= -80.86 (t, J = 9 Hz, CF_3); -80.87 (t, J = 9 Hz, CF_3); -120.4 (m, $\mathbf{CF_2}CF_2CF_3$); -127.6 (s, $CF_2\mathbf{CF_2}CF_3$).

HRMS calcd. for $C_{22}H_{20}F_{14}NO_5^+$ ($[M+H]^+$): 644.1112, found: 644.1117.

(*E*)-2,2,2-Trifluoroethyl 3-((3a*R,4*S**,6*R**,6a*R**)-3-ethyl-4-vinyl-4,5,6,6a-tetrahydro-3a*H*-cyclopenta[*d*]isoxazol-6-yl)acrylate,** (±)-**16a**

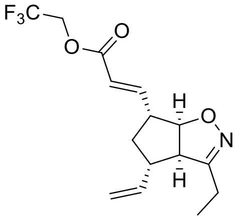

Used catalyst: HG-2. Yellow oil; yield: 7% (12 mg); R_f = 0.54 (*n*-hexane/EtOAc, 3:1).

^{1}H NMR (500 MHz, $CDCl_3$): δ = 1.15-1.19 (t, 3H, J = 7 Hz, CH_3); 1.46-1.54 (m, 1H, CH_2); 2.00-2.06 (m, 1H, CH_2); 2.18-2.30 (m, 1H, CH_2); 2.42-2.53 (m, 1H, CH_2); 2.63-2.72 (m, 1H, CH_2); 2.76-2.84 (m, 1H, CH_2); 2.43-3.49 (m, 1H, H-4); 4.48-4.57 (m, 2H, CH_2CF_3); 5.07-5.20 (m, 2H, =CH_2); 5.76-5.85 (m, 1H, =CH); 5.97-5.02 (m, 1H, =CH); 7.04-7.15 (m, 1H, =CH).

^{13}C NMR (125 MHz, $CDCl_3$): δ = 10.6, 19.7, 37.9, 48.5, 50.7, 59.8, 60.3 (q, J = 37 Hz, **C**H_2CF_3); 88.9, 115.9, 120.3, 123.0 (q, J = 277 Hz, CF_3); 139.4, 150.6, 161.4, 164.3.

^{19}F NMR (471 MHz, $CDCl_3$): δ = -73.7 (CF_3).

HRMS calcd. for $C_{15}H_{19}F_3NO_3^+$ ($[M+H]^+$): 318.1311, found: 318.1311.

(*E*)-2,2,2-Trifluoroethyl 3-((3a*R,4*S**,6*R**,6a*R**)-3-ethyl-4-vinyl-4,5,6,6a-tetrahydro-3a*H*-cyclopenta[*d*]isoxazol-6-yl)acrylate** and **(*E*)-2,2,2-trifluoroethyl 3-((3a*R**,4*S**,6*R**,6a*R**)-3-ethyl-6-vinyl-4,5,6,6a-tetrahydro-3a*H*-cyclopenta[*d*]isoxazol-4-yl)acrylate,** (±)-**16a** and (±)-**16b**

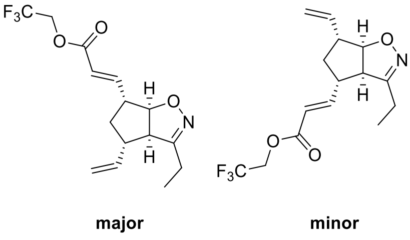

Best catalyst: G-2. Yield of (±)-**16a** and (±)-**16b**: 34% (56 mg); R_f = 0.54 (*n*-hexane/EtOAc 3:1); ratio of (±)-**16a** and (±)-**16b**: 2.5:1.

HRMS calcd. for $C_{16}H_{18}F_6NO_3^+$ ($[M+H]^+$): 318.1311, found: 318.1311.

(2*E*,2'*E*)-bis(2,2,2-Trifluoroethyl) 3,3'-((3a*R,4*S**,6*R**,6a*R**)-3-ethyl-4,5,6,6a-tetrahydro-3a*H*-cyclopenta[*d*]isoxazole-4,6-diyl)diacrylate,** (±)-**16c**

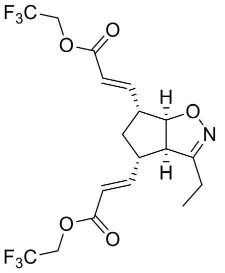

Best catalyst: HG-2. Yellow oil; yield: 52% (120 mg); R_f = 0.36 (*n*-hexane/EtOAc 3:1).

^{1}H NMR (500 MHz, $CDCl_3$): δ = 1.16-1.21 (t, 3H, *J* = 7 Hz, CH_3); 1.57-1.66 (m, 1H, CH_2); 2.09-2.17 (m, 1H, CH_2); 2.18-2.27 (m, 1H, CH_2); 2.42-2.51 (m, 1H, CH_2); 2.82-2.90 (m, 2H, H-4 and H-6); 3.54-3.60 (m, 1H, H-3a); 4.49-4.57 (m, 4H, CH_2CF_3); 4.84-4.89 (m, 1H, H-6a); 5.98-6.04 (m, 2H, =CH); 6.99-7.06 (m, 1H, =CH); 7.06-7.13 (m, 1H, =CH).

^{13}C NMR (125 MHz, $CDCl_3$): δ = 10.5, 19.8, 37.3, 46.6, 50.5, 59.6, 60.4 (q, *J* = 37 Hz, **C**H_2CF_3); 60.5 (q, *J* = 37 Hz, **C**H_2CF_3); 88.8, 120.4, 120.9, 122.9 (q, *J* = 277 Hz, CF_3); 123.0 (q, *J* = 277 Hz, CF_3); 149.5, 150.9, 160.3, 163.9, 164.1.

^{19}F NMR (471 MHz, $CDCl_3$): δ = -73.8 (CF_3); -73.7 (CF_3).

HRMS calcd. for $C_{18}H_{20}F_6NO_5^+$ ($[M+H]^+$): 444.1240, found: 444.1244.

(*E*)-5-((3a*R,4*S**,6*R**,6a*R**)-3-Ethyl-4-vinyl-4,5,6,6a-tetrahydro-3a*H*-cyclopenta[*d*]isoxazol-6-yl)-1,1,1-trifluoro-2-(trifluoromethyl)pent-4-en-2-ol,** (±)-**17a**

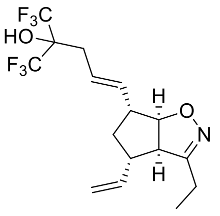

Best catalyst: G-2. Yellow oil; yield: 34% (20 mg); R_f = 0.34 (*n*-hexane/EtOAc 4:1).

^1H NMR (500 MHz, $CDCl_3$): δ = 1.14-1.19 (t, 3H, *J* =7 Hz, CH_3); 1.36-1.45 (m, 1H, CH_2); 1.92-1.98 (m, 1H, CH_2); 2.20-2.29 (m, 1H, CH_2); 2.46-2.56 (m, 1H, CH_2); 2.64-2.86 (m, 4H, H-4, H-6 and $CH_2C(CF_3)_2OH$); 3.41-3.48 (m, 1H, H-3a), 4.62-4.68 (m, 1H, H-6a); 4.72 (brs, 1H, OH); 5.06-5.19 (m, 2H, $=CH_2$); 5.57-5.65 (m, 1H, =CH); 5.69-5.75 (m, 1H, =CH); 5.77-5.87 (m, 1H, =CH).

^{13}C NMR (125 MHz, $CDCl_3$): δ = 10.5, 19.5, 28.7, 38.7, 45.8, 48.0, 59.3, 75.7 (septet, $^2J_{C-F}$ = 29 Hz, **C**(CF3)2OH); 89.6, 115.5, 121.7, 123.0 (q, $^1J_{C-F}$ = 287 Hz, CF_3); 123.2 (q, $^1J_{C-F}$ = 287 Hz, CF_3);126.6, 136.2, 139.6, 162.2.

^{19}F NMR (471 MHz, $CDCl_3$): δ = -78.0 (CF_3); -75.3 (CF_3).

HRMS calcd. for $C_{16}H_{20}F_6NO_2^+$ ($[M+H]^+$): 372.1392, found: 372.1397.

(*E*)-5-((3a*R,4*S**,6*R**,6a*R**)-3-Ethyl-6-vinyl-4,5,6,6a-tetrahydro-3a*H*-cyclopenta[*d*]isoxazol-4-yl)-1,1,1-trifluoro-2-(trifluoromethyl)pent-4-en-2-ol,** (±)-**17b**

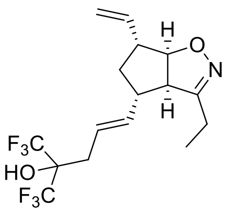

Best catalyst: G-2. Yellow oil; yield: 11% (65 mg); R_f = 0.32 (*n*-hexane/EtOAc, 4:1).

^1H NMR (500 MHz, $CDCl_3$): δ = 1.12-1.19 (m, 3H, CH_3); 1.36-1.45 (m, 1H, CH_2); 1.93-2.00 (m, 1H, CH_2); 2.17-2.29 (m, 1H, CH_2); 2.42-2.53 (m, 1H, CH_2); 2.57-2.74 (m, 4H, H-4, H-6 and $CH_2(CF_3)_2OH$); 3.36-3.47 (m, 1H, H-3a); 3.76 (brs, 1H, OH); 4.63-4.76 (m, 1H, H-6a); 5.03-5.19 (m, 2H, $=CH_2$); 5.52-5.64 (m, 1H, =CH); 5.73-5.86 (m, 2H, =CH).

^{13}C NMR (125 MHz, $CDCl_3$): δ = 10.5, 19.5, 33.8, 38.4, 48.2, 50.6, 59.6, 75.5 (septet, $^2J_{C-F}$ = 29 Hz, **C**$H_2(CF_3)_2OH$); 89.7, 115.4, 120.9, 123.0 (q, $^1J_{C-F}$ = 286 Hz, CF_3); 138.1, 139.8, 161.7.

^{19}F NMR (471 MHz, $CDCl_3$): δ = -76.5 (CF_3); -76.3 (CF_3).

HRMS calcd. for $C_{16}H_{20}F_6NO_2^+$ ($[M+H]^+$): 372.1392, found: 372.1398.

(3a*R,4*S**,6*R**,6a*R**)-3-Ethyl-4-((*E*)-4-fluorostyryl)-6-vinyl-4,5,6,6a-tetrahydro-3a*H*-cyclopenta[*d*]isoxazole** and **(3a*R**,4*S**,6*R**,6a*R**)-3-ethyl-6-((*E*)-4-fluorostyryl)-4-vinyl-4,5,6,6a-tetrahydro-3a*H*-cyclopenta[*d*]isoxazole,** (±)-**19a** and (±)-**19b**

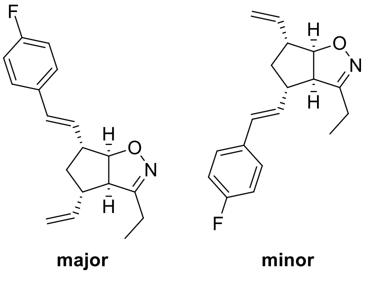

Best catalyst: G-2. Yield of (±)-**19a** and (±)-**19b**: 37% (55 mg); R_f = 0.53 (*n*-hexane/EtOAc 3:1); ratio of (±)-**19a** and (±)-**19b**: 2:1.

HRMS calcd. for $C_{18}H_{20}F_6NO_5^+$ ($[M+H]^+$): 286.1601, found: 286.1603.

(3a*R,4*S**,6*R**,6a*R**)-3-Ethyl-4,6-bis((*E*)-4-fluorostyryl)-4,5,6,6a-tetrahydro-3a*H*-cyclopenta[*d*]isoxazole,** (±)-**19c**

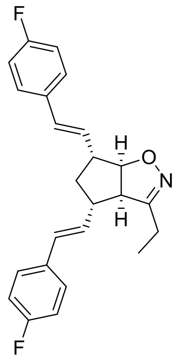

Best catalyst: HG-2. White solid; yield: 38% (76 mg); R_f = 0.45 (*n*-hexane/EtOAc 3:1), mp. 96-97 °C.

^{1}H NMR (500 MHz, $CDCl_3$): δ = 1.16-1.20 (t, 3H, J=7 Hz, CH_3); 1.55-1.64 (m, 1H, CH_2); 2.06-2.13 (m, 1H, CH_2); 2.23-2.32 (m, 1H, CH_2); 2.48-2.57 (m, 1H, CH_2); 2.77-2.86 (m, 2H, H-4 and H-6); 3.48-3.54 (m, 1H, H-3a); 4.82-4.86 (m, 1H, H-6a); 6.07-6.13 (m, 1H, =CH); 6.16-6.22 (m, 1H, =CH); 6.44-6.47 (m, 1H, =CH); 6.47-6.50 (m, 1H, =CH).

^{13}C NMR (125 MHz, $CDCl_3$): δ = 10.7,19.9, 39.3, 47.9, 51.3, 60.2, 90.2, 115.4 (d, $^2J_{C-F}$ = 20 Hz); 115.6 (d, $^2J_{C-F}$ = 20 Hz); 127.6 (d, $^3J_{C-F}$ = 5 Hz); 127.6 (d, $^3J_{C-F}$ = 5 Hz); 129.5, 129.6, 129.8, 131.4, 132.9 (d, $^4J_{C-F}$ = 3 Hz); 133.3 (d, $^4J_{C-F}$ = 3 Hz); 162.2 (d, $^1J_{C-F}$ = 246 Hz); 162.3 (d, $^1J_{C-F}$ = 246 Hz).

^{19}F NMR (471 MHz, $CDCl_3$): δ = -114.9 (Ar-F), -114.3 (Ar-F).

HRMS calcd. for $C_{24}H_{24}F_2NO^+$ ($[M+H]^+$): 380.1820, found: 380.1824.

(*E*)-1,1,1,3,3,3-Hexafluoropropan-2-yl 3-((3a*R,4*S**,6*R**,6a*R**)-3-phenyl-4-vinyl-4,5,6,6a-tetrahydro-3a*H*-cyclopenta[*d*]isoxazol-6-yl)acrylate,** (±)-**20a**

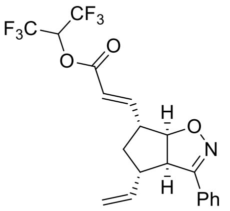

Best catalyst: G-3. Colorless oil; yield: 30% (55 mg); R_f = 0.42 (*n*-hexane/EtOAc 7:1).

^{1}H NMR (500 MHz, $CDCl_3$): δ = 1.58-1.66 (m, 1H, CH_2); 2.09-2.17 (m, 1H, CH_2); 2.79-2.87 (m, 1H, H-4); 2.93-3.01 (m, 1H, H-6); 3.97-4.03 (m, 1H, H-3a); 5.04-5.11 (m, 2H, H-6a and =CH_2); 5.14-5.18 (m, 1H, =CH_2); 5.79-5.95 (m, 2H, $CH(CF_3)_2$ and =CH); 6.06-6.12 (m, 1H, =CH); 7.23-7.30 (m, 1H, =CH); 7.35-7.43 (m, 3H, Ar-H); 7.66-7.70 (m, 2H, Ar-H).

^{13}C NMR (125 MHz, $CDCl_3$): δ = 37.9, 48.3, 50.3, 57.9, 66.5 (septett, $^2J_{C\text{-}F}$ = 35 Hz, $CH(CF_3)_2$); 90.4, 116.7, 119.1, 120.5 (q, $^1J_{C\text{-}F}$ = 281 Hz, CF_3); 127.7, 128.4, 128.6, 130.2, 139.9, 152.9, 159.0, 162.5.

^{19}F NMR (471 MHz, $CDCl_3$): δ = -73.2 (CF_3).

HRMS calcd. for $C_{20}H_{18}F_6NO_3^+$ ($[M+H]^+$): 434.1185, found: 434.1189.

(2*E*,2'*E*)-Bis(1,1,1,3,3,3-hexafluoropropan-2-yl) 3,3'-((3a*R,4*S**,6*R**,6a*R**)-3-phenyl-4,5,6,6a-tetrahydro-3a*H*-cyclopenta[*d*]isoxazole-4,6-diyl)diacrylate,** (±)-**20c**

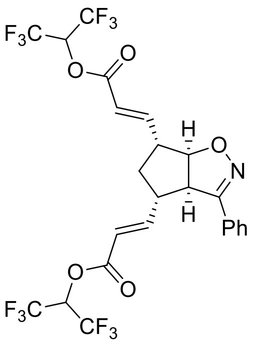

Best catalyst: HG-2. White solid; yield: 38% (100 mg); R_f = 0.39 (*n*-hexane/EtOAc 7:1), mp. 112-113 °C.

^{1}H NMR (500 MHz, $CDCl_3$): δ = 1.71-1.80 (m, 1H, CH_2); 2.18-2.25 (m, 1H, CH_2); 2.94-3.03 (m, 1H, H-4); 3.03-3.11 (m, 1H, H-6); 4.09-4.15 (m, 1H, H-3a); 5.10-5.16 (m, 1H, H-6a); 5.78-5.94 (m, 3H, $CH(CF_3)_2$ and =CH); 6.09-6.15 (m, 1H, =CH); 7.17-7.29 (m, 2H, =CH); 7.34-7.47 (m, 3H, Ar-H); 7.54-7.60 (m, 2H, Ar-H).

^{13}C NMR (125 MHz, $CDCl_3$): δ = 37.3, 47.3, 50.3, 57.8, 66.6 (septett, $^{2}J_{C-F}$ = 35 Hz, **C**H(CF_3)$_2$); 66.7 (septett, $^{2}J_{C-F}$ = 35 Hz, **C**H(CF_3)$_2$); 90.2, 119.8, 120.3 (q, $^{1}J_{C-F}$ = 282 Hz, CF_3); 127.5, 127.7, 128.8, 130.7, 151.6, 153.3, 158.1, 162.1, 162.2.

^{19}F NMR (471 MHz, $CDCl_3$): δ = -73.2 (CF_3); -73.3 (CF_3).

HRMS calcd. for $C_{24}H_{18}F_{12}NO_5^+$ ($[M+H]^+$): 628.0987, found: 628.0987.

(*E*)-2,2,3,3,4,4,4-Heptafluorobutyl 3-((3a*R,4*S**,6*R**,6a*R**)-3-phenyl-4-vinyl-4,5,6,6a-tetrahydro-3a*H*-cyclopenta[*d*]isoxazol-6-yl)acrylate,** (±)-**21a**

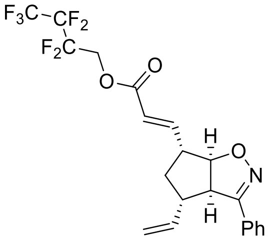

Best catalyst: G-2. Yellow oil; yield: 27% (53 mg); R_f = 0.38 (*n*-hexane/EtOAc 7:1).

^{1}H NMR (500 MHz, $CDCl_3$): δ = 1.55-1.65 (m, 1H, CH_2); 2.07-2.15 (m, 1H, CH_2); 2.77-2.86 (m, 1H, H-4); 2.90-2.99 (m, 1H, H-6); 3.95-4.02 (m, 1H, H-3a); 4.60-4.71 (m, 2H, $CH_2(CF_2)_2CF_3$); 5.01-5.17 (m, 3H, H-6a and =CH_2); 5.83-.596 (m, 1H, =CH); 5.99-6.06 (m, 1H, CH); 7.10-7.18 (m, 1H, =CH); 7.34-7.43 (m, 3H, Ar-H); 7.64-7.71 (m, 2H, Ar-H).

^{13}C NMR (125 MHz, $CDCl_3$): δ = 37.9, 48.3, 50.1, 57.8, 59.3 (t, $^{2}J_{C-F}$ = 27 Hz, **C**H$_2$(CF_2)$_2CF_3$); 90.6, 116.5, 100-120 (m, $CF_2CF_2CF_3$); 120.4, 127.7, 12.84,.128.6, 130.1, 140.1, 150.6, 159.0, 164.3.

^{19}F NMR (471 MHz, $CDCl_3$): δ= -80.86 (t, J = 9 Hz, CF_3); -80.85 (t, J = 9 Hz, CF_3); -120.4 (m, **CF**$_2CF_2CF_3$); -127.6 (s, CF_2**CF**$_2CF_3$).

HRMS calcd. for $C_{21}H_{19}F_7NO_3^+$ ($[M+H]^+$): 466.1247, found: 466.1253.

(2*E*,2'*E*)-Bis(2,2,3,3,4,4,4-heptafluorobutyl) 3,3'-((3a*R,4*S**,6*R**,6a*R**)-3-phenyl-4,5,6,6a-tetrahydro-3a*H*-cyclopenta[*d*]isoxazole-4,6-diyl)diacrylate,** (±)-**21c**

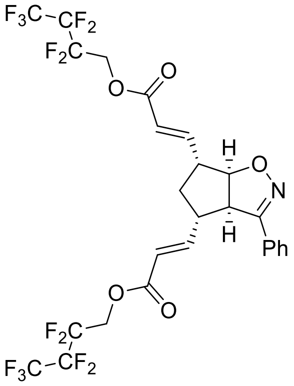

Best catalyst: HG-2. Yellow solid; yield: 37% (108 mg); R_f = 0.31 (*n*-hexane/EtOAc 7:1), mp. 69-70 °C.

^{1}H NMR (500 MHz, CDCl$_3$): δ = 1.65-1.75 (m, 1H, CH$_2$); 2.14-2.21 (m, 1H, CH$_2$); 2.92-3.07 (m, 2H, H-4 and H-6); 4.05-4.11 (m, 1H, H-3a); 4.54-4.74 (m, 4H, CH$_2$(CF$_2$)CF$_3$); 5.07-5.13 (m, 1H, H-6a); 5.82-5.87 (m, 1H, =CH); 6.02-6.07 (m, 1H, =CH); 7.06-7.15 (m, 2H, =CH); 7.35-7.44 (m, 3H, Ar-H); 7.54-7.59 (m, 2H, Ar-H).

^{13}C NMR (125 MHz, CDCl$_3$): δ = 37.4, 46.9, 50.1, 59.4 (t, $^2J_{C-F}$ = 27 Hz, **C**H$_2$(CF$_2$)$_2$CF$_3$); 90.4, 100-120 (m, CF$_2$CF$_2$CF$_3$); 120.9, 121.0,127.4, 127.9, 128.8, 130.5, 149.4, 151.3, 158.2, 164.0.

^{19}F NMR (471 MHz, CDCl$_3$): δ= -80.82 (t, J = 9 Hz, CF$_3$); -80.84 (t, J = 9 Hz, CF$_3$); -120.4 (m, **CF$_2$**CF$_2$CF$_3$); -127.6 (s, CF$_2$**CF$_2$**CF$_3$).

HRMS calcd. for $C_{26}H_{20}F_{14}NO_5^+$ ($[M+H]^+$): 692.1112, found: 692.1119.

(*E*)-2,2,2-Trifluoroethyl 3-((3a*R,4*S**,6*R**,6a*R**)-3-phenyl-4-vinyl-4,5,6,6a-tetrahydro-3a*H*-cyclopenta[*d*]isoxazol-6-yl)acrylate,** (±)-**22a**

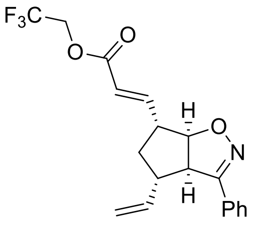

Best catalyst: G-2. Pale yellow oil; yield: 37% (57 mg); R_f = 0.33 (*n*-hexane/EtOAc 7:1).

^{1}H NMR (500 MHz, $CDCl_3$): δ = 1.59-1.66 (m, 1H, CH_2); 2.10-2.17 (m, 1H, CH_2); 2.80-2.88 (m, 1H, H-4); 2.92-3.01 (m, 1H, H-6); 3.97-4.05 (m, 1H, H-3a); 4.52-4.62 (m, 2H, CH_2CF_3); 5.03-5.21 (m, 3H, H-6a and =CH_2); 5.87-5.97 (m, 1H, =CH); 6.03-6.09 (m, 1H, =CH); 7.14-7.21 (m, 1H, CH); 7.37-7.47 (m, 3H, Ar-H); 7.68-7.73 (m, 2H, Ar-H).

^{13}C NMR (125 MHz, $CDCl_3$): δ = 37.9, 48.3, 50.1, 57.8, 60.4 (q, $^2J_{C-F}$ = 37 Hz, **C**H_2CF_3); 90.6, 116.5, 120.5, 123.0 (q, $^1J_{C-F}$ = 277 Hz, CF_3); 127.7, 128.4, 128.6, 130.1, 140.1, 150.5, 159.0, 164.3.

^{19}F NMR (471 MHz, $CDCl_3$): δ = -73.7 (CF_3).

HRMS calcd. for $C_{19}H_{19}F_3NO_3^+$ ($[M+H]^+$): 366.1311, found: 366.1316.

(2*E*,2'*E*)-Bis(2,2,2-trifluoroethyl) 3,3'-((3a*R,4*S**,6*R**,6a*R**)-3-phenyl-4,5,6,6a-tetrahydro-3a*H*-cyclopenta[*d*]isoxazole-4,6-diyl)diacrylate,** (±)-**22c**

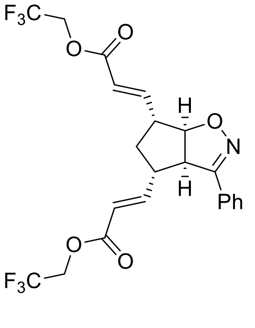

Best catalyst: HG-2. Yellow oil; yield: 48% (99 mg); R_f = 0.41 (*n*-hexane/EtOAc 5:1).

^{1}H NMR (500 MHz, $CDCl_3$): δ = 1.68-1.78 (m, 1H, CH_2); 2.16-2.23 (m, 1H, CH_2); 2.94-3.09 (m, 2H, H-4 and H-6); 4.08-4.13 (m, 1H, H-3a), 4.51-4.64 (m, 4H, CH_2CF_3); 5.09-5.15 (m, 1H,

H-6a); 5.85-5.92 (m, 1H, =CH); 6.04-6.10 (m, 1H, =CH); 7.08-7.19 (m, 2H, =CH); 7.37-7.47 (m, 3H, Ar-H); 7.57-7.62 (m, 2H, Ar-H).

^{13}C NMR (125 MHz, $CDCl_3$): δ = 37.4, 46.9, 50.1, 57.6, 59.9, 60.42 (q, $^2J_{C\text{-}F}$ = 37 Hz, **C**H_2CF_3); 60.45 (q, $^2J_{C\text{-}F}$ = 37 Hz, **C**H_2CF_3); 90.4, 120.9, 122.54 (q, $^1J_{C\text{-}F}$ = 277 Hz, CF_3); 122.59 (q, $^1J_{C\text{-}F}$ = 277 Hz, CF_3); 127.4, 127.9, 128.8, 129.0, 130.5, 149.3, 151.3, 158.2, 164.0, 164.1.

^{19}F NMR (471 MHz, $CDCl_3$): δ = -73.77 (CF_3); -73.77 (CF_3).

HRMS calcd. for $C_{22}H_{20}F_6NO_5^+$ ([M+H]$^+$): 492.1240, found: 492.1247.

(*E*)-1,1,1-Trifluoro-5-((3a*R,4*S**,6*R**,6a*R**)-3-phenyl-4-vinyl-4,5,6,6a-tetrahydro-3a*H*-cyclopenta[*d*]isoxazol-6-yl)-2-(trifluoromethyl)pent-4-en-2-ol,** (±)-**23a**

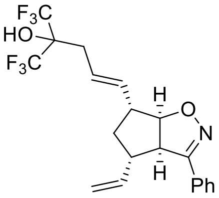

Best catalyst: G-2. Brown oil; yield: 54% (95 mg); R_f = 0.45 (*n*-hexane/EtOAc 4:1).

^{1}H NMR (500 MHz, $CDCl_3$): δ = 1.44-1.55 (m, 1H, CH_2); 1.99-2.08 (m, 1H, CH_2); 2.69-2.85 (m, 4H, H-4, H-6 and $CH_2(CF_3)_2OH$); 3.47 (brs, 1H, OH); 3.88-3.97 (m, 1H, H-3a); 4.91-4.98 (m, 1H, H-6a); 5.01-5.17 (m, 2H, =CH_2); 5.57-5.70 (m, 1H, =CH); 5.77-5.97 (m, 2H, =CH); 7.32-7.43 (m, 3H, Ar-H); 2.64-2.71 (m, 2H, Ar-H).

^{13}C NMR (125 MHz, $CDCl_3$): δ = 33.7, 38.4, 48.2, 50.1, 57.6, 75.2 (septet, $^2J_{C\text{-}F}$ = 29 Hz, **C**$H_2(CF_3)_2OH$); 91.4, 116.0, 121.0 122.9 (q, $^1J_{C\text{-}F}$ = 287 Hz, CF_3); 127.6, 128.5, 128.6, 130.0, 138.7, 140.6, 159.1.

^{19}F NMR (471 MHz, $CDCl_3$): δ = -76.5 (CF_3); -76.4 (CF_3).

HRMS calcd. for $C_{20}H_{20}F_6NO_2^+$ ([M+H]$^+$): 420.1393, found: 420.1392.

(3a*R,4*S**,6*R**,6a*R**)-6-((*E*)-4-Fluorostyryl)-3-phenyl-4-vinyl-4,5,6,6a-tetrahydro-3a*H*-cyclopenta[*d*]isoxazole,** (±)-**25a**

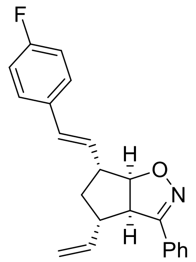

Best catalyst: G-2. White solid; yield: 28% (39 mg); R_f = 0.39 (*n*-hexane/EtOAc 7:1), mp. 57-58 °C.

^{1}H NMR (500 MHz, $CDCl_3$): δ = 1.55-1.64 (m, 1H, CH_2); 2.07-2.14 (m, 1H, CH_2); 2.74-2.85 (m, 1H, H-4); 2.85- 2.95 (m, 1H, H-6); 3.91-3.97 (m, 1H, H-3a); 5.00-5.14 (m, 3H, H-6a and =CH_2); 5.88-5.98 (m, 1H, =CH); 6.15-6.22 (m, 1H, =CH); 6.46-6.53 (m, 1H, =CH); 6.96-7.02 (m, 2H, Ar-H); 7.26-7.40 (m, 5H, Ar-H); 7.66-7.72 (m, 2H, Ar-H).

^{13}C NMR (125 MHz, $CDCl_3$): δ = 38.7, 48.4, 50.6, 57.7, 91.9, 115.4 (d, $^2J_{C\text{-}F}$ = 21 Hz); 115.8, 127.65 (d, $^3J_{C\text{-}F}$ = 7 Hz); 127.66; 128.4, 128.8, 129.6, 129.8, 133.3(d, $^4J_{C\text{-}F}$ = 3 Hz); 140.8, 159.1, 162.2 (d, $^1J_{C\text{-}F}$ = 246 Hz).

^{19}F NMR (471 MHz, $CDCl_3$): δ = -114.9 (Ar-F).

HRMS calcd. for $C_{22}H_{21}FNO^+$ ($[M+H]^+$): 334.1601, found: 334.1603.

(3a*R,4*S**,6*R**,6a*R**)-4,6-Bis((*E*)-4-fluorostyryl)-3-phenyl-4,5,6,6a-tetrahydro-3a*H*-cyclopenta[*d*]isoxazole,** (±)-**25c**

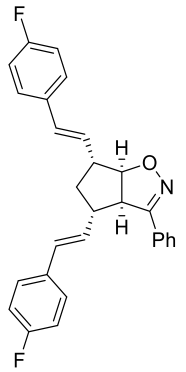

Best catalyst: G-2. White solid; yield: 49% (88 mg); R_f = 0.52 (*n*-hexane/EtOAc 5:1), mp. 163-164 °C.

^{1}H NMR (500 MHz, $CDCl_3$): δ = 1.63-1.73 (m, 1H, CH_2); 2.11-2.19 (m, 1H, CH_2); 2.86-2.99 (m, 2H, H-4 and H-6); 3.97-4.04 (m, 1H, H-3a); 5.04-5.10 (m, 1H, H-6a); 6.13-6.26 (m, 2H, =CH); 6.27-6.34 (m, 1H, =CH); 6.48-6.54 (m, 1H, =CH); 6.95-7.05 (m, 4H, Ar-H); 7.26-7.40 (m, 7H, Ar-H); 7.66-7.73 (m, 2H, Ar-H).

^{13}C NMR (125 MHz, $CDCl_3$): δ = 39.1, 48.1, 50.7, 58.3, 91.8, 115.4 (d, $^2J_{C-F}$ = 16 Hz); 115.5 (d, $^2J_{C-F}$ = 16 Hz); 127.6 (d, $^3J_{C-F}$ = 8 Hz); 127.8; 128.5, 128.8, 129.5, 129.9 (d, $^3J_{C-F}$ = 8 Hz); 130.3, 132.0, 133.0 (d, $^4J_{C-F}$ = 3 Hz); 133.3 (d, $^4J_{C-F}$ = 3 Hz); 159.0, 162.2 (d, $^1J_{C-F}$ = 247 Hz); 162.3 (d, $^1J_{C-F}$ = 247 Hz).

^{19}F NMR (471 MHz, $CDCl_3$): δ = -114.7 (Ar-F); -114.3 (Ar-F).

HRMS calcd. for $C_{28}H_{24}F_2NO^+$ ([M+H]$^+$): 428.1820, found: 428.1825.

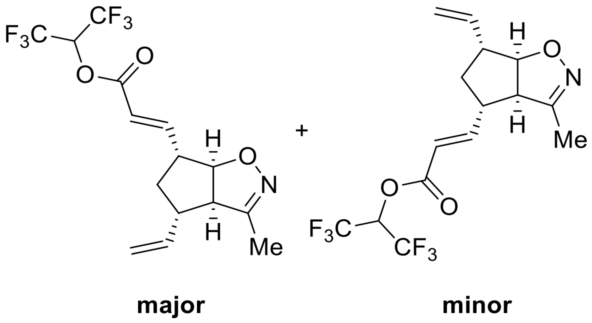

(±)-**8a** and (±)-**8b**

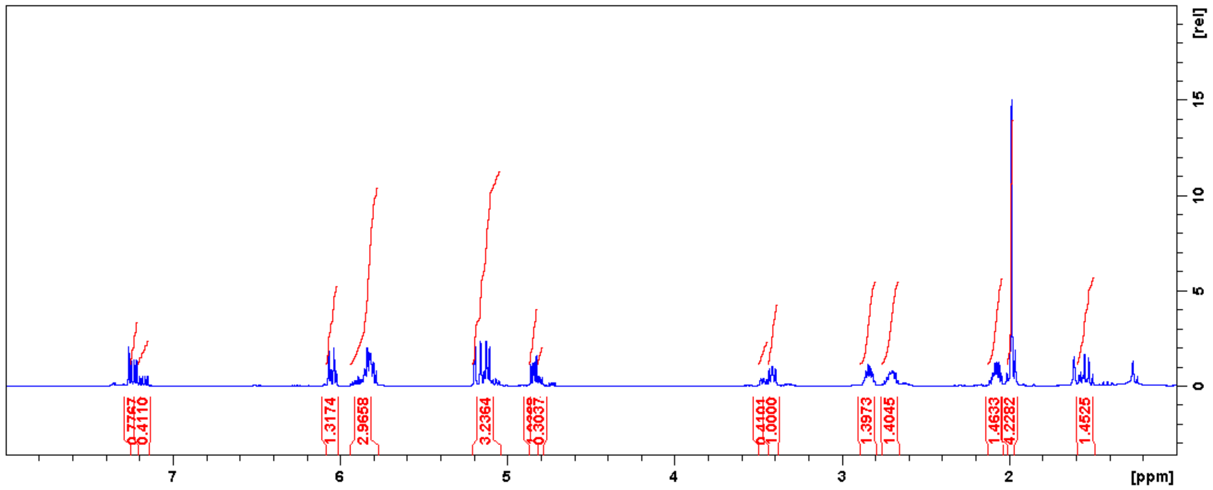

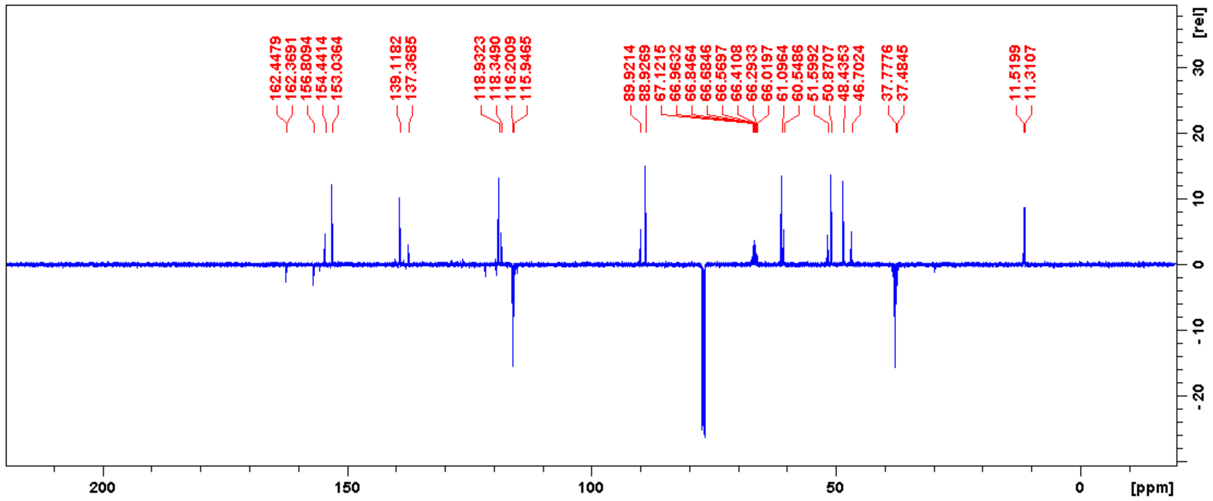

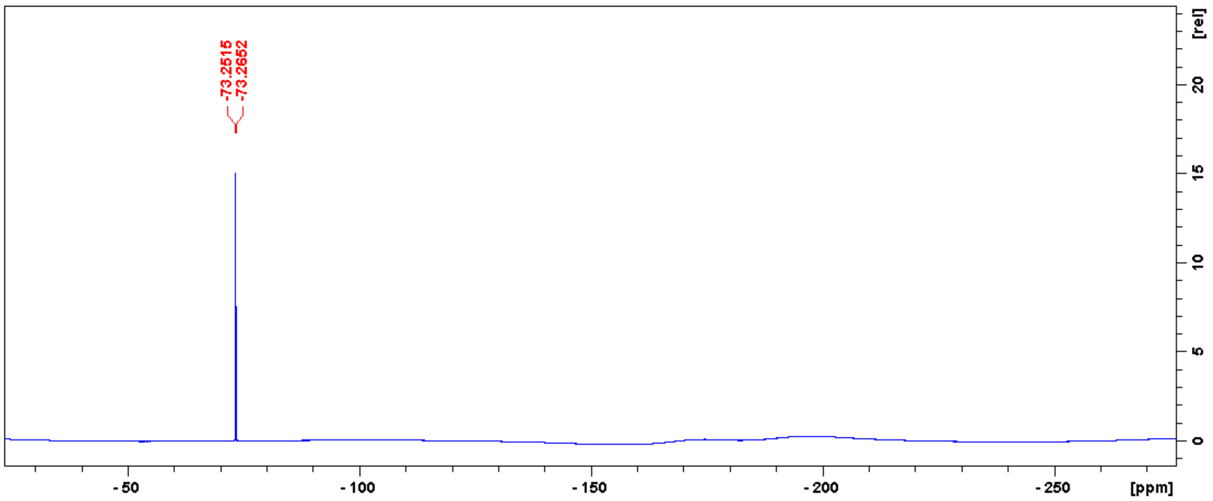

-73.2515
-73.2652
[rel]
20
15
10
5
0
-50
-100
-150
-200
-250
[ppm]

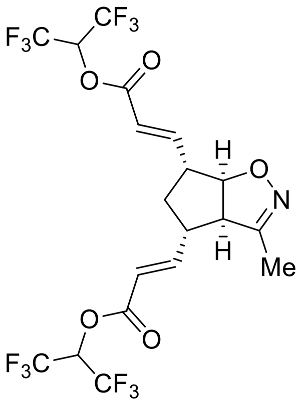

(±)-**8c**

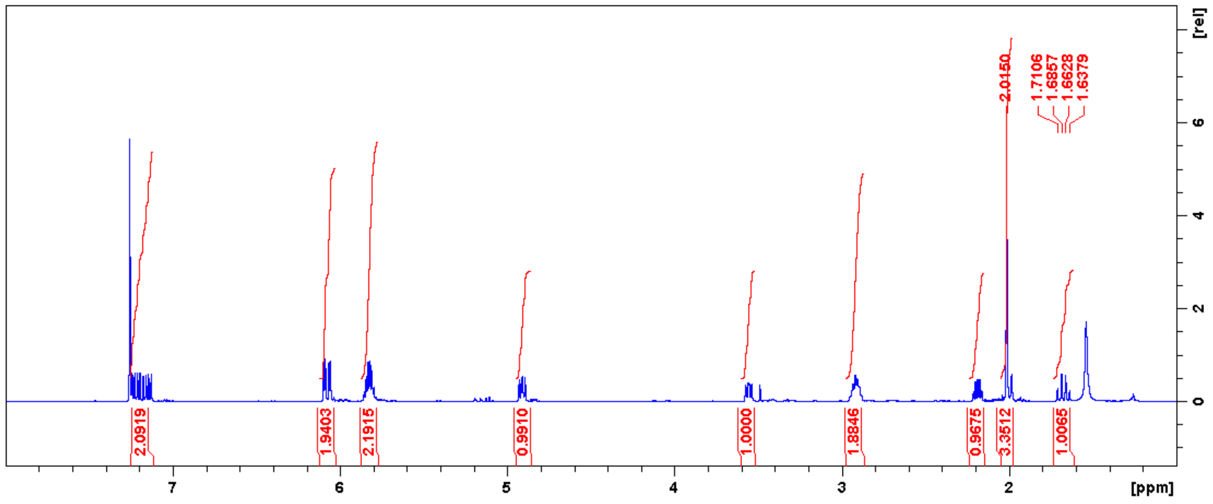

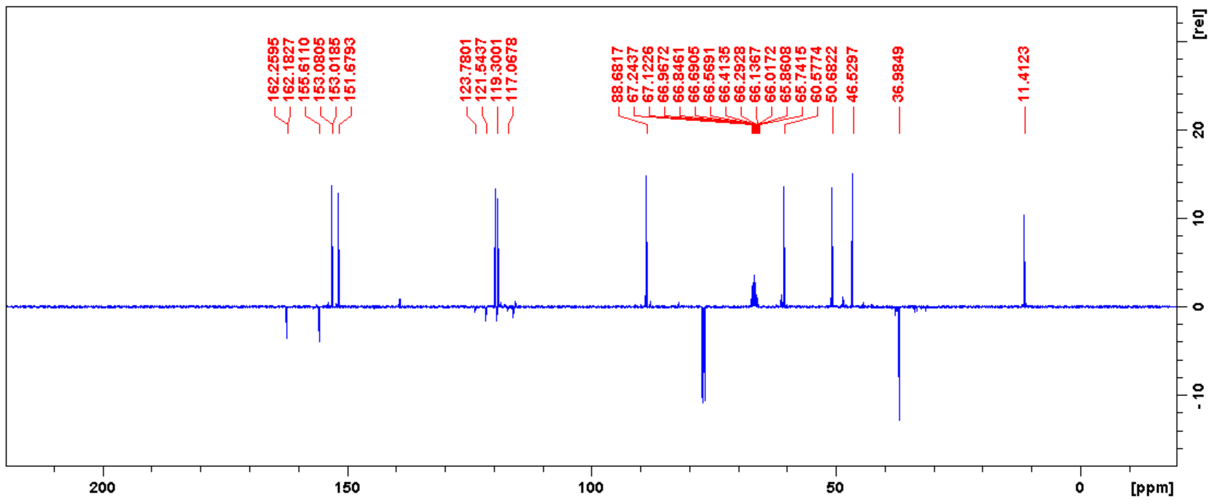

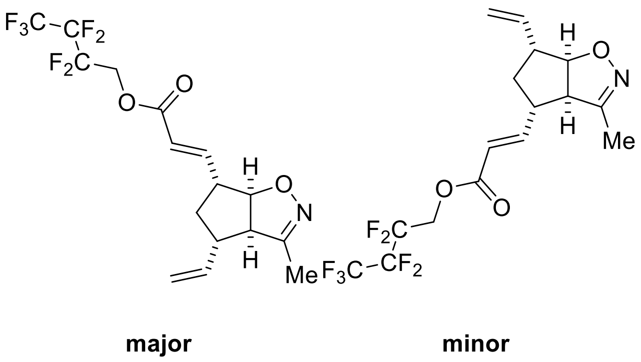
F3C-CF2
F2C
O
O
H
O
N
H
Me
major
H
O
N
H
Me
O
O
F2C
F3C-CF2
minor

(±)-**9a** and (±)-**9b**

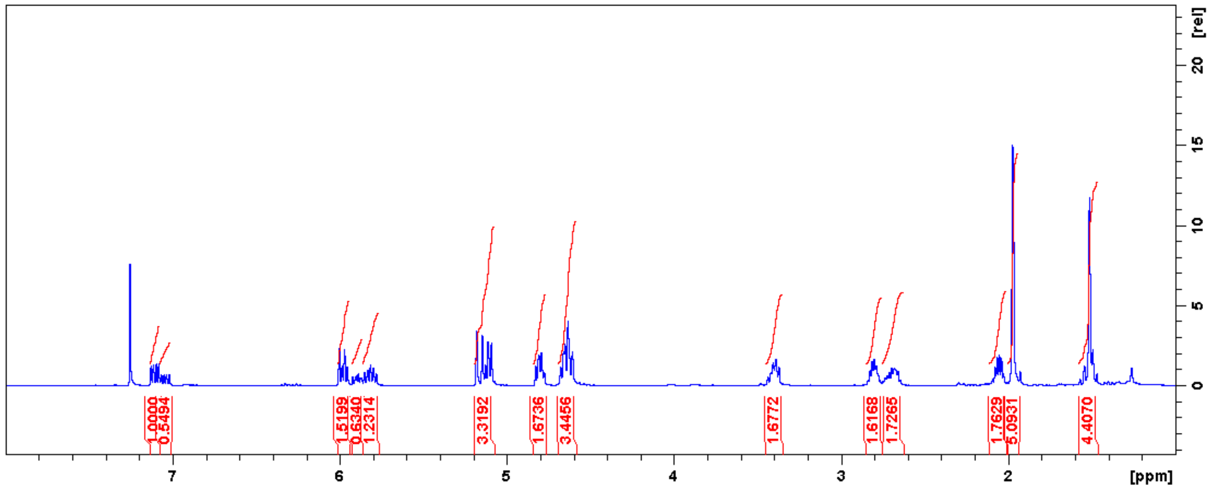
1.0000
0.5494
1.5199
0.6340
1.2314
3.3192
1.6736
3.4456
1.6772
1.6168
1.7265
1.7629
5.0931
4.4070
[ppm]
[rel]

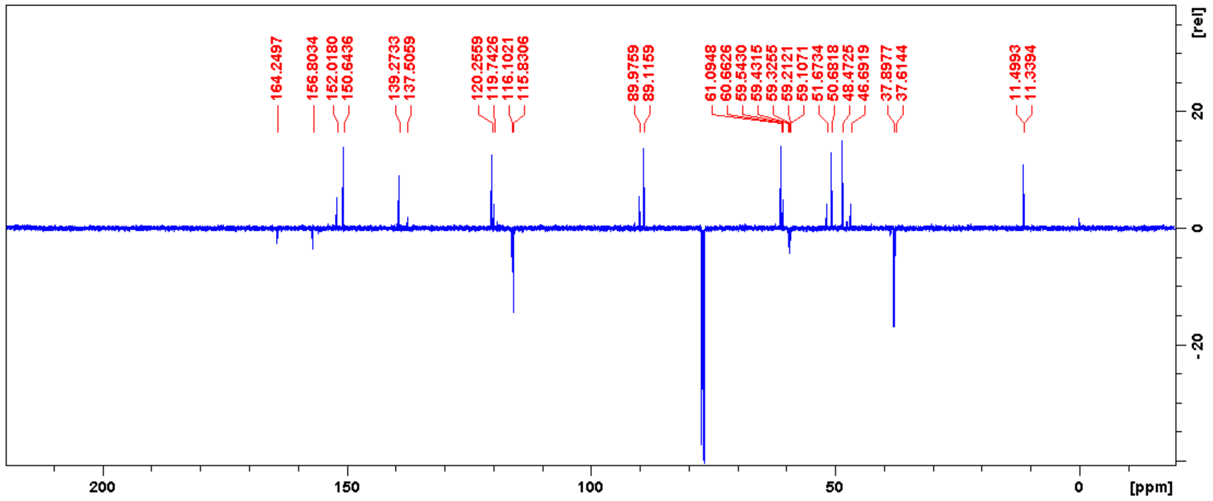
164.2497
156.8034
152.0180
150.6436
139.2733
137.5059
120.2559
119.7426
116.1021
115.8306
89.9759
89.1159
61.0948
60.6626
59.5430
59.4315
59.3255
59.2121
59.1071
51.6734
50.6818
48.4725
46.6919
37.8977
37.6144
11.4993
11.3394
[ppm]
[rel]

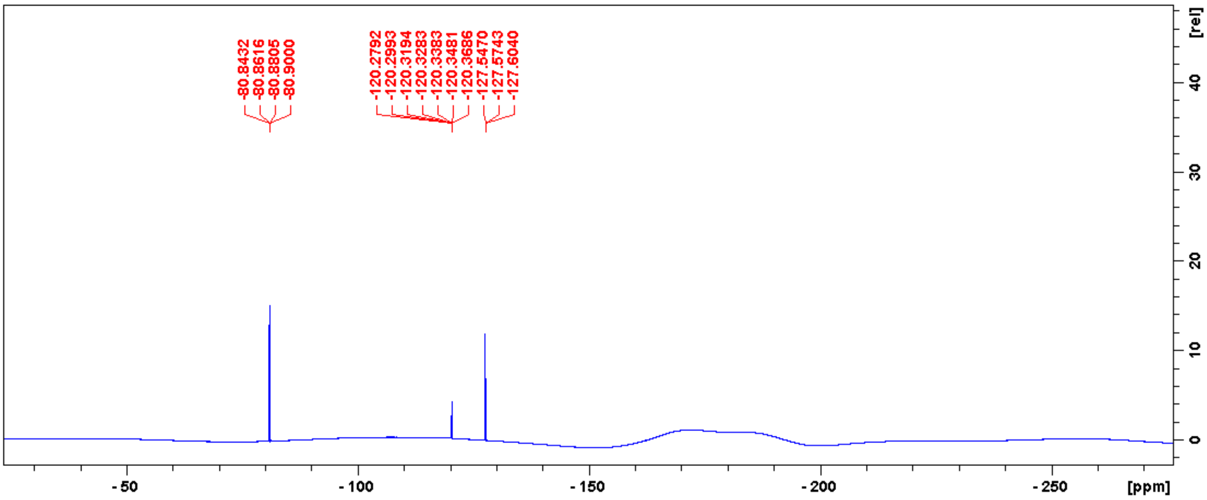

-80.8432
-80.8616
-80.8805
-80.9000
-120.2792
-120.2993
-120.3194
-120.3283
-120.3383
-120.3481
-120.3686
-127.5470
-127.5743
-127.6040
[rel]
40
30
20
10
0
- 50
- 100
- 150
- 200
- 250
[ppm]

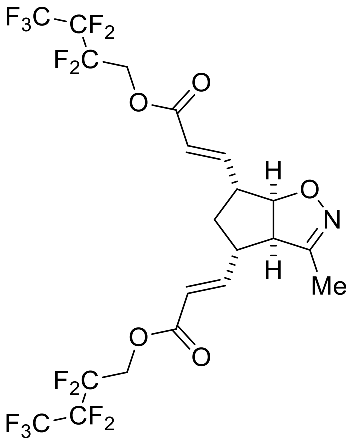

(±)-**9c**

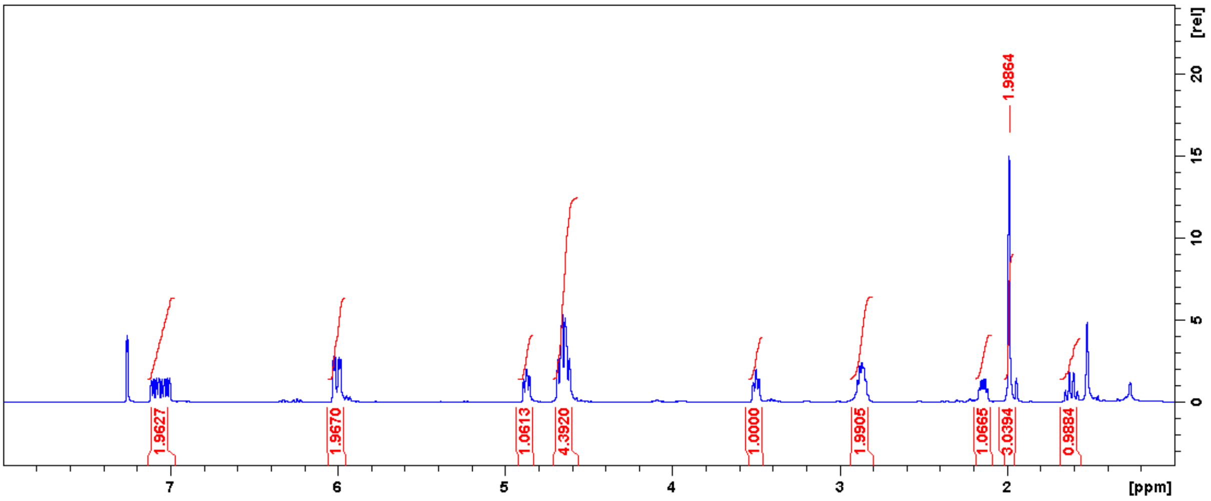

1.9864
1.9627
1.9670
1.0613
4.3920
1.0000
1.9905
1.0665
3.0394
0.9884
[rel]
[ppm]

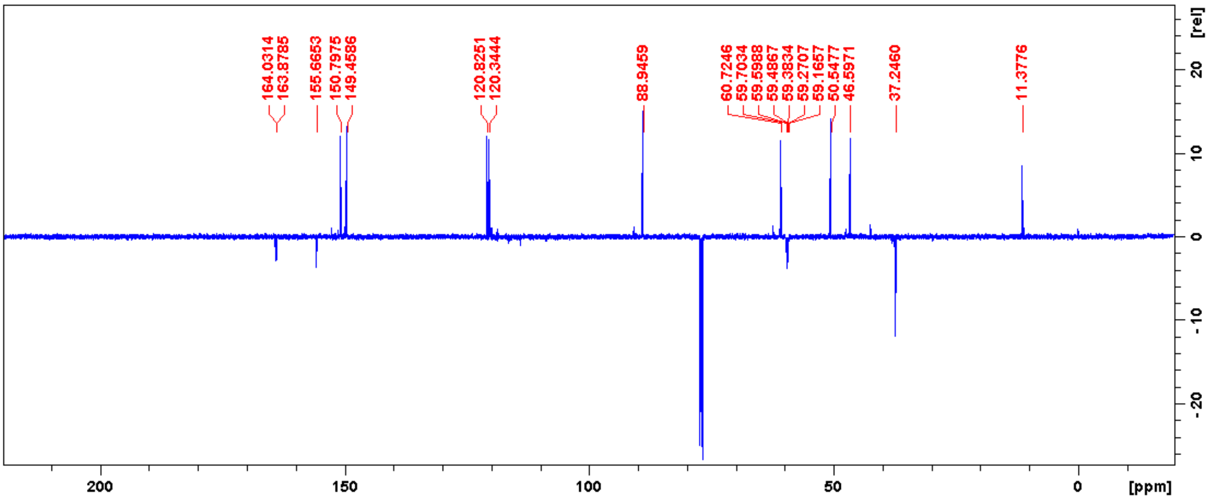

164.0314
163.8785
155.6653
150.7975
149.4586
120.8251
120.3444
88.9459
60.7246
59.7034
59.5988
59.4867
59.3834
59.2707
59.1657
50.5477
46.5971
37.2460
11.3776
[rel]
[ppm]

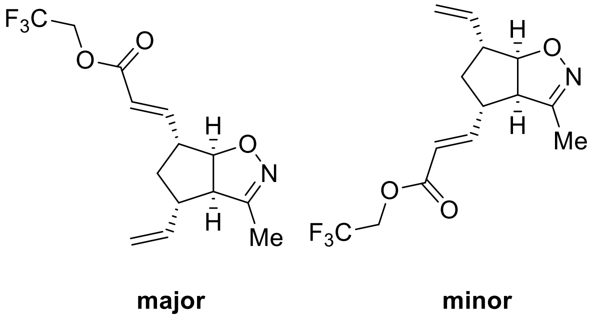
F3C
O
O
H
O
N
H
Me
major
H
O
N
H
Me
O
O
F3C
minor

(±)-**10a** and (±)-**10b**

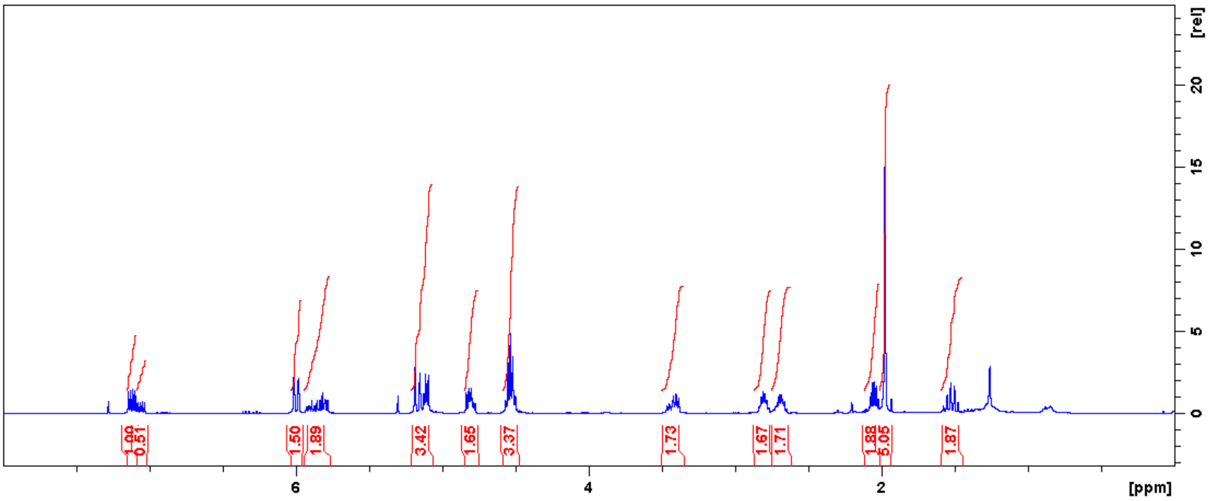
[rel]
20
15
10
5
0
1.00
0.51
1.50
1.89
3.42
1.65
3.37
1.73
1.67
1.71
1.88
5.05
1.87
6
4
2
[ppm]

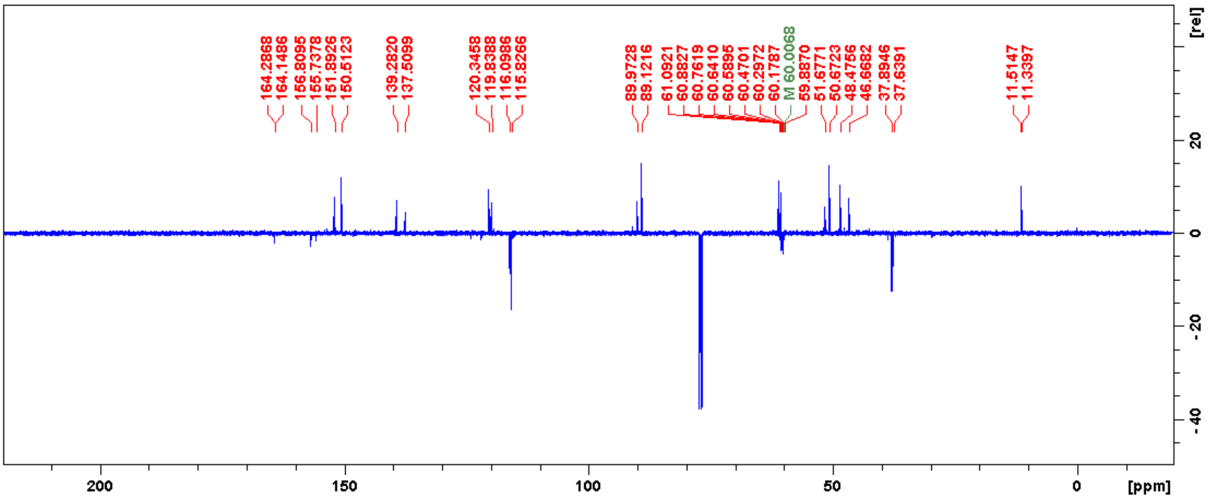
164.2868
164.1486
156.8095
155.7378
151.8926
150.5123
139.2820
137.5099
120.3458
119.8388
116.0986
115.8266
89.9728
89.1216
61.0921
60.8827
60.7619
60.6410
60.5895
60.4701
60.2972
60.1787
M 60.0068
59.8870
51.6771
50.6723
48.4756
46.6682
37.8946
37.6391
11.5147
11.3397
[rel]
20
0
-20
-40
200
150
100
50
0
[ppm]

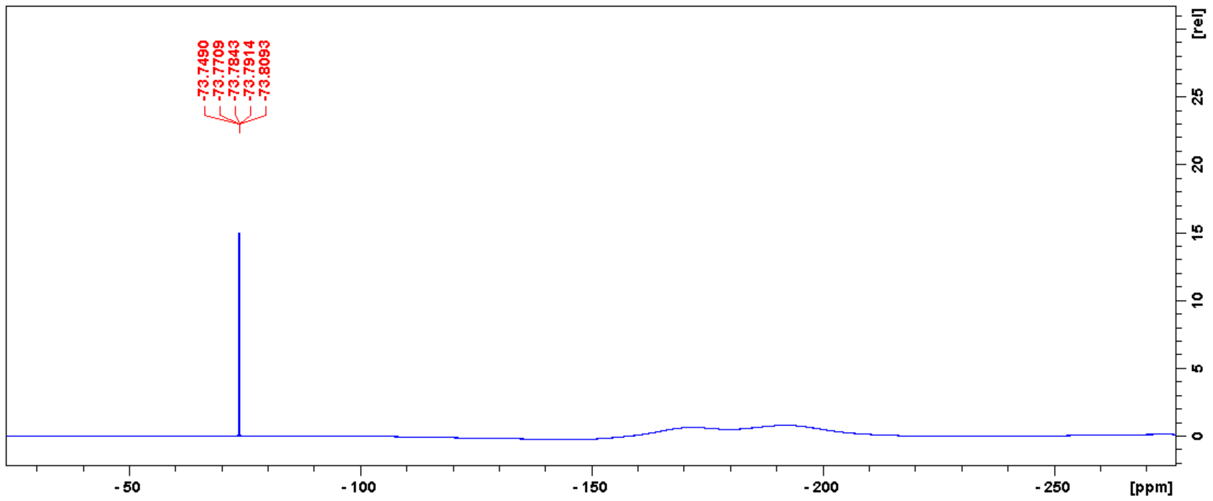
-73.7490
-73.7709
-73.7843
-73.7914
-73.8093
[rel]
25
20
15
10
5
0
- 50
- 100
- 150
- 200
- 250
[ppm]

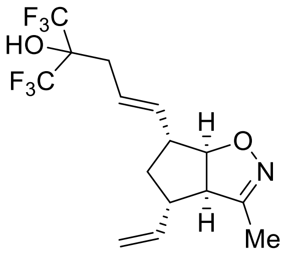

(±)-**11a**

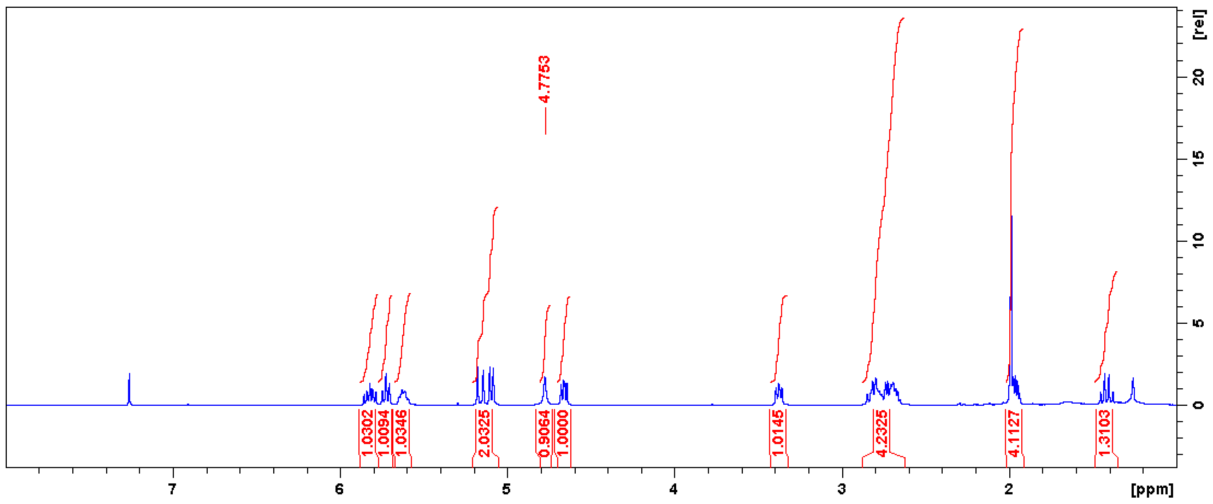

4.7753
1.0302
1.0094
1.0346
2.0325
0.9064
1.0000
1.0145
4.2325
4.1127
1.3103
[ppm]
[rel]

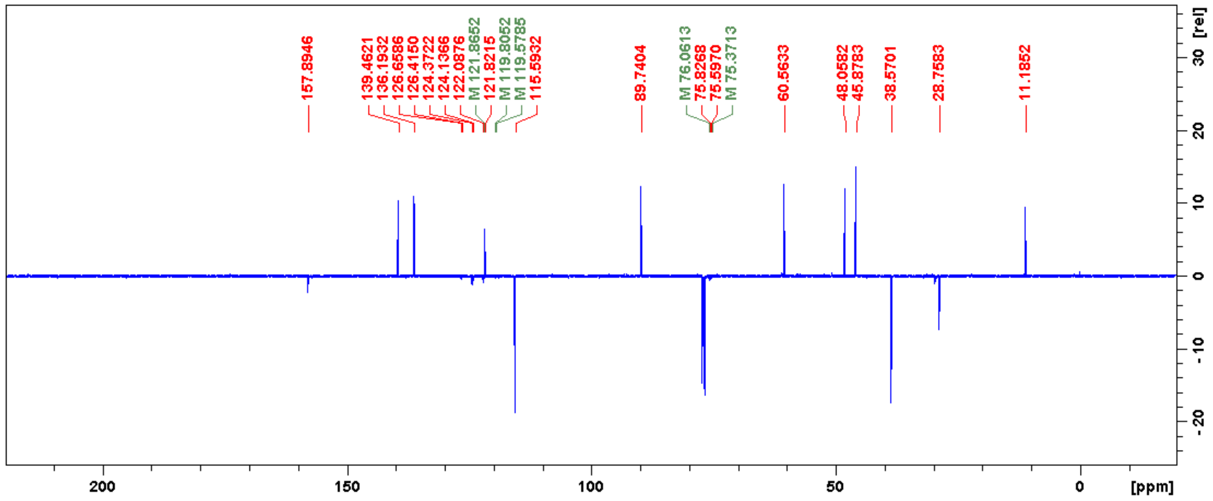

157.8946
139.4621
136.1932
126.6586
126.4150
124.3722
124.1366
122.0876
M 121.8652
121.8215
M 119.8052
M 119.5785
115.5932
89.7404
M 76.0613
75.8268
75.5970
M 75.3713
60.5633
48.0582
45.8783
38.5701
28.7583
11.1852
[ppm]
[rel]

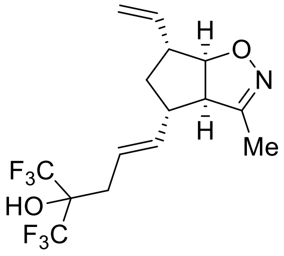

(±)-**11b**

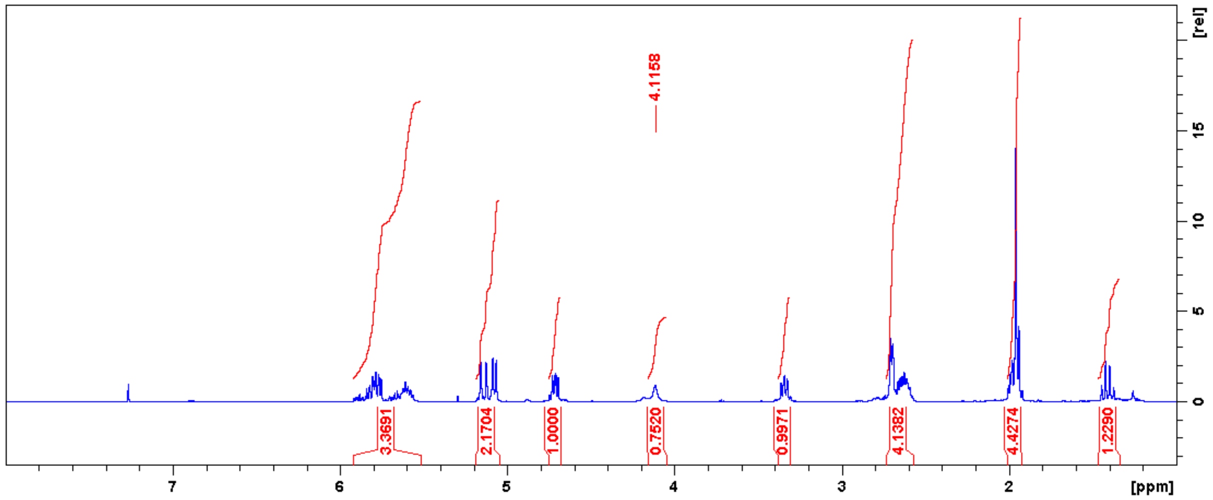
4.1158
3.3691
2.1704
1.0000
0.7520
0.9971
4.1382
4.4274
1.2290
[rel]
[ppm]

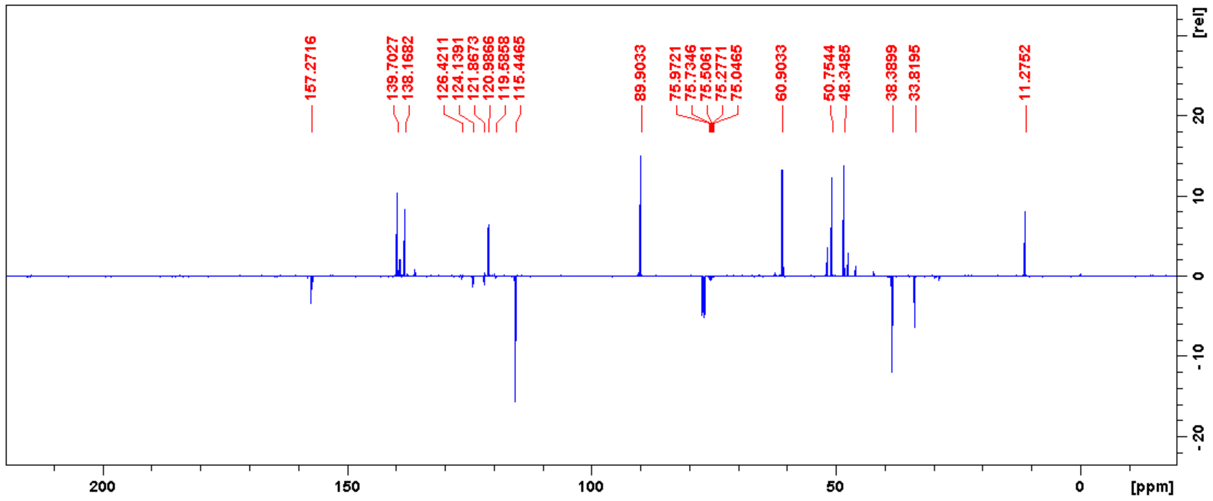
157.2716
139.7027
138.1682
126.4211
124.1391
121.8673
120.9866
119.5858
115.4465
89.9033
75.9721
75.7346
75.5061
75.2771
75.0465
60.9033
50.7544
48.3485
38.3899
33.8195
11.2752
[rel]
[ppm]

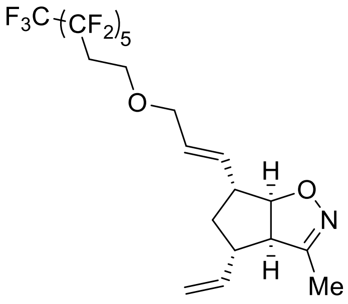

(±)-12a

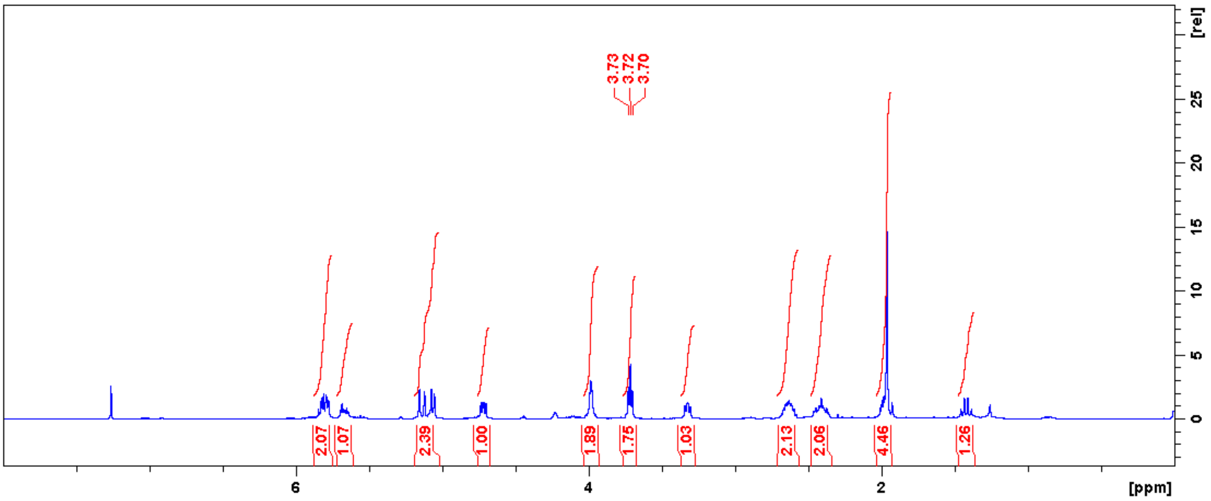

3.73
3.72
3.70
2.07
1.07
2.39
1.00
1.89
1.75
1.03
2.13
2.06
4.46
1.26
[rel]
[ppm]

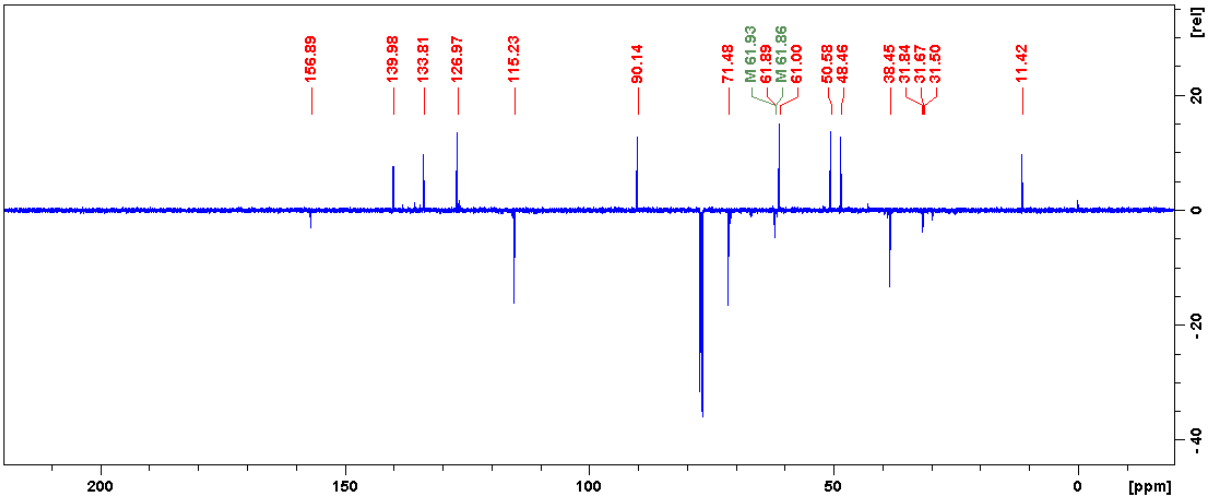

156.89
139.98
133.81
126.97
115.23
90.14
71.48
M 61.93
61.89
M 61.86
61.00
50.58
48.46
38.45
31.84
31.67
31.50
11.42
[rel]
[ppm]

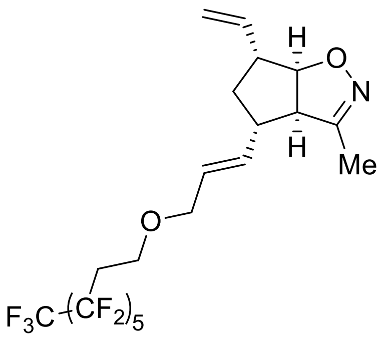

H
O
N
H
Me
O
F3C
(CF2)5

(±)-**12b**

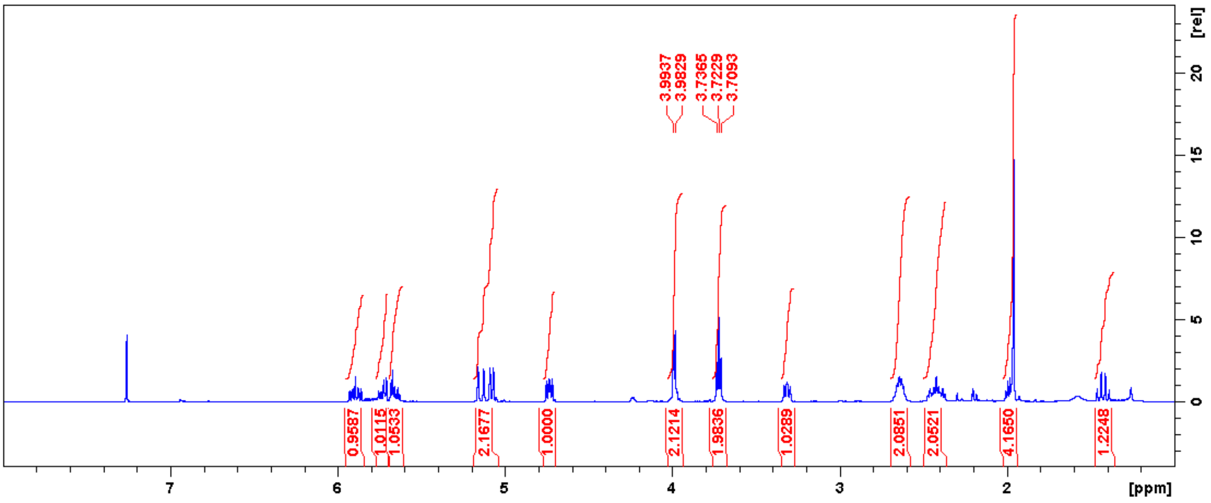

3.9937
3.9829
3.7365
3.7229
3.7093
0.9587
1.0115
1.0533
2.1677
1.0000
2.1214
1.9836
1.0289
2.0851
2.0521
4.1650
1.2248
7
6
5
4
3
2
[ppm]
[rel]
20
15
10
5
0

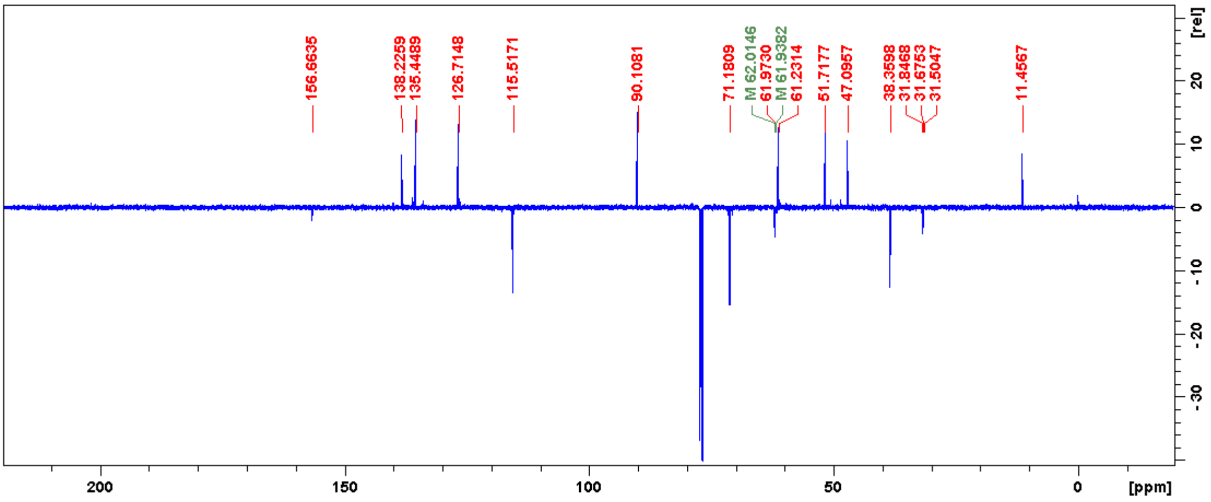

156.6635
138.2259
135.4489
126.7148
115.5171
90.1081
71.1809
M 62.0146
61.9730
M 61.9382
61.2314
51.7177
47.0957
38.3598
31.8468
31.6753
31.5047
11.4567
200
150
100
50
0
[ppm]
[rel]
20
10
0
-10
-20
-30

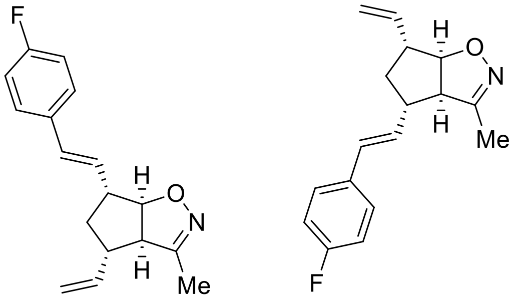

major **minor** (±)-**13a** and (±)-**13b**

Spectra in D_6-benzene:

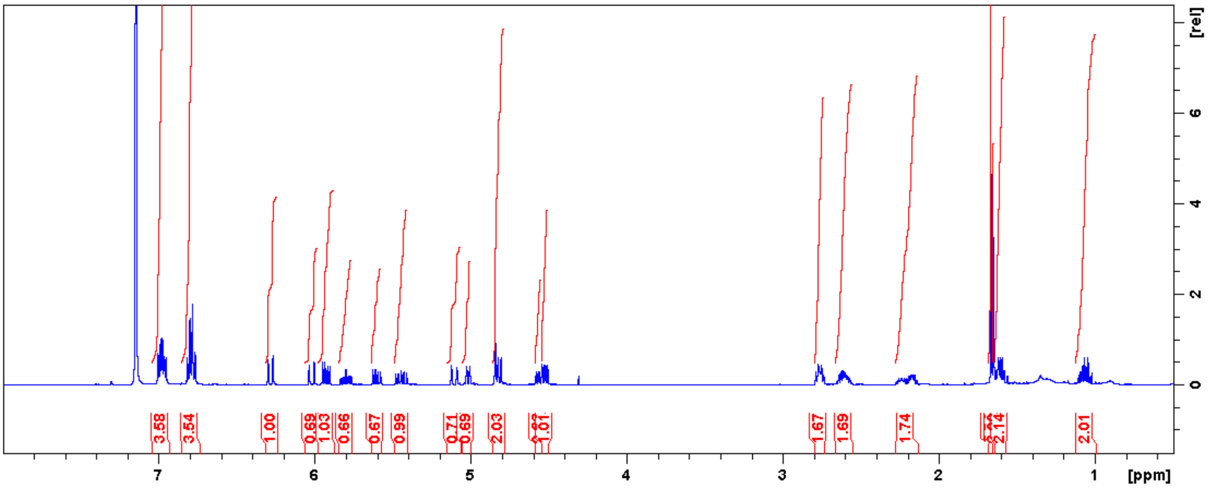

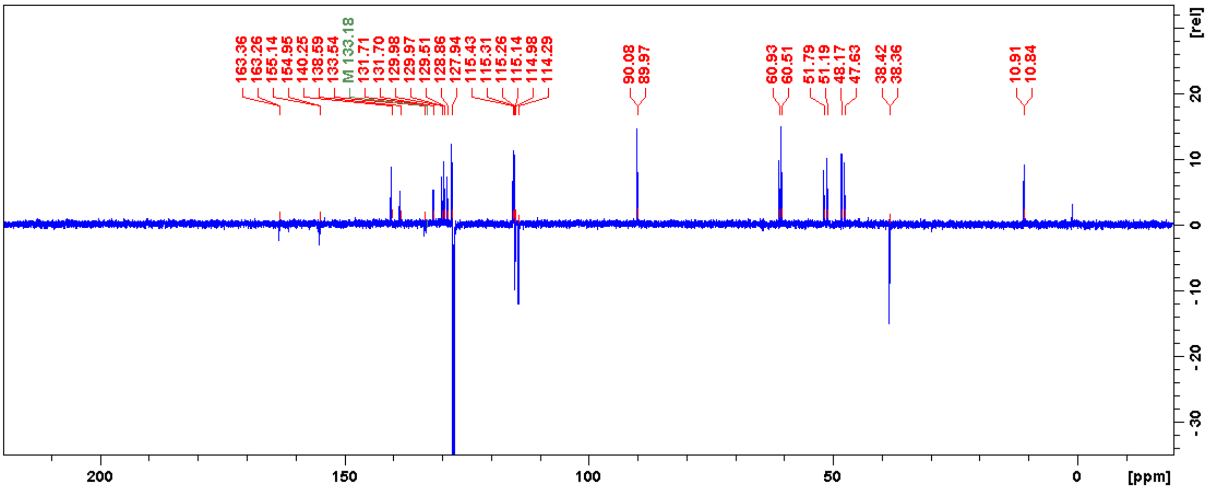

Spectra in $CDCl_3$:

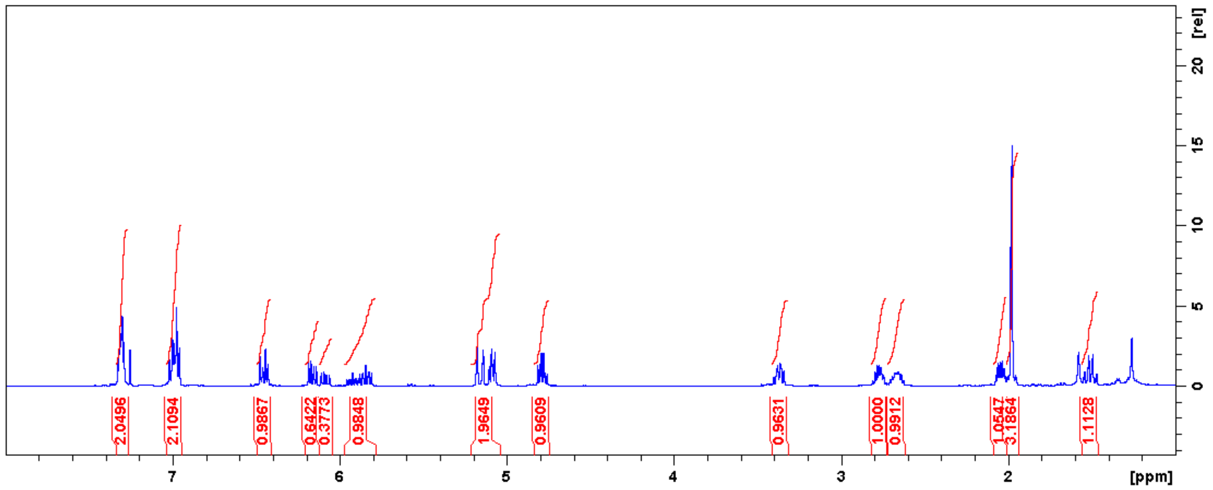

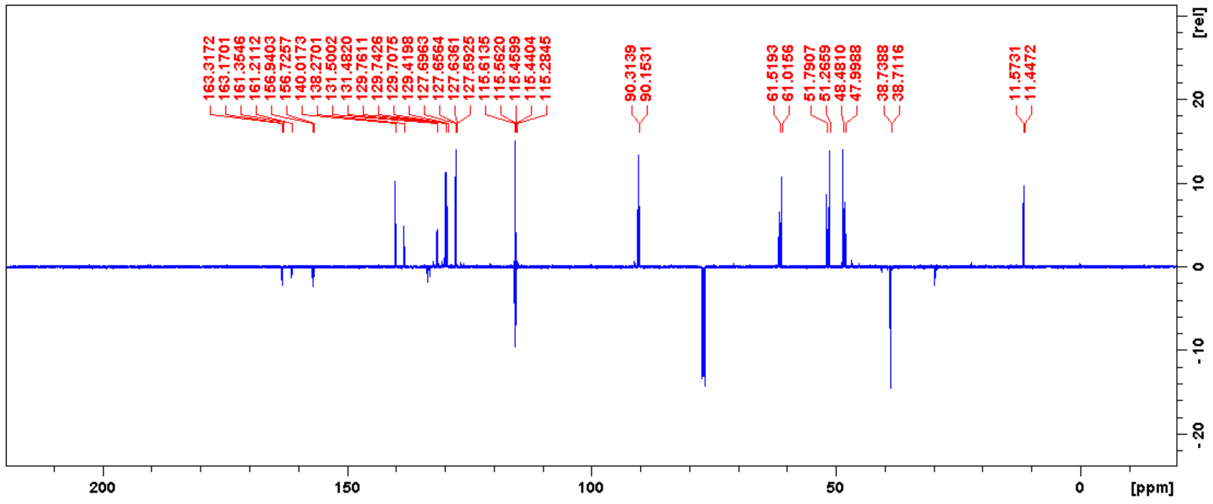

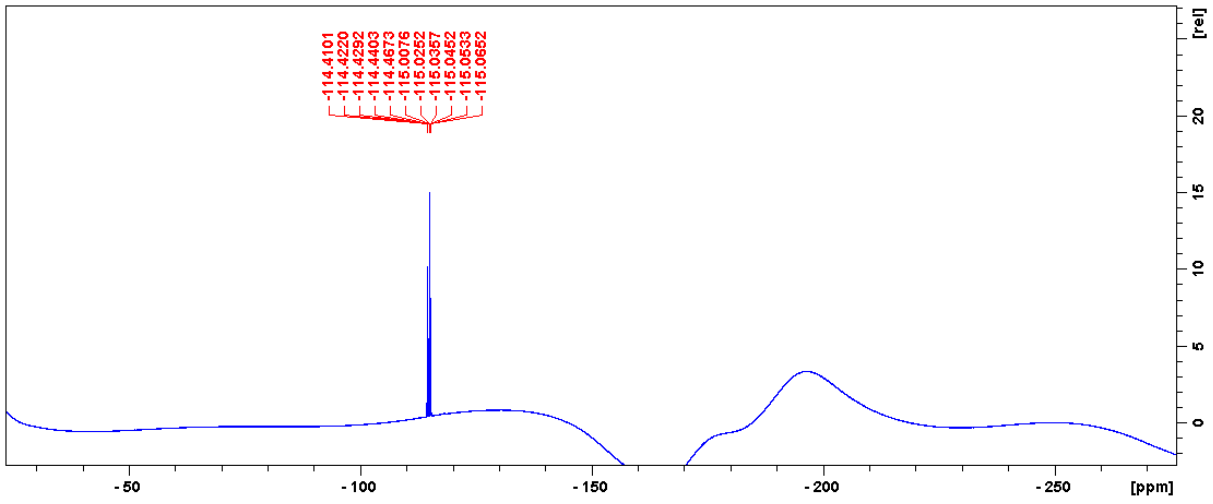

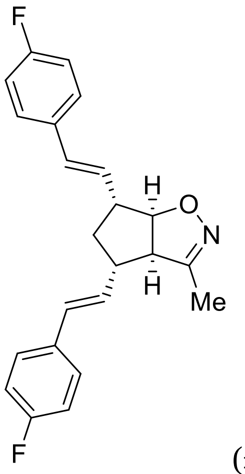
F
H
O
N
H
Me
F

(±)-**13c**

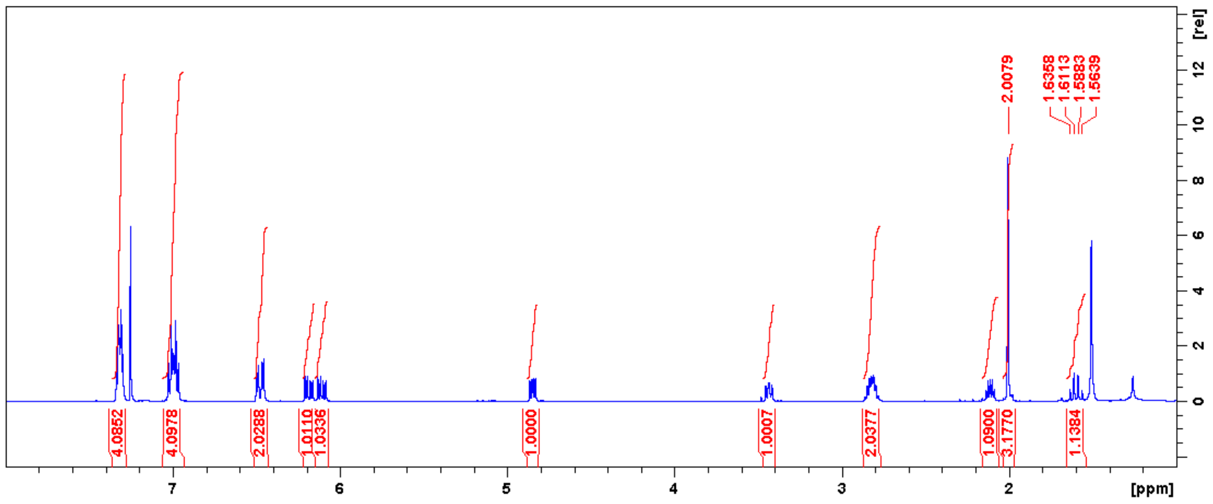
2.0079
1.6358
1.6113
1.5883
1.5639
4.0852
4.0978
2.0288
1.0110
1.0336
1.0000
1.0007
2.0377
1.0900
3.1770
1.1384
[rel]
[ppm]

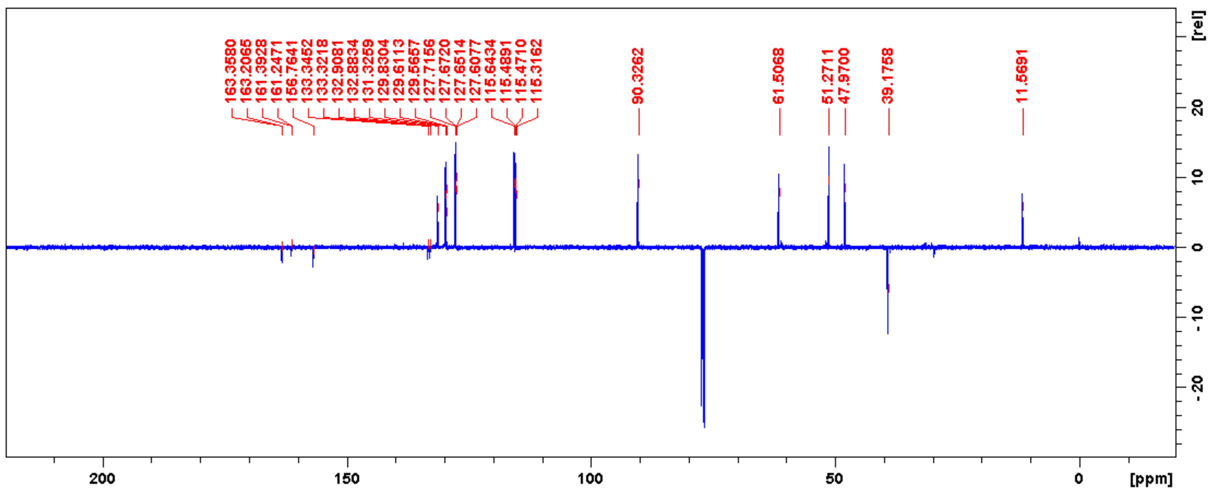
163.3580
163.2065
161.3928
161.2471
156.7641
133.3452
133.3218
132.9081
132.8834
131.3259
129.8304
129.6113
129.5657
127.7156
127.6720
127.6514
127.6077
115.6434
115.4891
115.4710
115.3162
90.3262
61.5068
51.2711
47.9700
39.1758
11.5691
[rel]
[ppm]

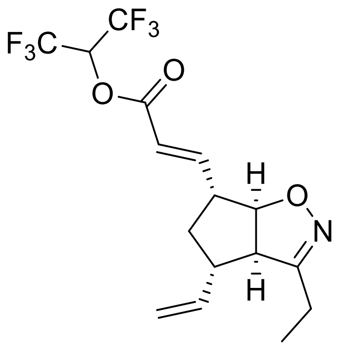
CF3
F3C
O
O
H
O
N
H

(±)-**14a**

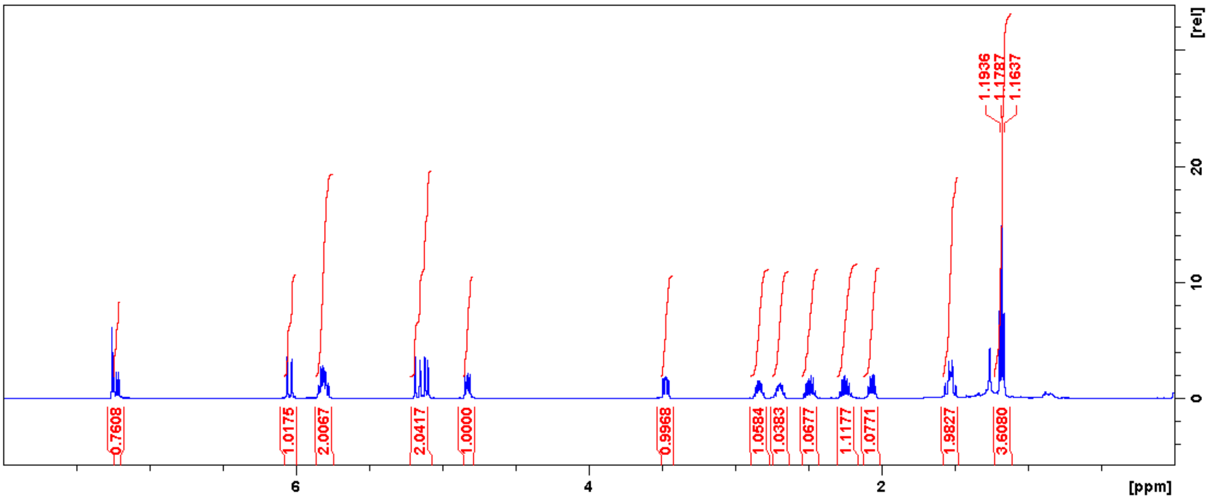
1.1936
1.1787
1.1637
0.7608
1.0175
2.0067
2.0417
1.0000
0.9968
1.0584
1.0383
1.0677
1.1177
1.0771
1.9827
3.6080
[rel]
[ppm]

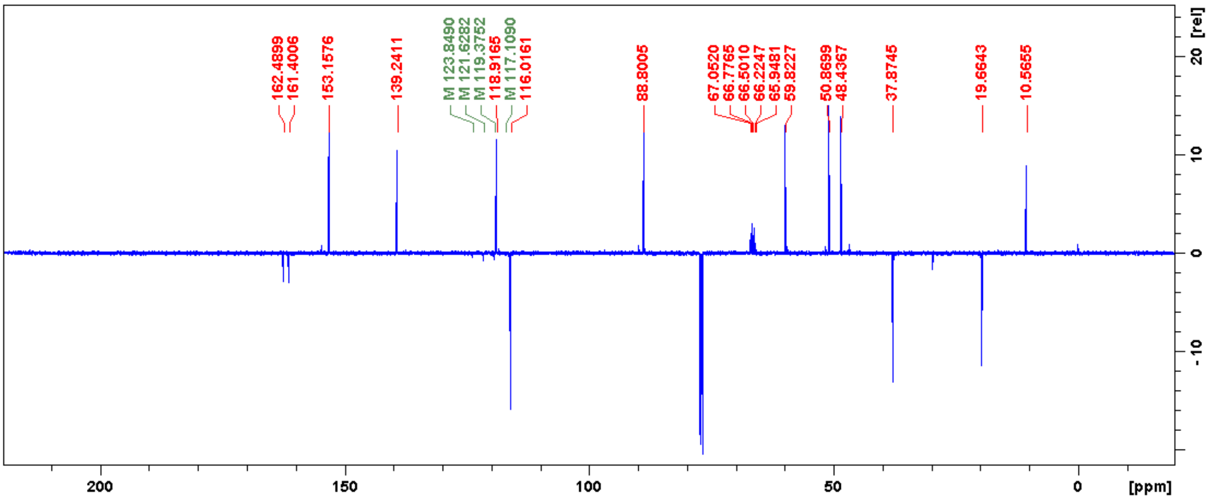
162.4899
161.4006
153.1576
139.2411
M 123.8490
M 121.6282
M 119.3752
118.9165
M 117.1090
116.0161
88.8005
67.0520
66.7765
66.5010
66.2247
65.9481
59.8227
50.8699
48.4367
37.8745
19.6643
10.5655
[rel]
[ppm]

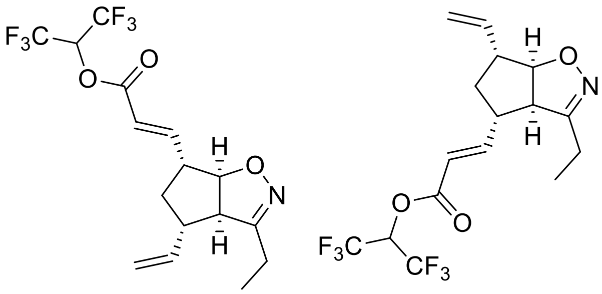

major **minor** (±)-**14a** and (±)-**14b**

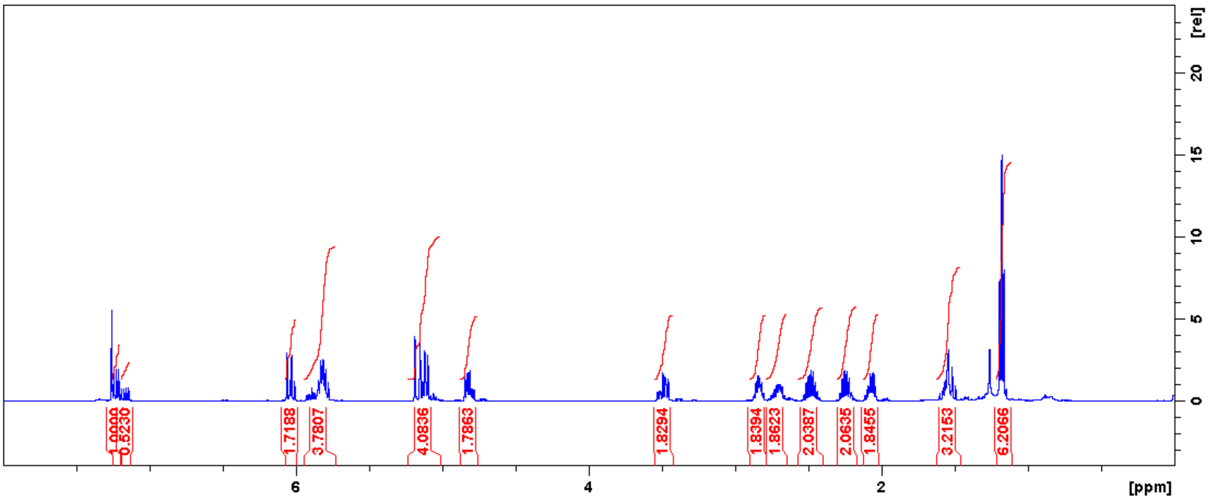

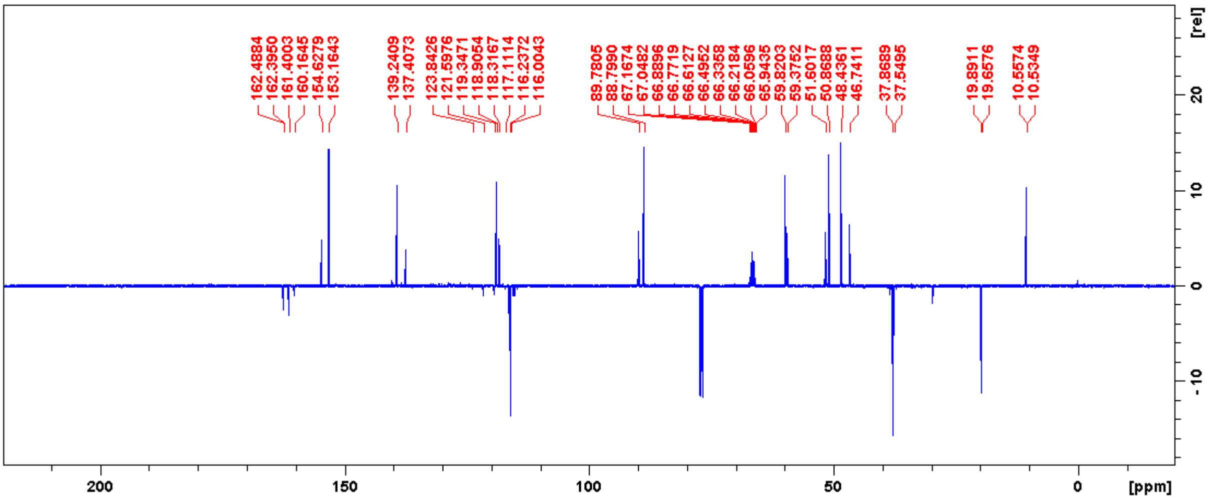

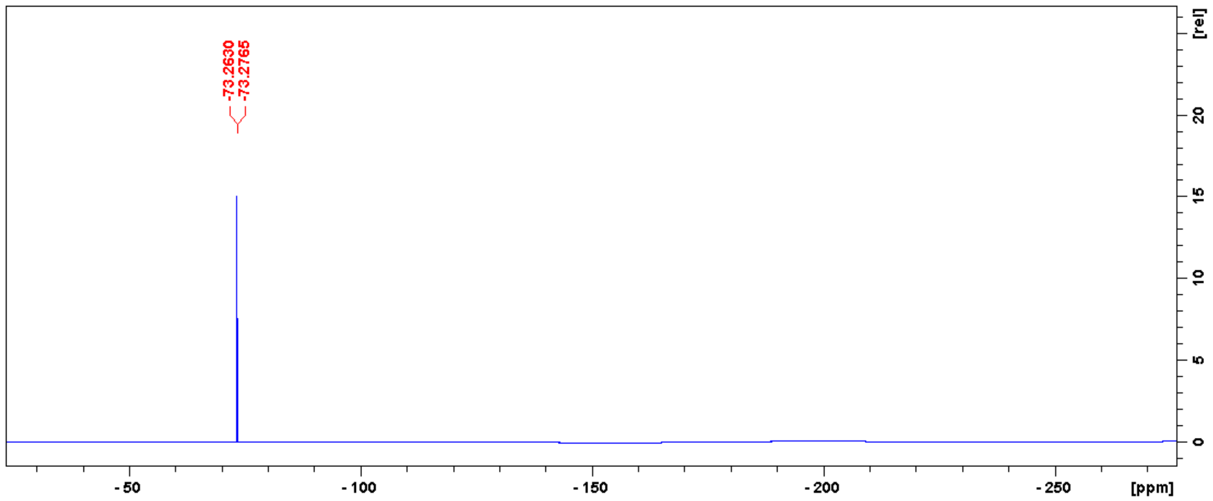
-73.2630
-73.2765
[rel]
20
15
10
5
0
-50
-100
-150
-200
-250
[ppm]

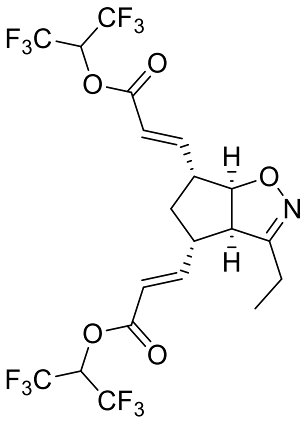

(±)-**14c**

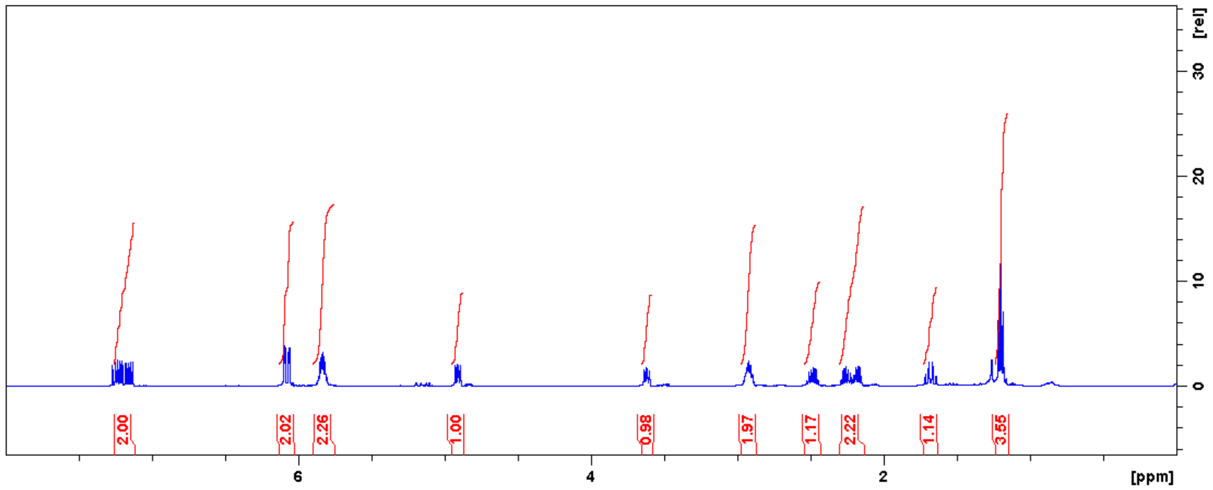

2.00
2.02
2.26
1.00
0.98
1.97
1.17
2.22
1.14
3.55
[ppm]
[rel]

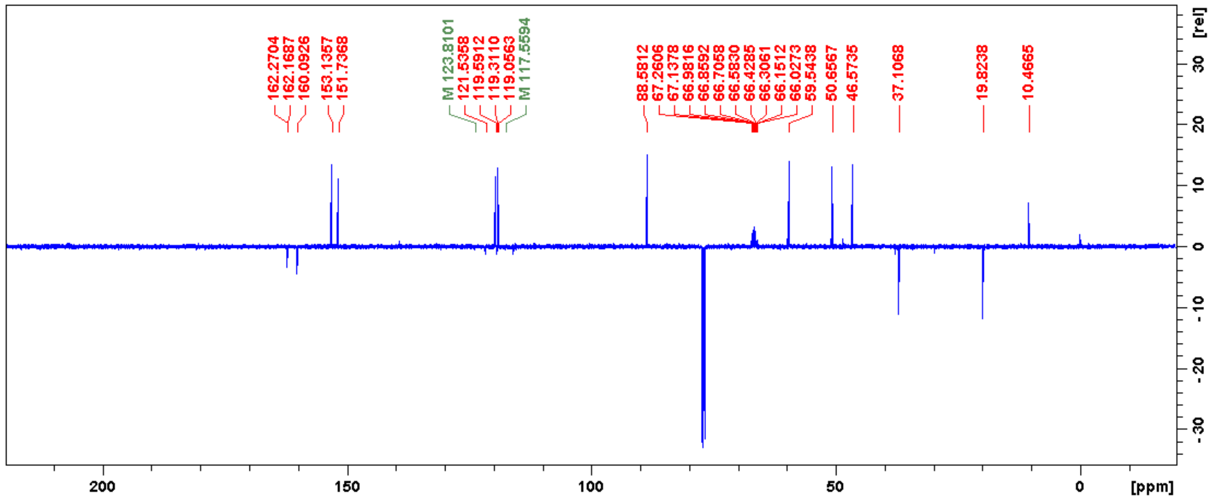

162.2704
162.1687
160.0926
153.1357
151.7368
M 123.8101
121.5358
119.5912
119.3110
119.0563
M 117.5594
88.5812
67.2606
67.1378
66.9816
66.8592
66.7058
66.5830
66.4285
66.3061
66.1512
66.0273
59.5438
50.6567
46.5735
37.1068
19.8238
10.4665
[ppm]
[rel]

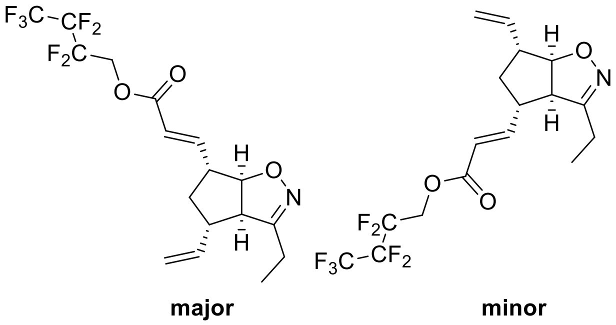
F3C
CF2
F2C
O
O
H
O
N
H
major
H
O
N
H
O
O
F2C
F3C
CF2
minor

(±)-**15a** and (±)-**15b**

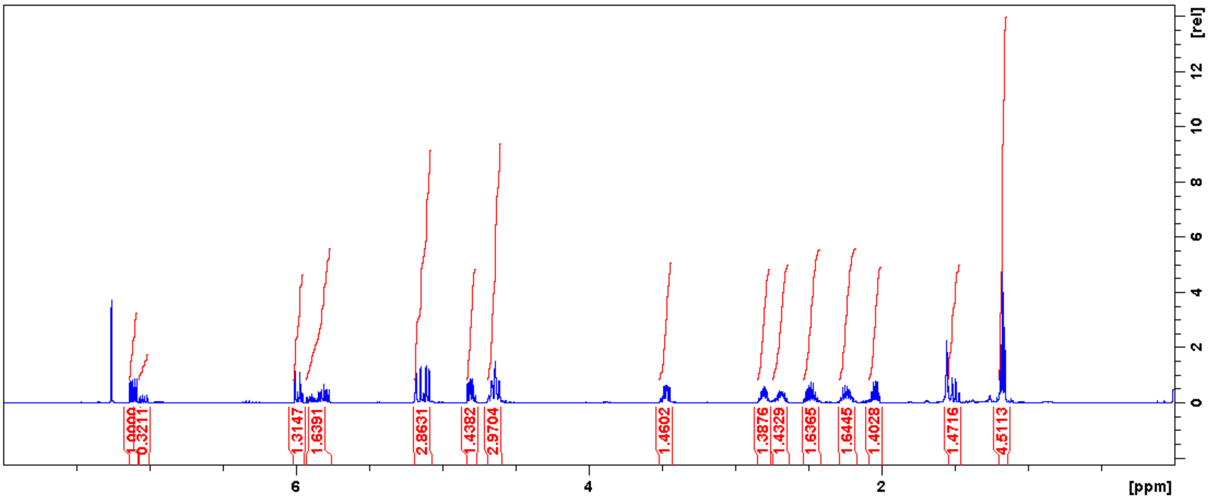
1.0000
0.3211
1.3147
1.6391
2.8631
1.4382
2.9704
1.4602
1.3876
1.4329
1.6365
1.6445
1.4028
1.4716
4.5113
6
4
2
[ppm]
[rel]

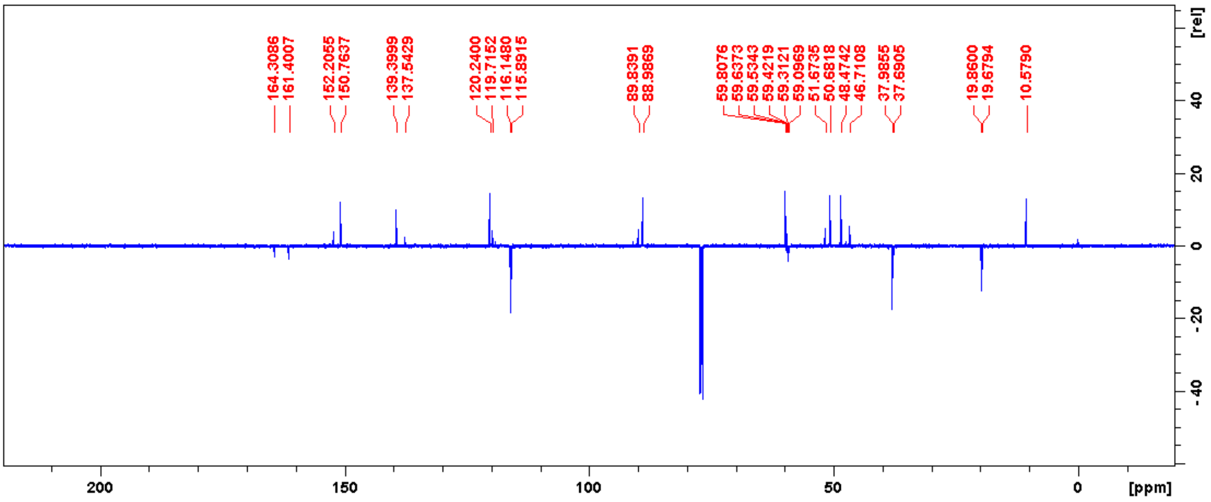
164.3086
161.4007
152.2055
150.7637
139.3999
137.5429
120.2400
119.7152
116.1480
115.8915
89.8391
88.9869
59.8076
59.6373
59.5343
59.4219
59.3121
59.0969
51.6735
50.6818
48.4742
46.7108
37.9855
37.6905
19.8600
19.6794
10.5790
200
150
100
50
0
[ppm]
[rel]

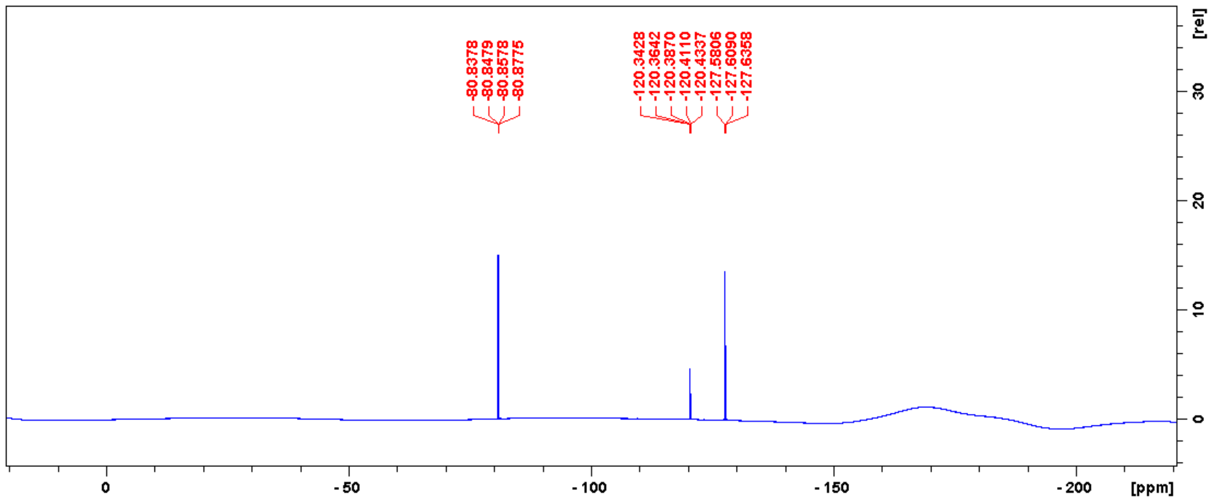

-80.8378
-80.8479
-80.8578
-80.8775
-120.3428
-120.3642
-120.3870
-120.4110
-120.4337
-127.5806
-127.6090
-127.6358
[rel]
30
20
10
0
0
- 50
- 100
- 150
- 200
[ppm]

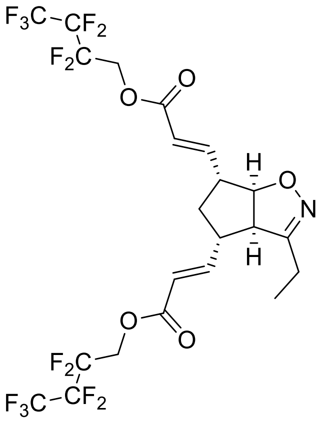

(±)-15c

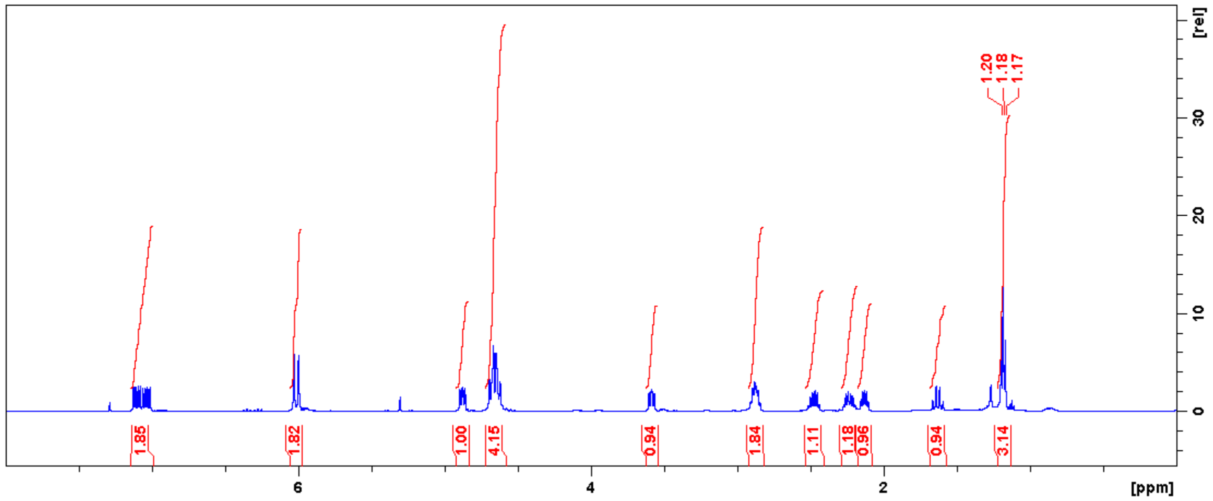

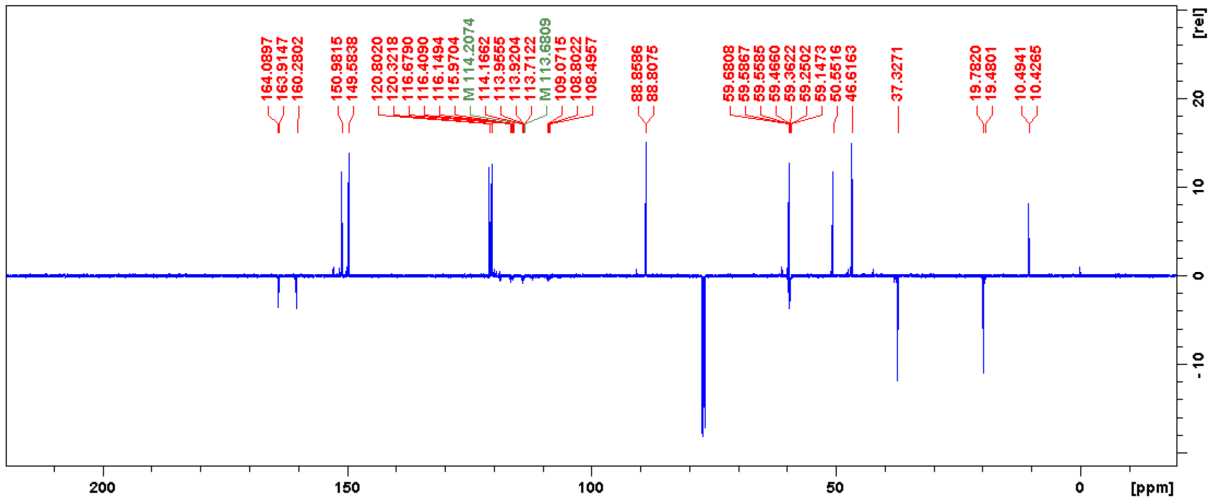

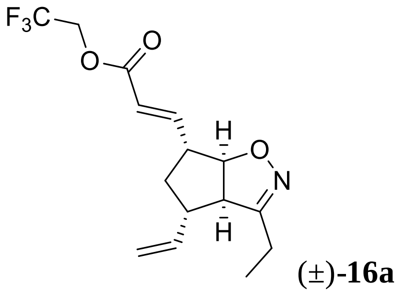
F3C
O
O
H
O
N
H

(±)-**16a**

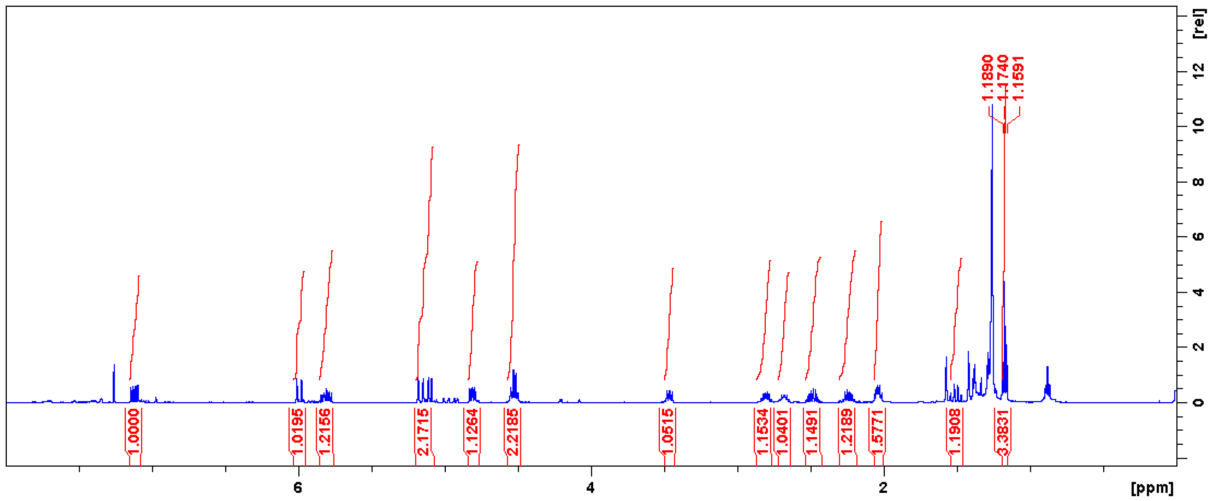
1.1890
1.1740
1.1591
1.0000
1.0195
1.2156
2.1715
1.1264
2.2185
1.0515
1.1534
1.0401
1.1491
1.2189
1.5771
1.1908
3.3831
[rel]
[ppm]

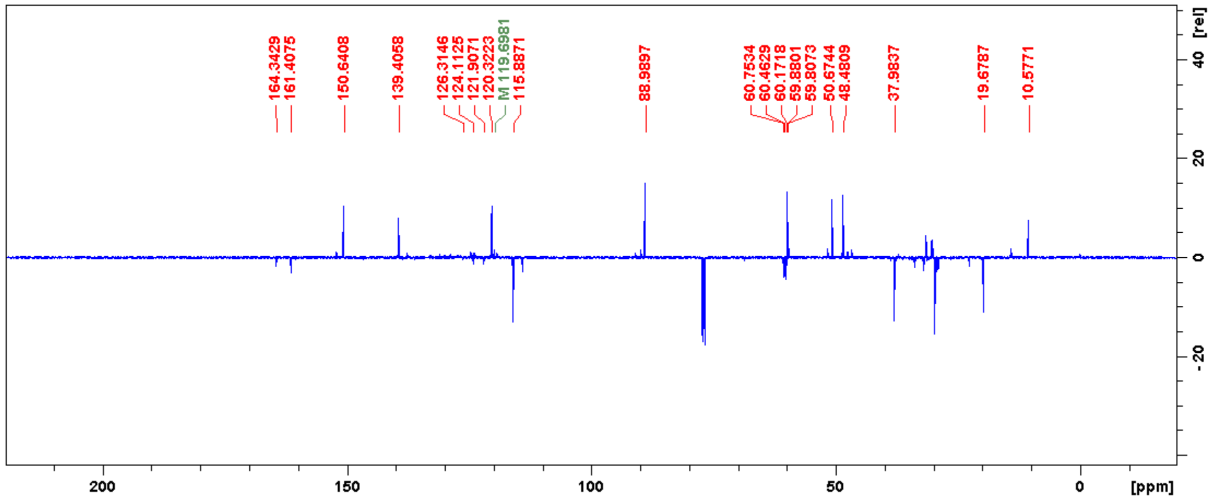
164.3429
161.4075
150.6408
139.4058
126.3146
124.1125
121.9071
120.3223
M 119.6981
115.8871
88.9897
60.7534
60.4629
60.1718
59.8801
59.8073
50.6744
48.4809
37.9837
19.6787
10.5771
[rel]
[ppm]

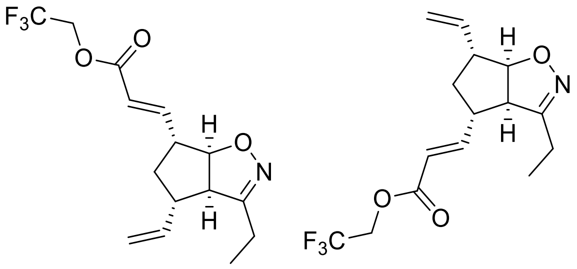

major **minor** (±)-**16a** and (±)-**16b**

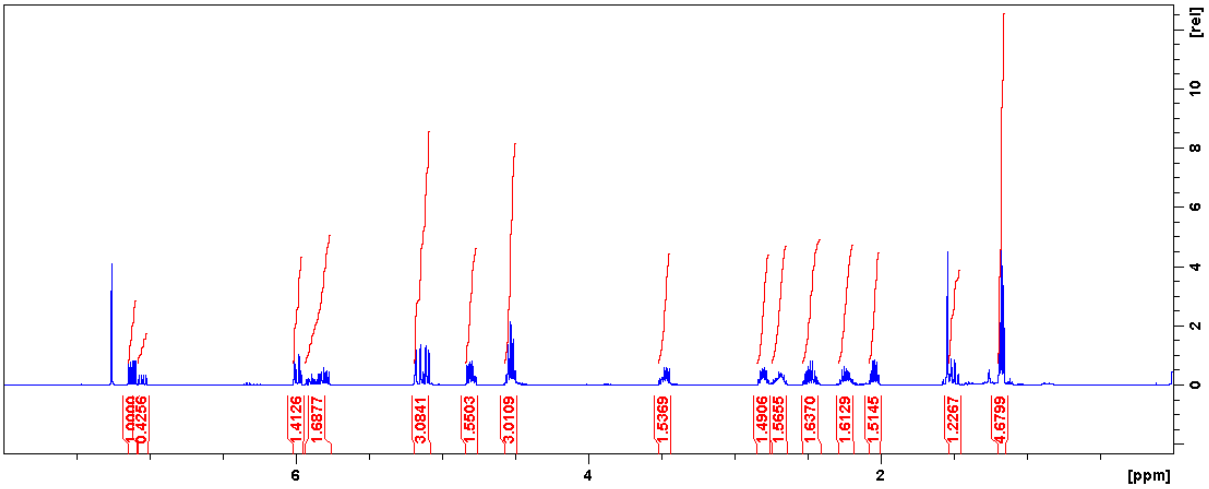

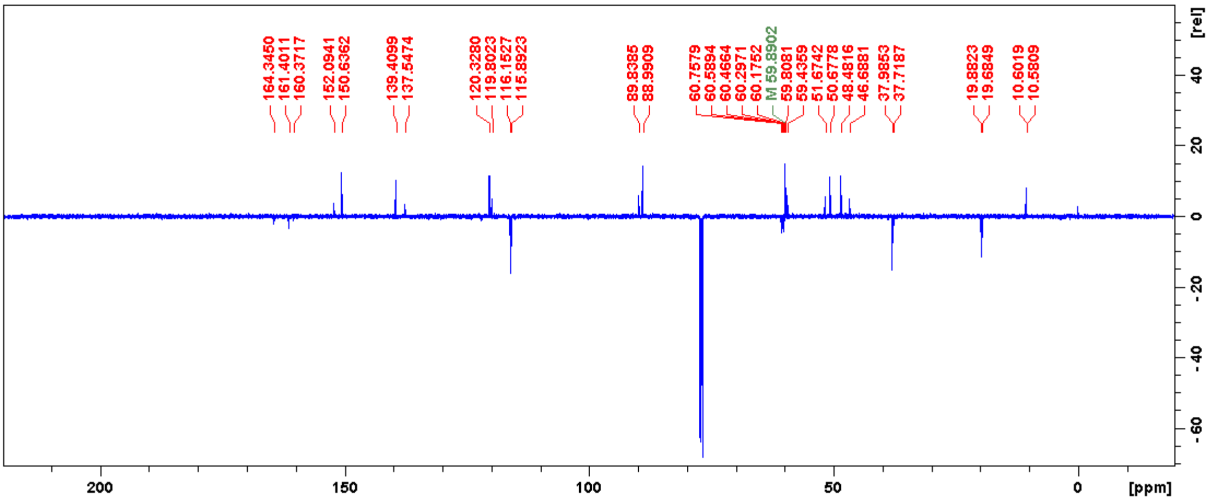

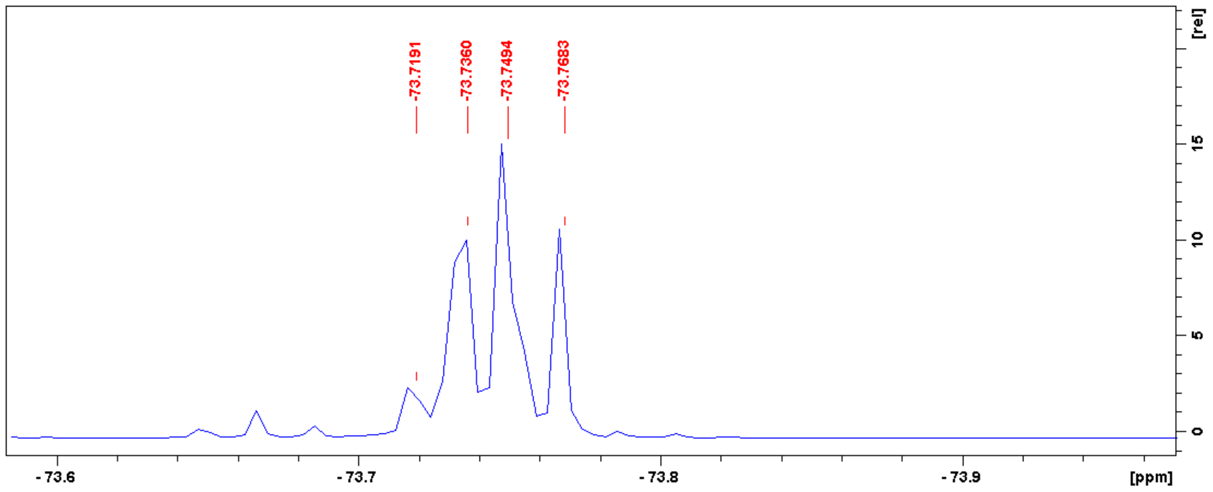
-73.7191
-73.7360
-73.7494
-73.7683
[rel]
15
10
5
0
- 73.6
- 73.7
- 73.8
- 73.9
[ppm]

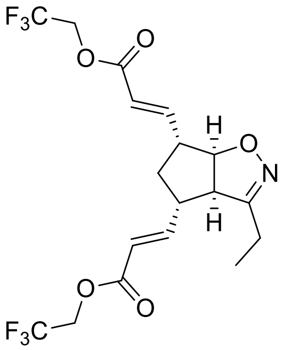
F3C
O
O
H
O
N
H
O
O
F3C

(±)-**16c**

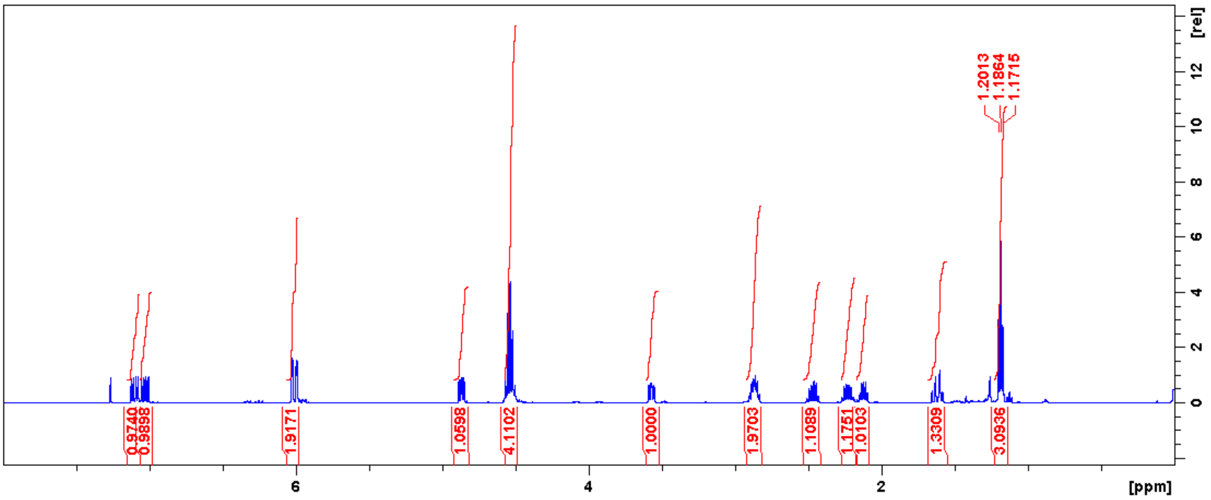
1.2013
1.1864
1.1715
0.9740
0.9898
1.9171
1.0598
4.1102
1.0000
1.9703
1.1089
1.1751
1.0103
1.3309
3.0936
[rel]
[ppm]

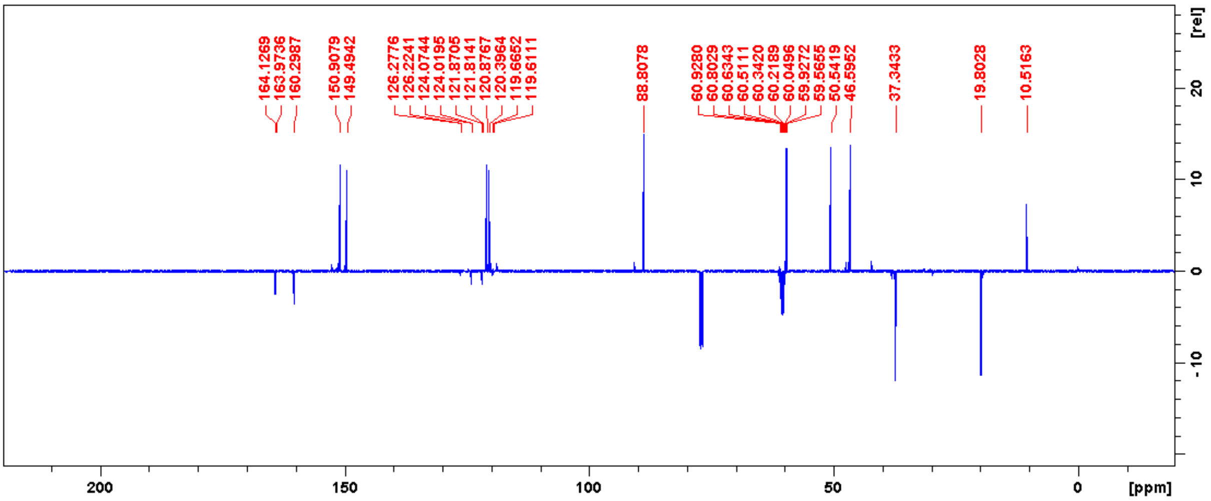
164.1269
163.9736
160.2987
150.9079
149.4942
126.2776
126.2241
124.0744
124.0195
121.8705
121.8141
120.8767
120.3964
119.6652
119.6111
88.8078
60.9280
60.8029
60.6343
60.5111
60.3420
60.2189
60.0496
59.9272
59.5655
50.5419
46.5952
37.3433
19.8028
10.5163
[rel]
[ppm]

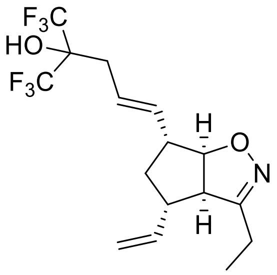
F3C
HO
F3C
H
O
N
H

(±)-**17a**

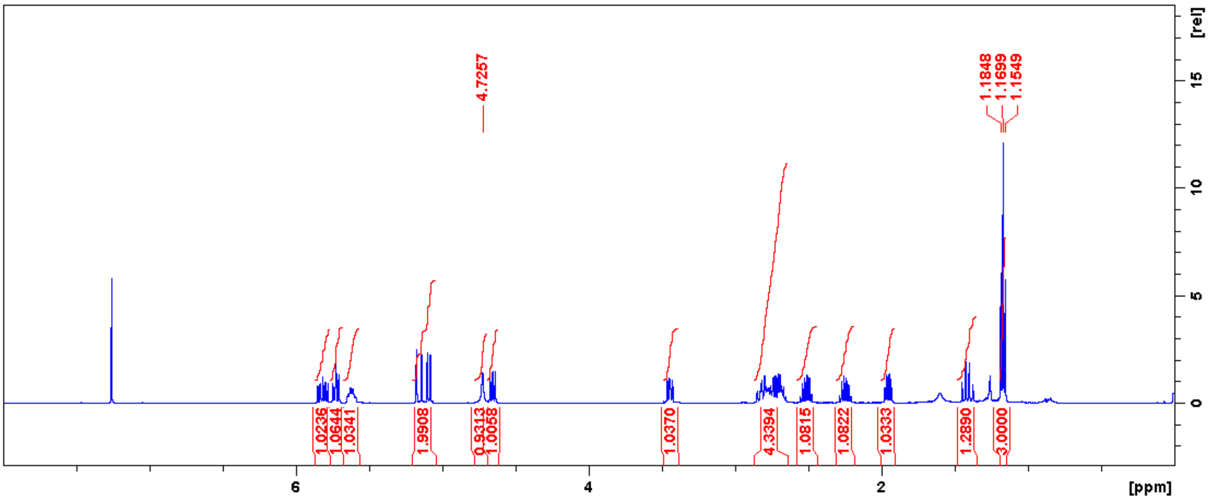
4.7257
1.1848
1.1699
1.1549
1.0236
1.0644
1.0341
1.9908
0.9313
1.0058
1.0370
4.3394
1.0815
1.0822
1.0333
1.2890
3.0000
[rel]
[ppm]

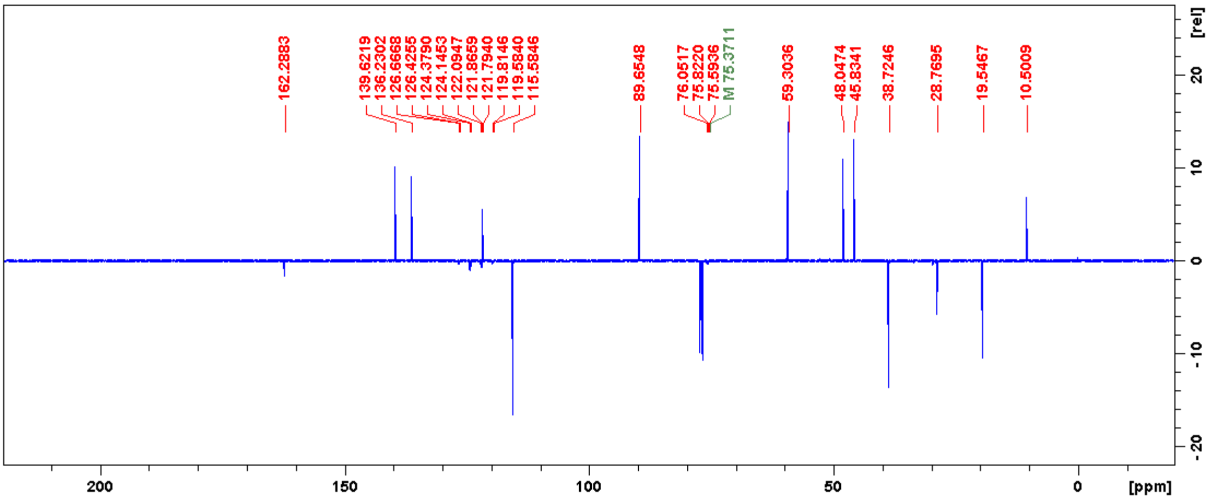
162.2883
139.6219
136.2302
126.6668
126.4255
124.3790
124.1453
122.0947
121.8659
121.7940
119.8146
119.5840
115.5846
89.6548
76.0517
75.8220
75.5936
M 75.3711
59.3036
48.0474
45.8341
38.7246
28.7695
19.5467
10.5009
[rel]
[ppm]

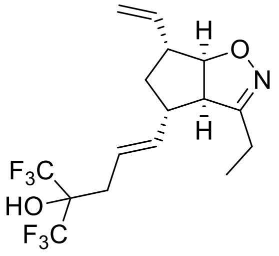
H
O
N
H
F3C
HO
F3C

(±)-**17b**

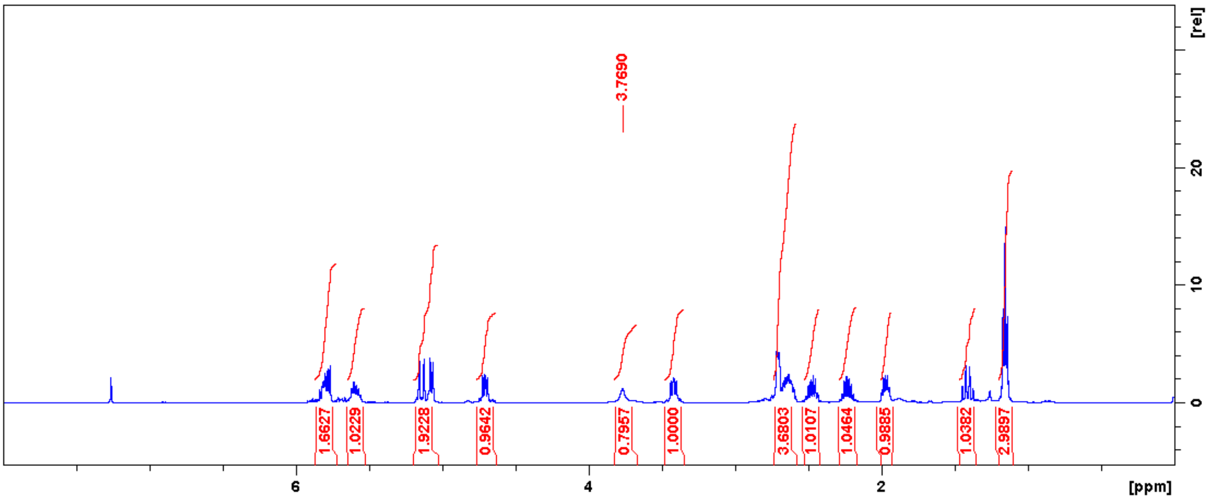
3.7690
1.6627
1.0229
1.9228
0.9642
0.7957
1.0000
3.6803
1.0107
1.0464
0.9885
1.0382
2.9897
[rel]
20
10
0
6
4
2
[ppm]

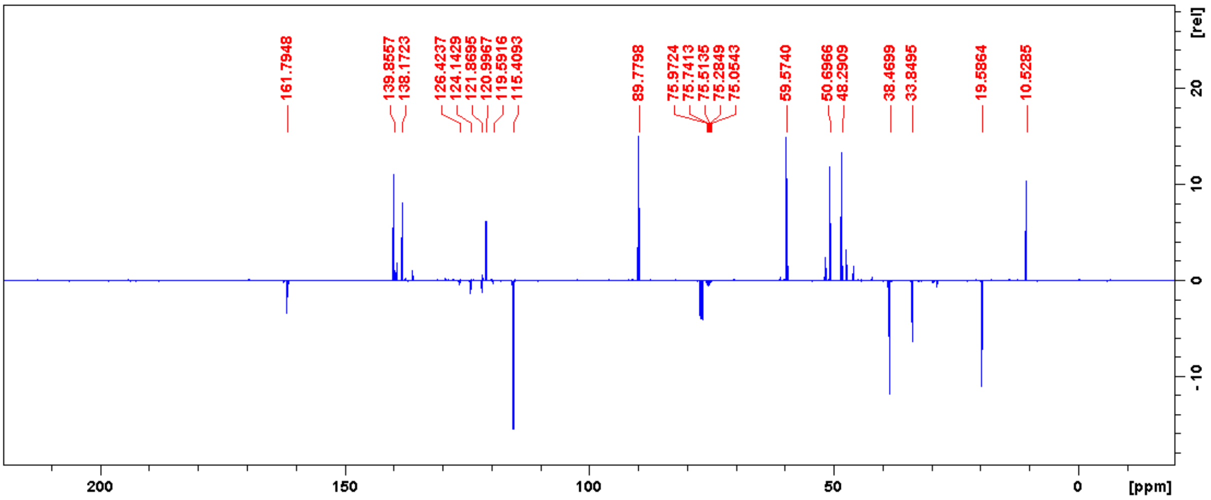
161.7948
139.8557
138.1723
126.4237
124.1429
121.8695
120.9967
119.5916
115.4093
89.7798
75.9724
75.7413
75.5135
75.2849
75.0543
59.5740
50.6966
48.2909
38.4699
33.8495
19.5864
10.5285
[rel]
20
10
0
-10
200
150
100
50
0
[ppm]

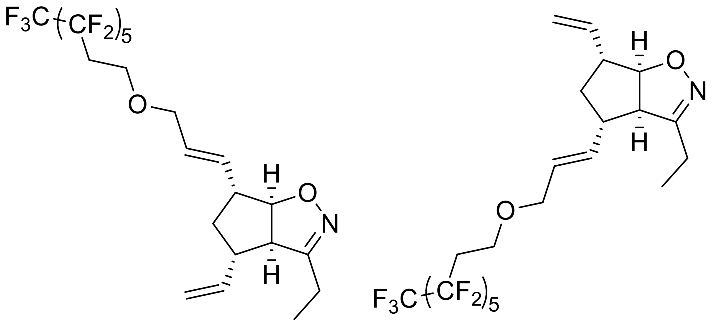
F3C(CF2)5
O
H
O
N
H
F3C(CF2)5

(±)-**18a** and (±)-**18b**

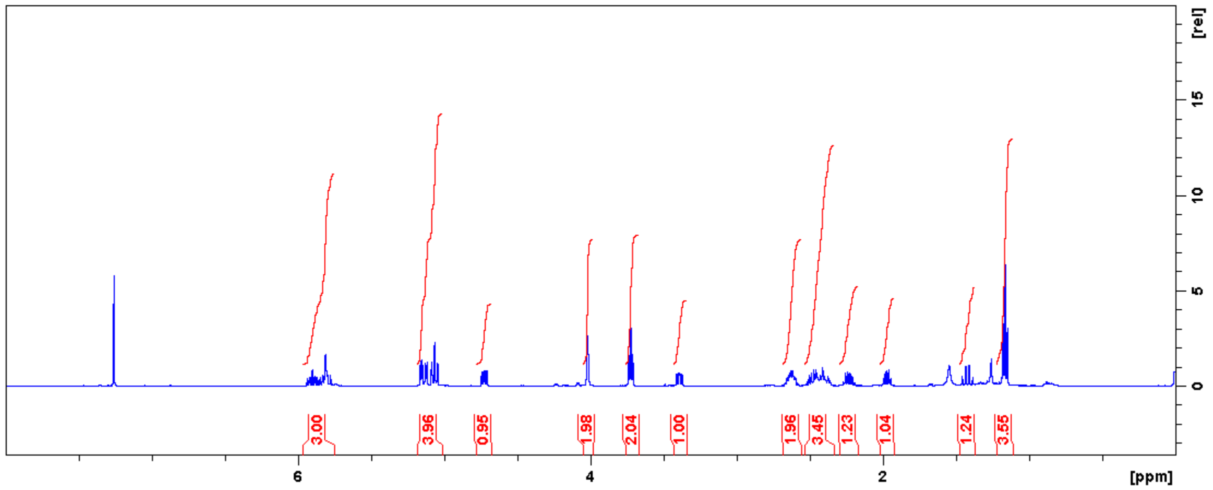
3.00
3.96
0.95
1.98
2.04
1.00
1.96
3.45
1.23
1.04
1.24
3.55
6
4
2
[ppm]
[rel]
0
5
10
15

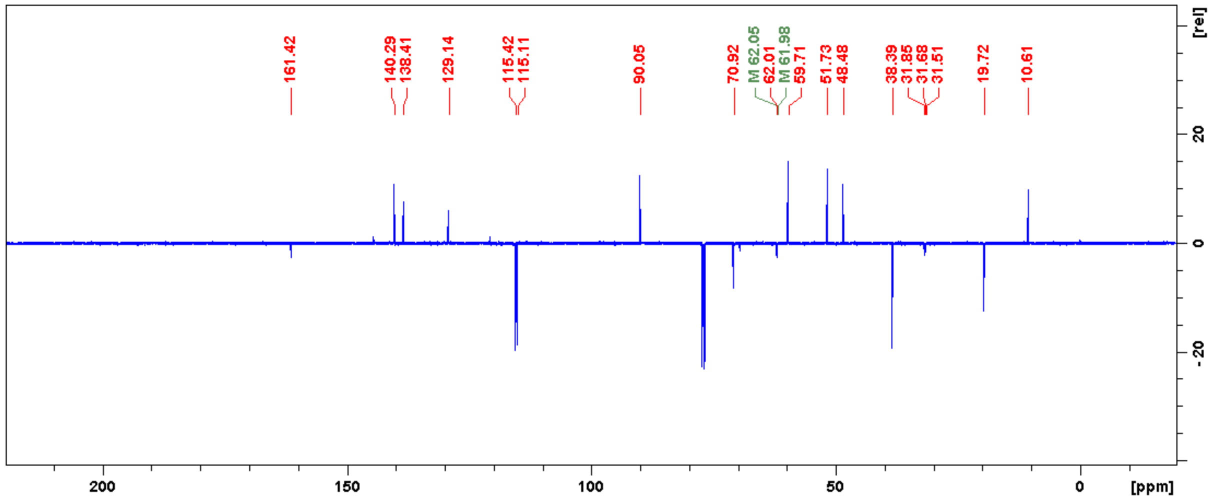
161.42
140.29
138.41
129.14
115.42
115.11
90.05
70.92
M 62.05
62.01
M 61.98
59.71
51.73
48.48
38.39
31.85
31.68
31.51
19.72
10.61
200
150
100
50
0
[ppm]
[rel]
20
0
-20

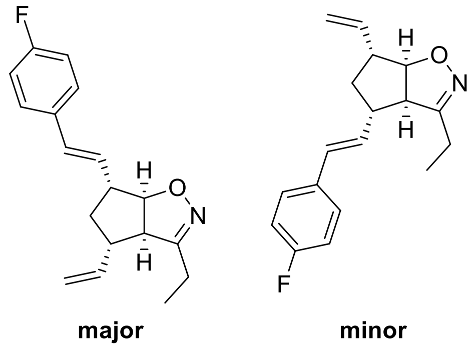

(±)-**19a** and (±)-**19b**

Spectra in D_6-benzene:

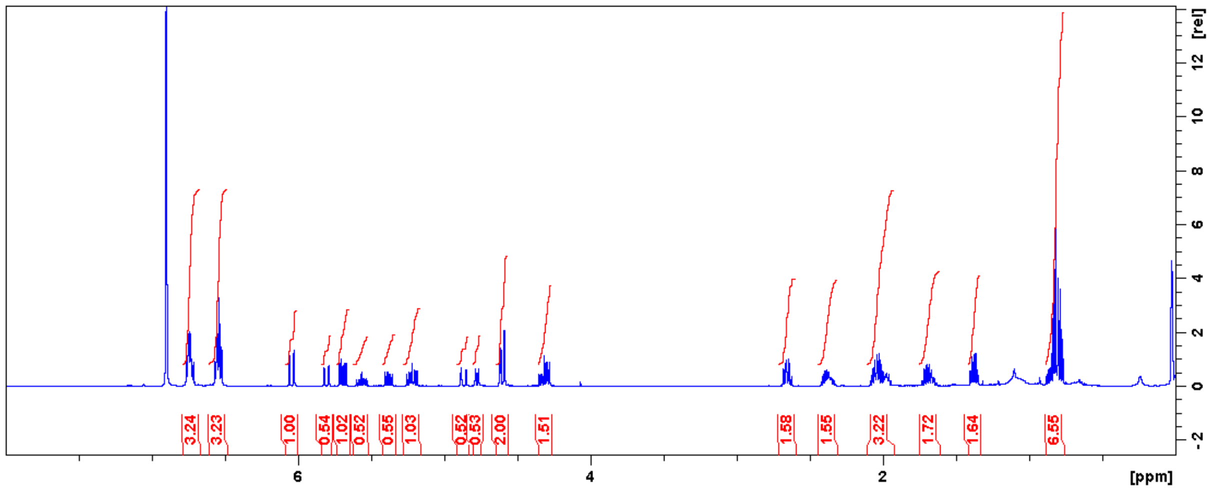

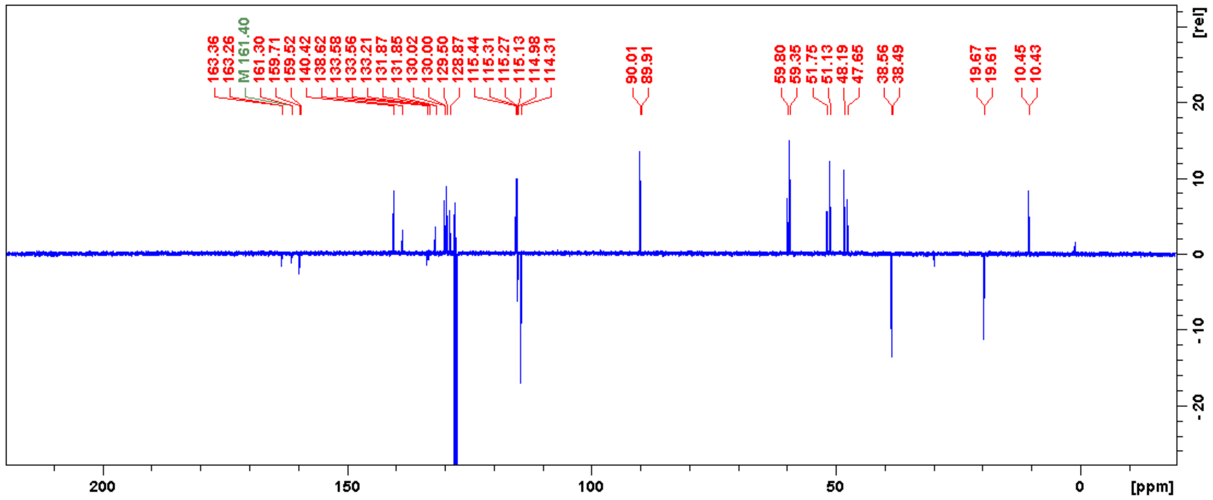

Spectra in $CDCl_3$:

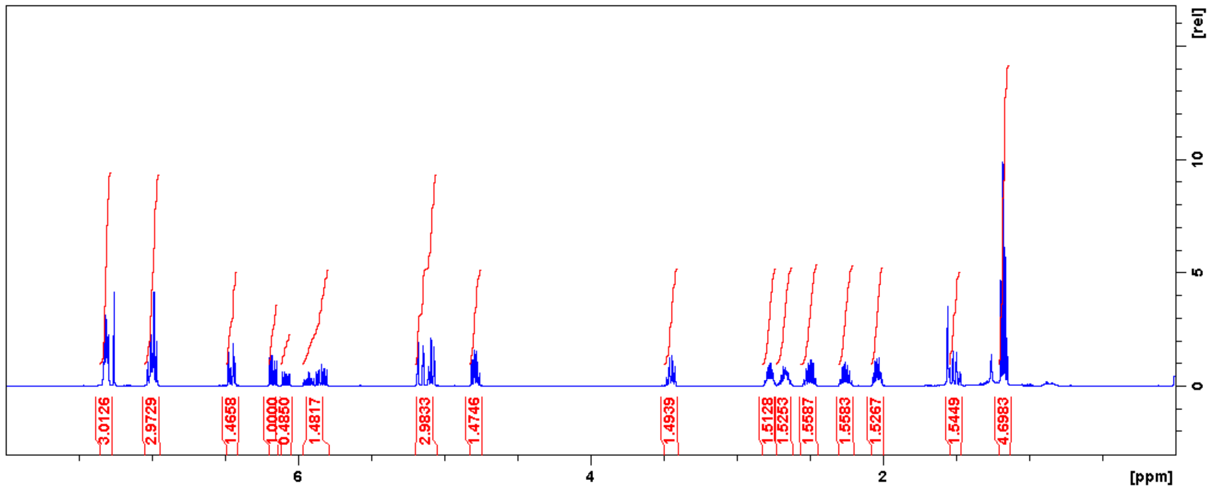

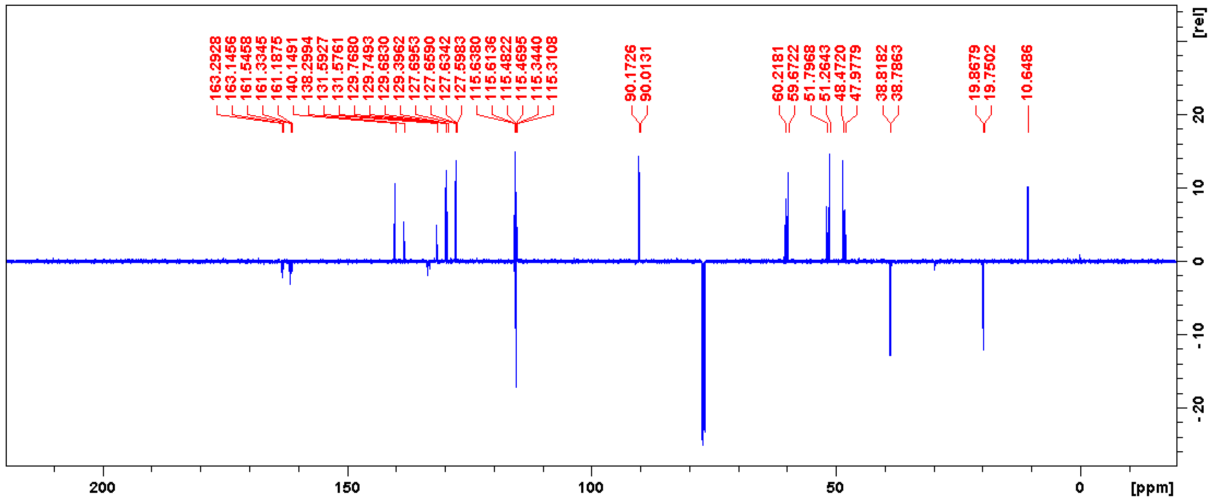

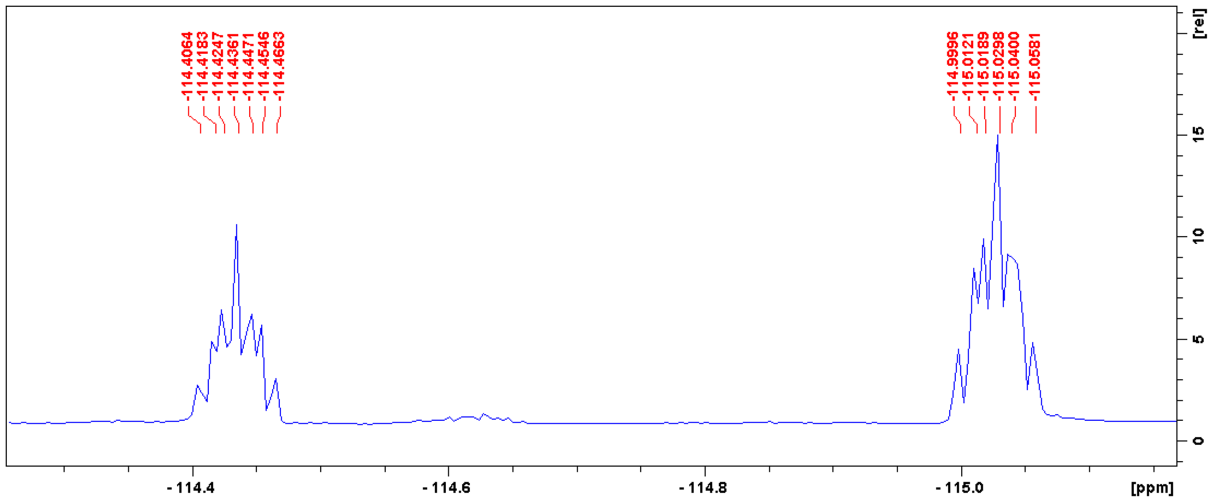

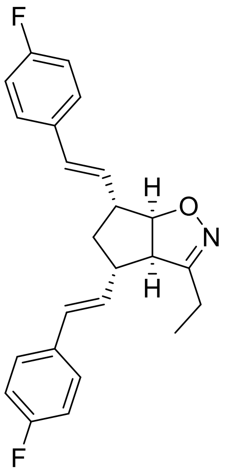

(±)-**19c**

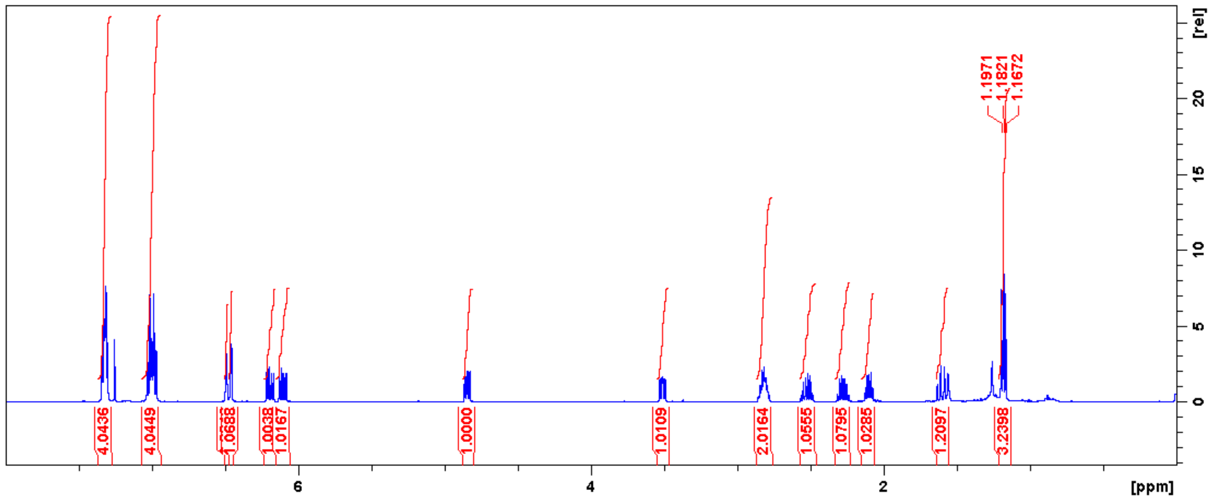

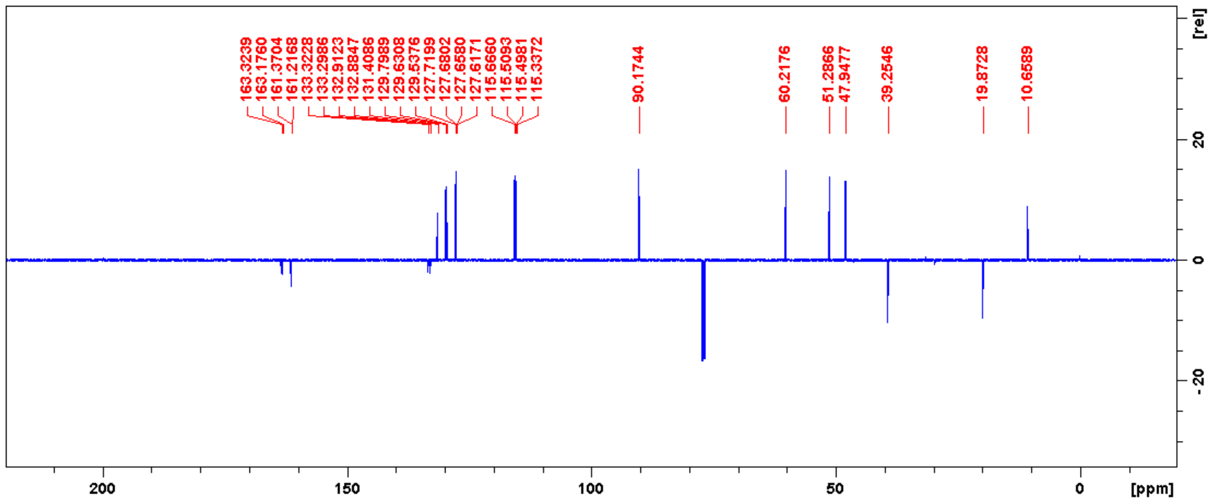

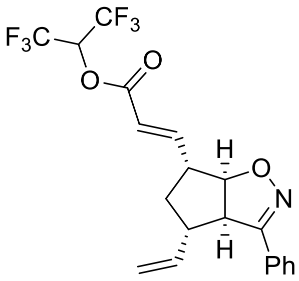
CF3
F3C
O
O
H
O
N
H
Ph

(±)-**20a**

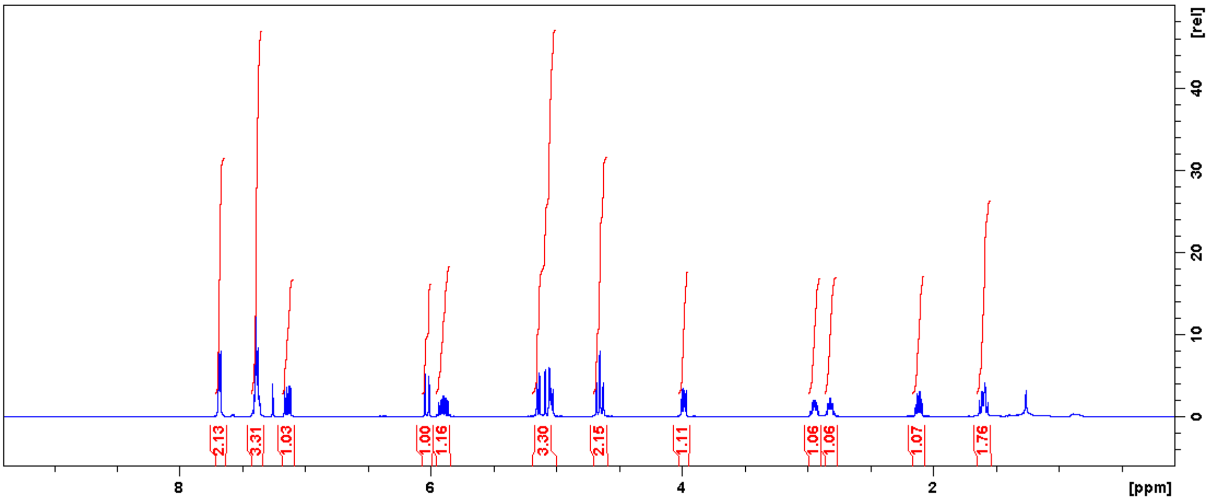
2.13
3.31
1.03
1.00
1.16
3.30
2.15
1.11
1.06
1.06
1.07
1.76
8
6
4
2
[ppm]
[rel]
0
10
20
30
40

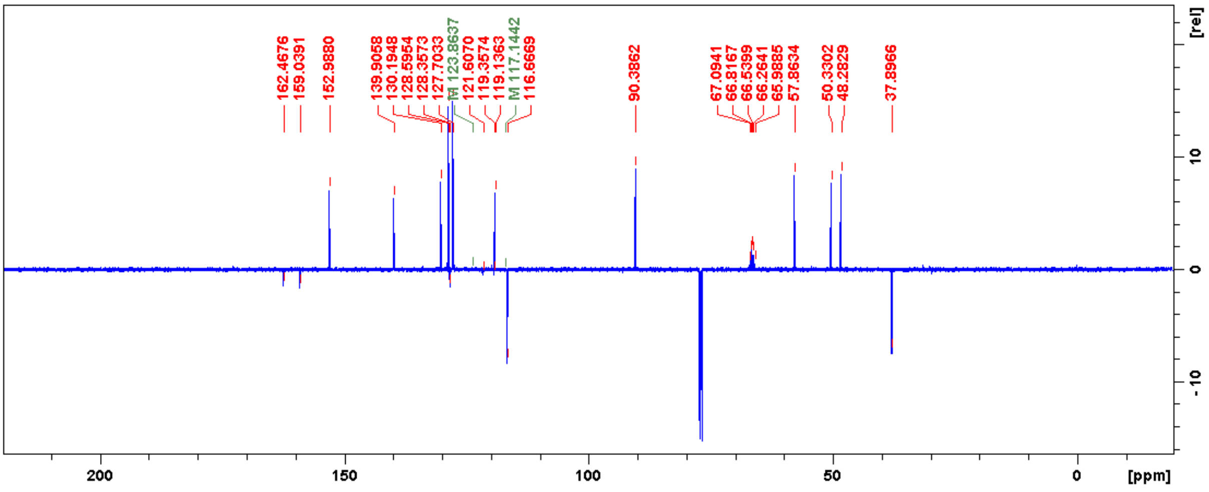
162.4676
159.0391
152.9880
139.9058
130.1948
128.5954
128.3573
127.7033
M 123.8637
121.6070
119.3574
119.1363
M 117.1442
116.6669
90.3862
67.0941
66.8167
66.5399
66.2641
65.9885
57.8634
50.3302
48.2829
37.8966
200
150
100
50
0
[ppm]
[rel]
10
0
-10

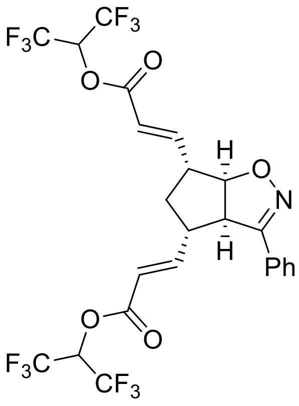

(±)-**20c**

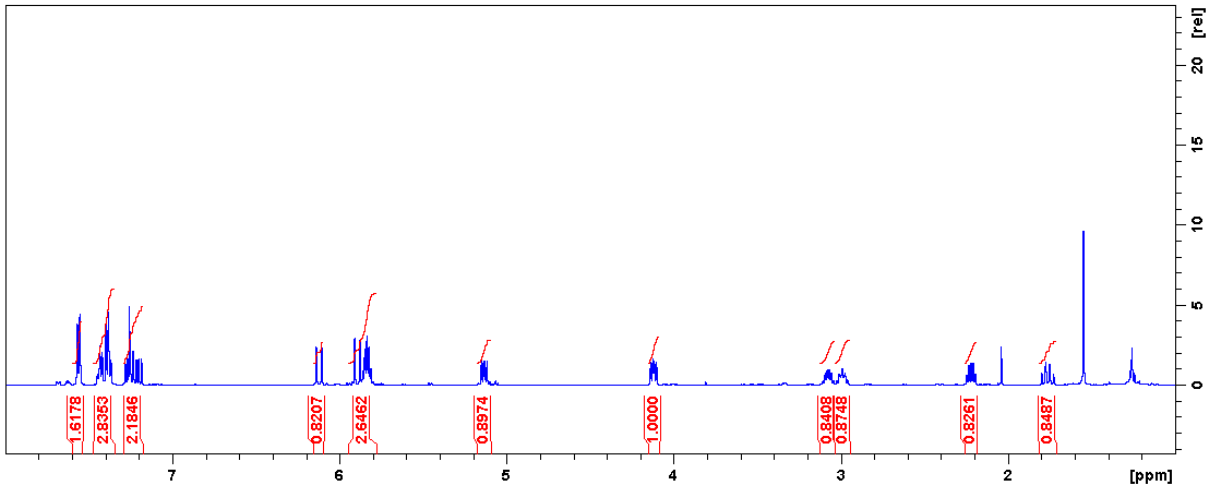

[rel]
20
15
10
5
0
1.6178
2.8353
2.1846
0.8207
2.6462
0.8974
1.0000
0.8408
0.8748
0.8261
0.8487
7
6
5
4
3
2
[ppm]

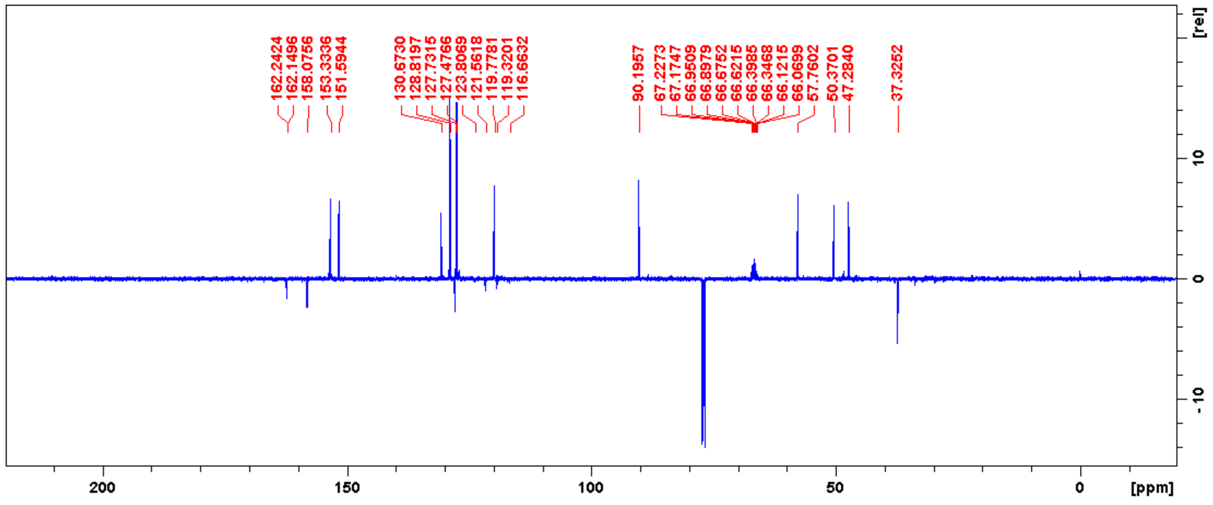

162.2424
162.1496
158.0756
153.3336
151.5944
130.6730
128.8197
127.7315
127.4766
123.8069
121.5618
119.7781
119.3201
116.6632
90.1957
67.2273
67.1747
66.9509
66.8979
66.6752
66.6215
66.3985
66.3468
66.1215
66.0699
57.7602
50.3701
47.2840
37.3252
[rel]
10
0
-10
200
150
100
50
0
[ppm]

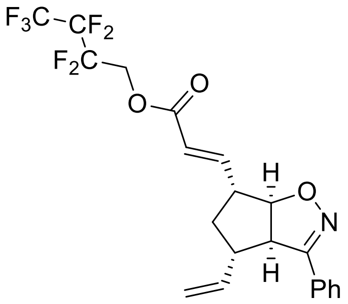

F_3C
CF_2
F_2C
O
O
H
O
N
H
Ph

(±)-**21a**

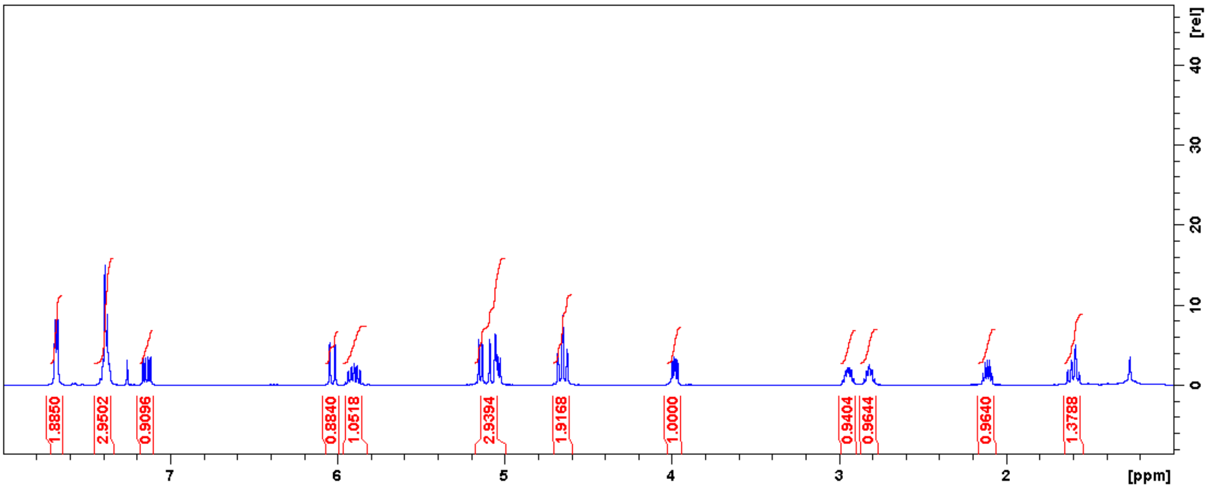

1.8850
2.9502
0.9096
0.8840
1.0518
2.9394
1.9168
1.0000
0.9404
0.9644
0.9640
1.3788
7
6
5
4
3
2
[ppm]
[rel]
40
30
20
10
0

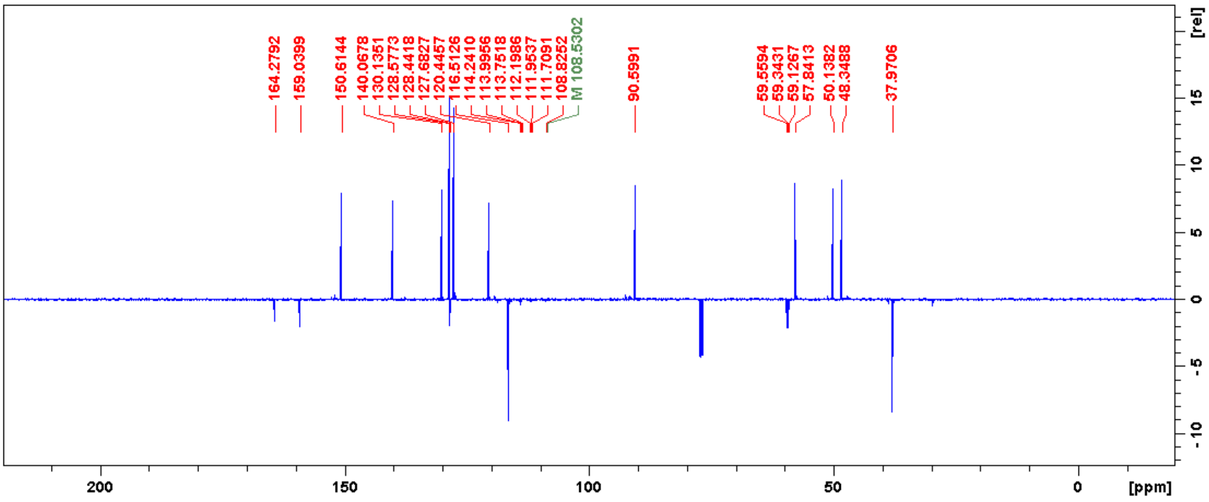

164.2792
159.0399
150.6144
140.0678
130.1351
128.5773
128.4418
127.6827
120.4457
116.5126
114.2410
113.9956
113.7518
112.1986
111.9537
111.7091
108.8252
M 108.5302
90.5991
59.5594
59.3431
59.1267
57.8413
50.1382
48.3488
37.9706
200
150
100
50
0
[ppm]
[rel]
15
10
5
0
-5
-10

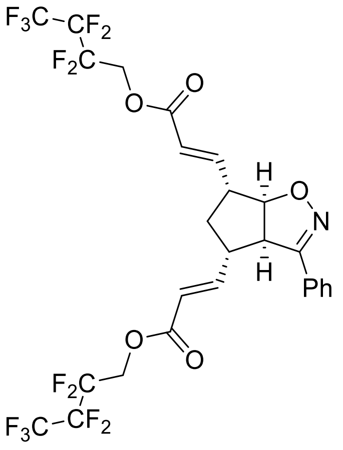
F3C
CF2
F2C
O
O
H
O
N
H
Ph
O
O
F2C
F3C
CF2

(±)-**21c**

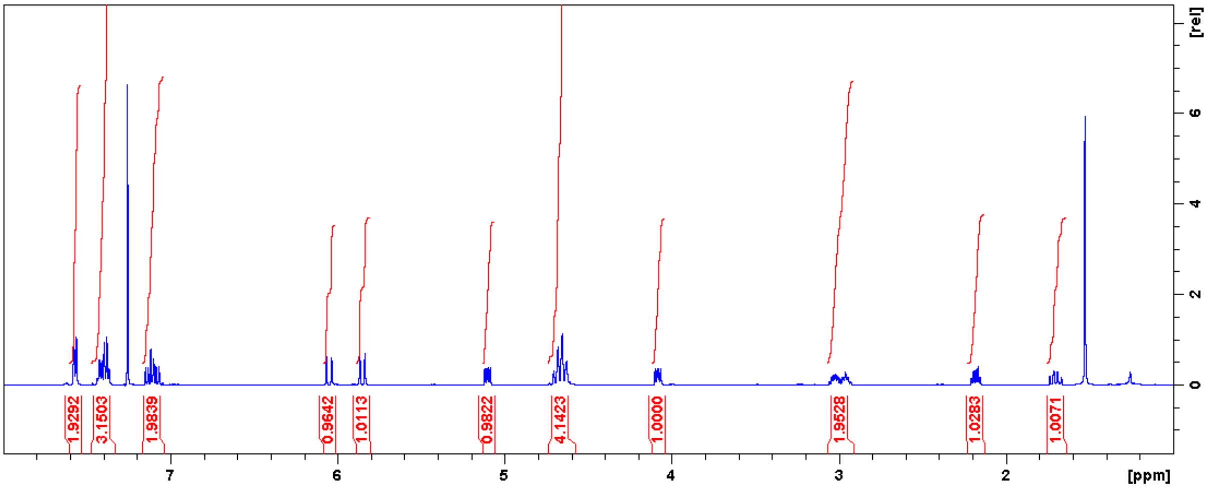
1.9292
3.1503
1.9839
0.9642
1.0113
0.9822
4.1423
1.0000
1.9528
1.0283
1.0071
[ppm]
[rel]

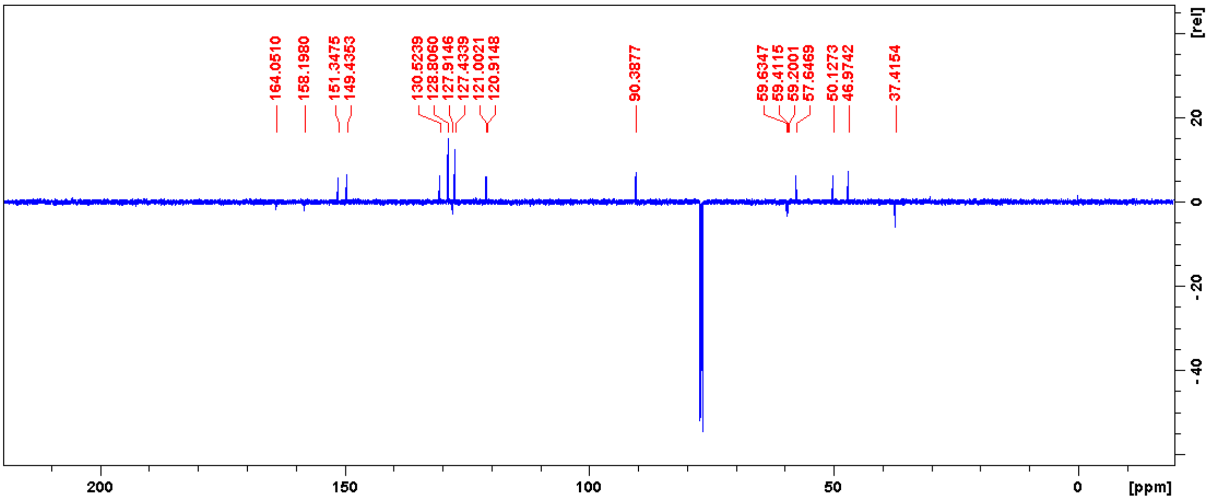
164.0510
158.1980
151.3475
149.4353
130.5239
128.8060
127.9146
127.4339
121.0021
120.9148
90.3877
59.6347
59.4115
59.2001
57.6469
50.1273
46.9742
37.4154
[ppm]
[rel]

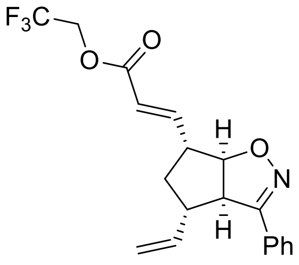

F3C
O
O
H
O
N
H
Ph

(±)-**22a**

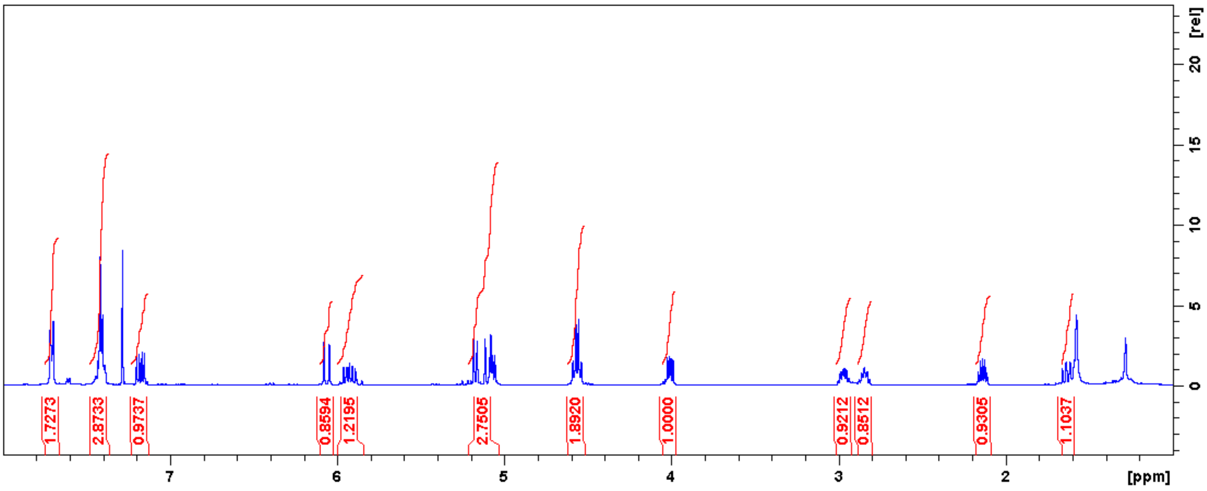

1.7273
2.8733
0.9737
0.8594
1.2195
2.7505
1.8920
1.0000
0.9212
0.8512
0.9305
1.1037
[ppm]
[rel]

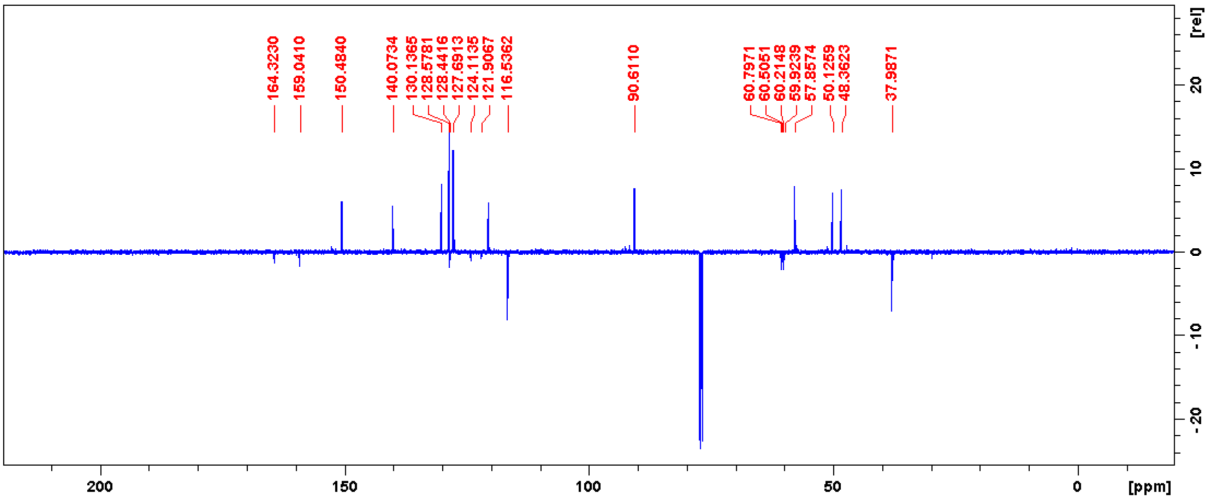

164.3230
159.0410
150.4840
140.0734
130.1365
128.5781
128.4416
127.6913
124.1135
121.9067
116.5362
90.6110
60.7971
60.5051
60.2148
59.9239
57.8574
50.1259
48.3623
37.9871
[ppm]
[rel]

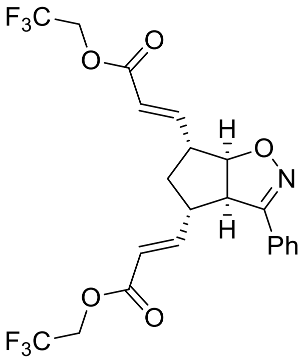

(±)-**22c**

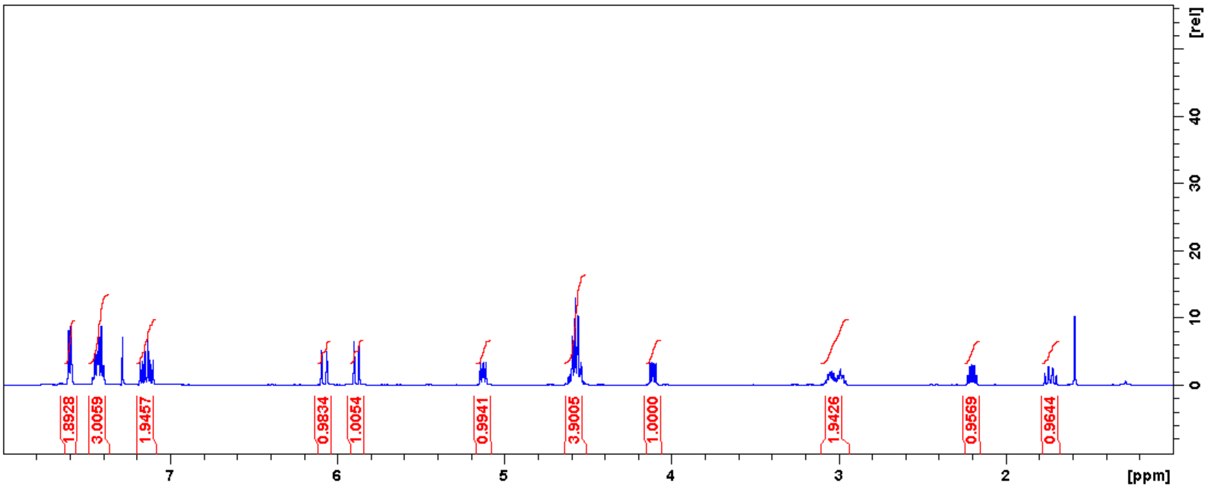

1.8928
3.0059
1.9457
0.9834
1.0054
0.9941
3.9005
1.0000
1.9426
0.9569
0.9644
[ppm]
[rel]

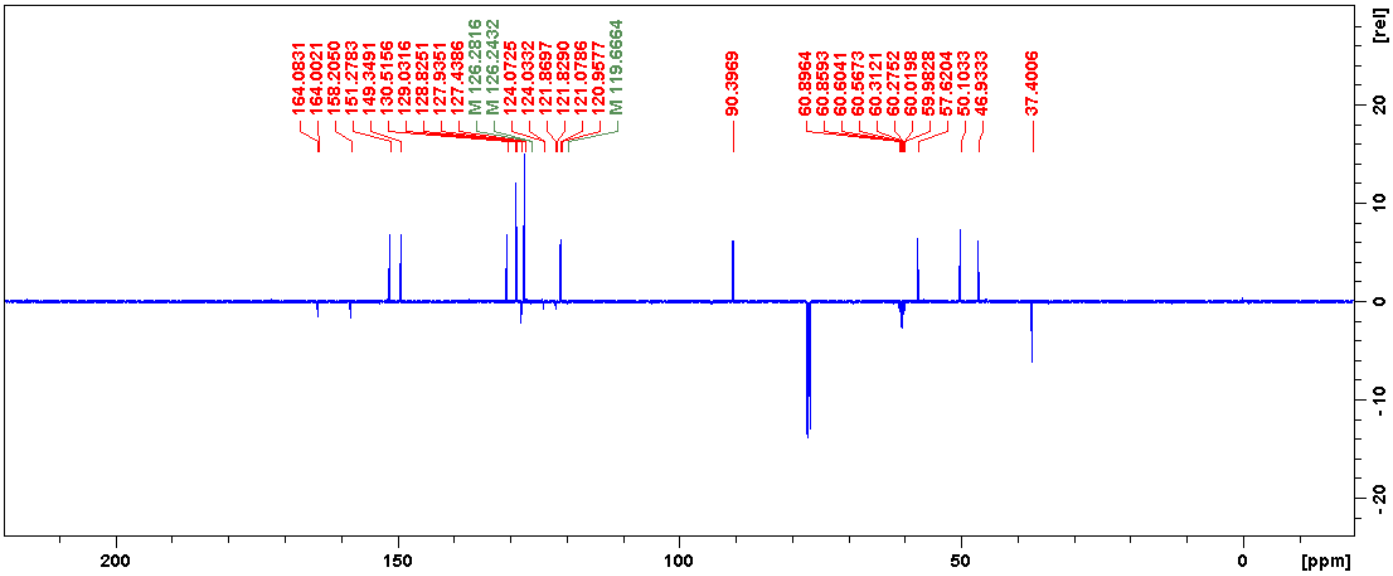

164.0831
164.0021
158.2050
151.2783
149.3491
130.5156
129.0316
128.8251
127.9351
127.4386
M 126.2816
M 126.2432
124.0725
124.0332
121.8697
121.8290
121.0786
120.9577
M 119.6664
90.3969
60.8964
60.8593
60.6041
60.5673
60.3121
60.2752
60.0198
59.9828
57.6204
50.1033
46.9333
37.4006
[ppm]
[rel]

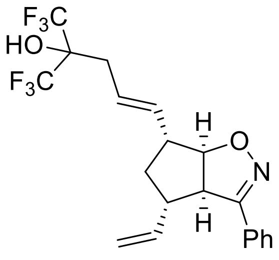
F3C
HO
F3C
H
O
N
H
Ph

(±)-23a

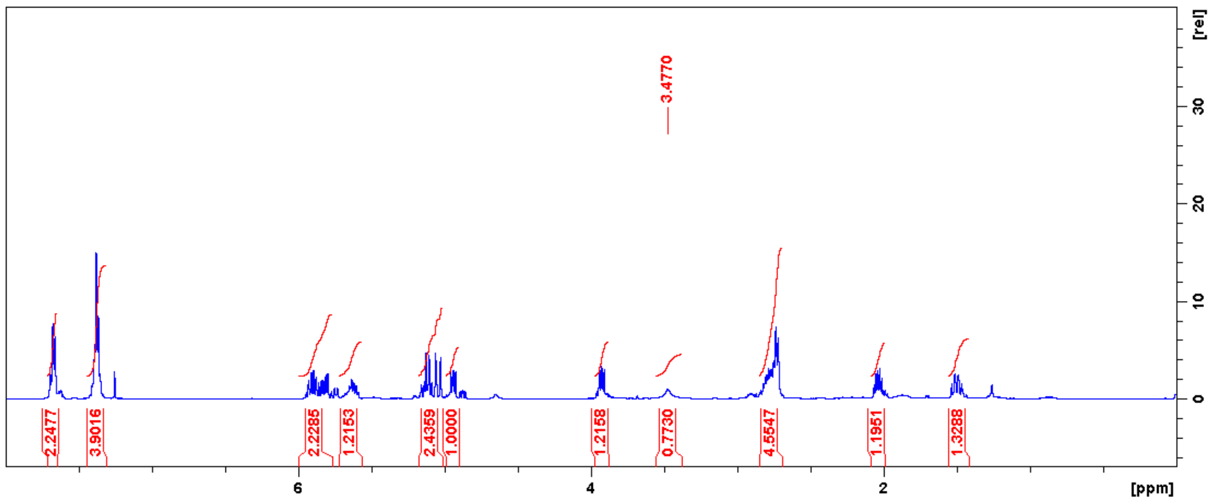
3.4770
2.2477
3.9016
2.2285
1.2153
2.4359
1.0000
1.2158
0.7730
4.5547
1.1951
1.3288
6
4
2
[ppm]
[rel]
30
20
10
0

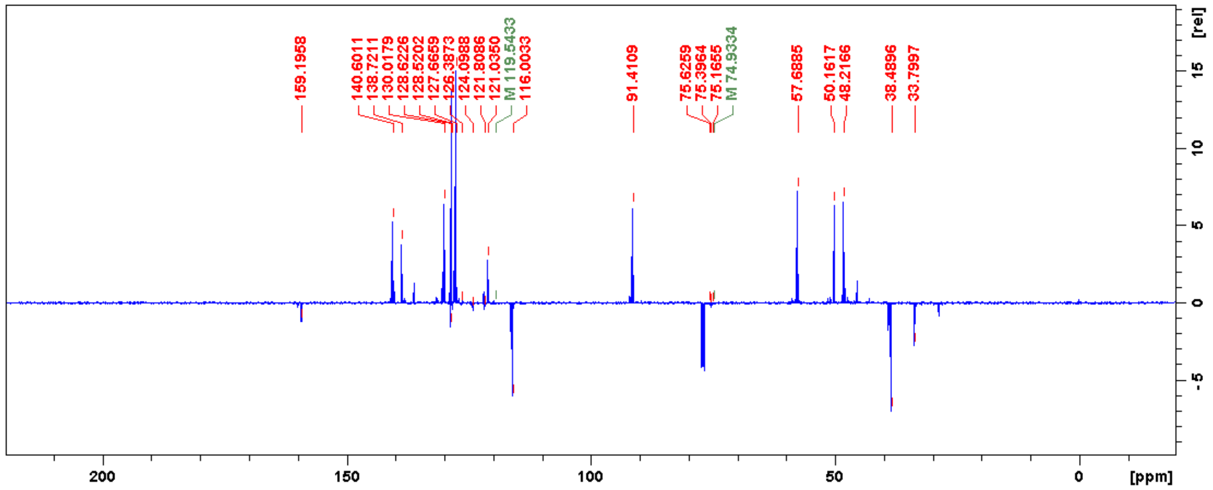
159.1958
140.6011
138.7211
130.0179
128.6226
128.5202
127.6659
126.3873
124.0988
121.8086
121.0350
M 119.5433
116.0033
91.4109
75.6259
75.3964
75.1655
M 74.9334
57.6885
50.1617
48.2166
38.4896
33.7997
200
150
100
50
0
[ppm]
[rel]
15
10
5
0
-5

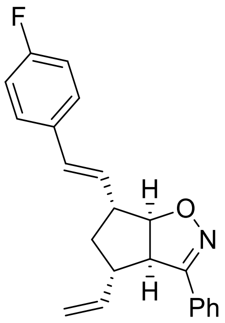
F
H
O
N
H
Ph

(±)-**25a**

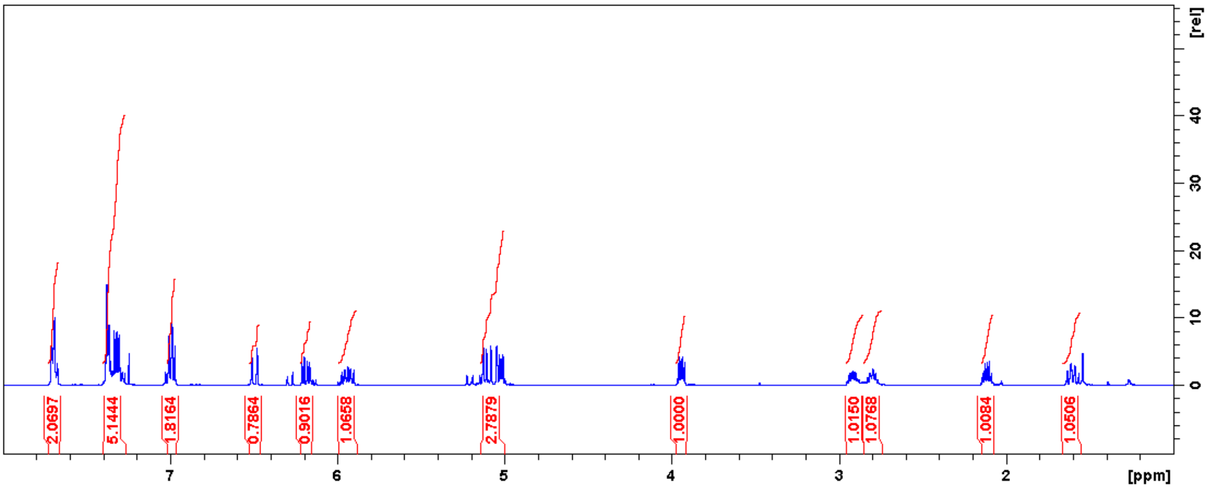
2.0697
5.1444
1.8164
0.7864
0.9016
1.0658
2.7879
1.0000
1.0150
1.0768
1.0084
1.0506
7
6
5
4
3
2
[ppm]
[rel]
0
10
20
30
40

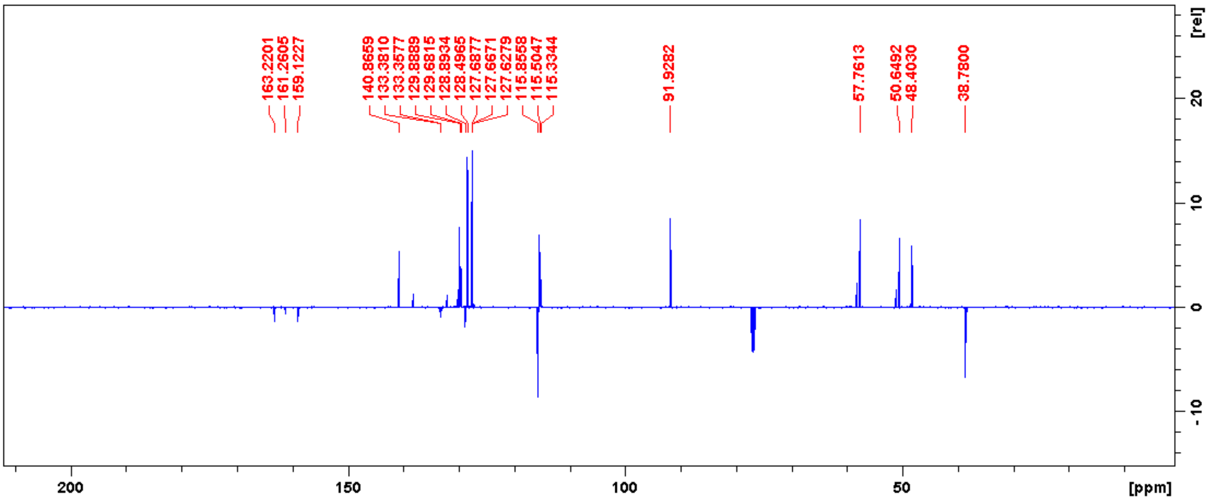
163.2201
161.2605
159.1227
140.8659
133.3810
133.3577
129.8889
129.6815
128.8934
128.4965
127.6877
127.6671
127.6279
115.8558
115.5047
115.3344
91.9282
57.7613
50.6492
48.4030
38.7800
200
150
100
50
[ppm]
[rel]
20
10
0
-10

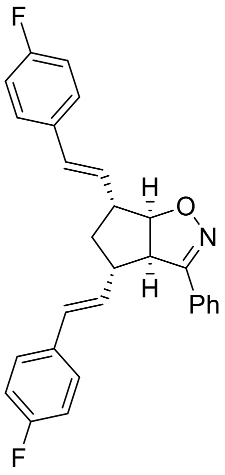
F
H
O
N
H
Ph
F

(±)-25c

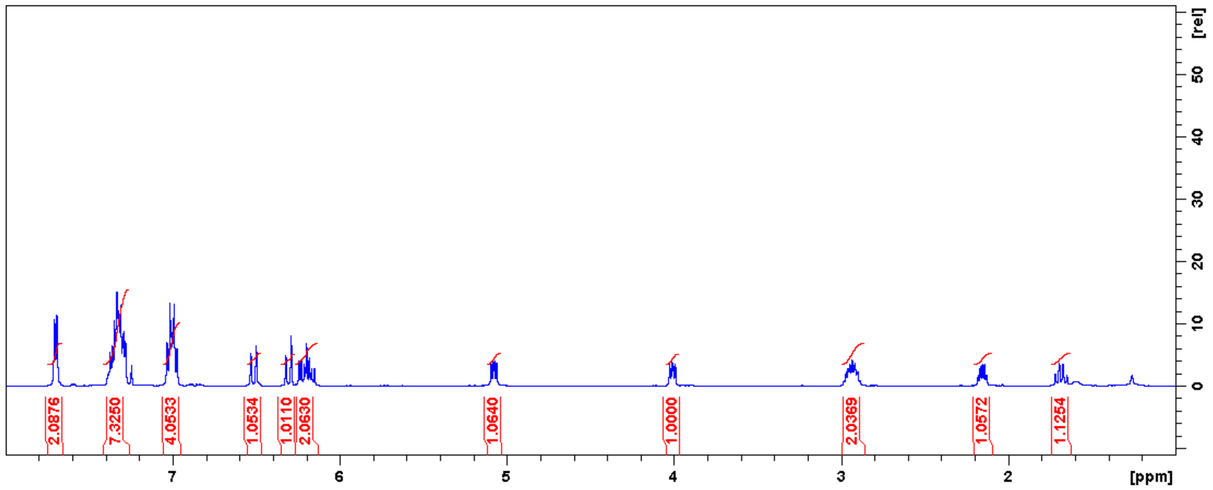
[rel]
50
40
30
20
10
0
2.0876
7.3250
4.0533
1.0534
1.0110
2.0630
1.0640
1.0000
2.0369
1.0572
1.1254
7
6
5
4
3
2
[ppm]

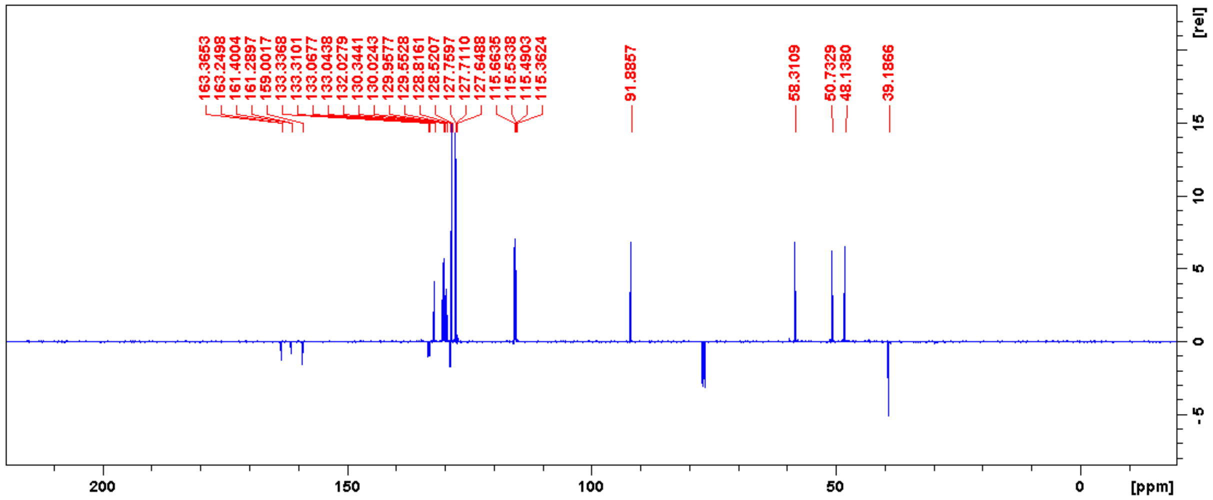
163.3653
163.2498
161.4004
161.2897
159.0017
133.3368
133.3101
133.0677
133.0438
132.0279
130.3441
130.0243
129.9577
129.5528
128.8161
128.5207
127.7597
127.7110
127.6488
115.6635
115.5338
115.4903
115.3624
91.8857
58.3109
50.7329
48.1380
39.1866
[rel]
15
10
5
0
-5
200
150
100
50
0
[ppm]

References

1. Benke, Z.; Remete, A. M.; Semghouli, A.; Kiss, L. *Asian J. Org. Chem.* **2021,** *10*, 1184–1191. doi:10.1002/ajoc.202100147
